# Interpretable machine learning for multiclass trajectory prediction in cardiovascular-kidney-metabolic syndrome stage: development and external validation

**DOI:** 10.3389/fendo.2026.1907821

**Published:** 2026-09-04

**Authors:** Tian Zhang, Zhenxu Ning, Yangwei Fan, Zhou Zidong, Jingyi Lei, Shuangxing Du, Shuzhen He

**Affiliations:** 1 School of Physics, Xi’an Jiaotong University, Xi’an, Shaanxi, China; 2 Department of Public Health, Qinghai University Medical College, Xining, China; 3 The First Affiliated Hospital of Xi’an Jiaotong University, Xi’an, Shaanxi, China; 4 Chongqing Medical University Clinical Medicine, Chongqing, China; 5 The First Affiliated Hospital of Xi’an Medical University, Xi’an, Shaanxi, China; 6 Xining Centre for Disease Control and Prevention, Xining, Qinghai, China

**Keywords:** cardiovascular-kidney-metabolic syndrome, external validation, machine learning, SHAP, trajectory prediction

## Abstract

**Background:**

Cardiovascular-kidney-metabolic (CKM) syndrome imposes a substantial global burden, yet most studies reduce stage change to a binary outcome that cannot distinguish clinically meaningful trajectories. This study developed and externally validated a multiclass machine learning framework for predicting CKM stage trajectories.

**Methods:**

A longitudinal cohort of 2,971 adults with baseline CKM stages 0–3 was used for development, with trajectories classified as Improvement, Stable, Mild Progression and Rapid Progression. Six algorithms were compared by Macro-AUC and decision curve analysis. SHAP values identified consensus predictors for a parsimonious model, externally validated in an independent hospital-based cohort (N = 291).

**Results:**

XGBoost achieved the highest Macro-AUC (0.786, 95% CI 0.757–0.813), with the six algorithms performing comparably. For rapid-progression screening, the primary model reached a sensitivity of 0.767, a specificity of 0.739 and a negative predictive value of 0.949 at a threshold of 0.23, and identified 41.9% of rapid progressors within the highest-risk 10%. Baseline CKM stage was the dominant prognostic determinant, with a stage-only benchmark reaching 0.689. Six consensus predictors were identified: baseline CKM stage, age, fasting glucose, TyG-BMI index, systolic blood pressure and triglycerides. The parsimonious model retained over 98% of full-model discrimination and reached a Macro-AUC of 0.752 externally without retraining.

**Conclusion:**

This framework distinguishes CKM trajectories and is intended for screening rapid progression rather than assigning individuals to a single trajectory. Baseline CKM stage is the principal prognostic determinant, and the six-predictor model preserves discrimination using routinely available measurements, supporting stratified follow-up pending confirmation in larger cohorts.

## 1 Introduction

The pathological interplay among cardiovascular disease, chronic kidney disease, and metabolic disorders has increasingly emerged as a critical concern in global public health. In 2023, the American Heart Association (AHA) formally introduced the conceptual framework of cardiovascular-kidney-metabolic (CKM) syndrome, which integrates the interactions among metabolic risk factors, renal impairment, and cardiovascular pathology into a unified staging system (1, 2). A substantial body of epidemiological evidence indicates that CKM syndrome imposes a considerable disease burden worldwide. In the United States, 55.3% of adults aged ≥65 years are classified as being at stages 3–4 (3). Whereas in China, the overall prevalence of CKM stages 3–4 among individuals aged ≥45 years reaches 26.5% (4). Collectively, these findings underscore the clinical importance of stage-based identification and prediction of CKM syndrome for enabling timely intervention.

To date, a limited number of studies have applied machine learning approaches to predict specific outcomes within CKM populations, including cognitive impairment (5) and depression risk (6). However, no prior work has leveraged multiple algorithms to predict both the direction and magnitude of stage transitions in CKM syndrome. Moreover, existing studies have generally reduced CKM stage changes to a binary outcome of progression versus non-progression, an approach that fails to distinguish clinically meaningful trajectories such as improvement, stability, mild deterioration, and rapid deterioration. In contrast, a multiclass prediction framework can simultaneously capture the direction and magnitude of change, thereby providing more refined risk stratification to inform clinical decision-making (7). Furthermore, the incorporation of interpretability techniques such as SHapley Additive exPlanations (SHAP) allows for the quantitative characterization of each predictor’s contribution to model output, enhancing clinical utility. Ensemble learning and stacking strategies have demonstrated superior discriminative performance compared with single models in cardiovascular risk prediction (8, 9), yet they have not been systematically applied to the prediction of CKM stage trajectories.

Accordingly, this study utilized longitudinal cohort data from China to define CKM stage changes as four distinct trajectories, namely improvement, stability, mild progression, and rapid progression, and developed a multiclass prediction framework incorporating six algorithms. Through multidimensional classification performance evaluation and decision curve analysis, combined with SHAP-based cross-trajectory feature importance analysis and the construction of parsimonious models, this study aimed to identify robust predictors of CKM stage trajectories and to characterize how feature contributions differ across trajectory categories.

## 2 Materials and methods

### 2.1 Study population and outcome definition

This study was conducted using data from the China Health and Retirement Longitudinal Study (CHARLS), a nationally representative survey covering 28 provinces in China that follows community-dwelling residents aged ≥45 years every two to three years (10). Given the availability of blood-based biomarker data, Wave 1 (2011) and Wave 3 (2015) were merged to construct a longitudinal analytic cohort. Eligible participants attended both waves and could be assigned a CKM stage at each (n = 3,442). Staging required body mass index, waist circumference, blood pressure, fasting plasma glucose, glycated hemoglobin, lipid profile and serum creatinine, together with self-reported diabetes, hypertension, dyslipidemia, chronic kidney disease, stroke and heart disease. Participants were excluded if any component was missing at either wave, if the stage was indeterminate, or if the inter-wave interval could not be established. Participants at CKM stage 4 at baseline were excluded (n = 471), leaving 2,971 (**Supplementary Figure 1A**). Stage 4 is the terminal category of the staging system, so no progression class is attainable from it. The target population is therefore adults at stages 0 to 3. CKM staging was defined according to the 2023 American Heart Association Presidential Advisory on cardiovascular-kidney-metabolic syndrome, which stratifies individuals into stages 0 through 4 based on the presence and severity of metabolic risk factors, chronic kidney disease, and subclinical or clinical cardiovascular disease (1, 2).

The primary outcome was a four-class trajectory defined by the magnitude of change in CKM stage. The change value was calculated by subtracting the Wave 1 stage from the Wave 3 stage, and each participant was assigned to one of the following trajectories accordingly. A value less than 0 was classified as Improvement (stage regression), 0 as Stable (no change), 1 as Mild Progression (one-stage increase), and 2 or greater as Rapid Progression (two or more stage increases). Because the outcome is a change in stage, the attainable classes depend on the baseline stage. Improvement cannot occur from stage 0, as no lower stage exists, and rapid progression cannot occur from stage 3, as stage 4 is the ceiling of the staging system and a two-stage increase would require a stage that does not exist. The study was designed, analyzed and reported in accordance with the TRIPOD+AI statement.

### 2.2 Candidate predictor variables

All candidate predictors were derived from Wave 1 baseline data and spanned multiple domains, including sociodemographic characteristics (age, sex, marital status, residence type, and household registration), socioeconomic position (annual household income), lifestyle factors (smoking, alcohol consumption, physical activity, and sleep duration), anthropometric measurements (height, weight, waist circumference, and body mass index), physical function measures (grip strength, gait speed, and peak expiratory flow), blood biomarkers (complete blood count, renal function, lipid profile, glucose, and inflammatory markers), composite metabolic indices (TyG and TyG-BMI), comorbidities (chronic diseases across multiple systems), cognitive function and depressive symptom scores. Detailed variable definitions and the measurement type declared for each predictor are provided in **Supplementary Table 1**. Baseline CKM stage was also included as a predictor. To prevent data leakage, variables directly involved in defining CKM stage, including diagnoses and constituent components of hypertension, diabetes, dyslipidemia, heart disease, stroke, and chronic kidney disease, were excluded from the predictor set.

### 2.3 Data preprocessing

Variables missing in more than 20% of participants were excluded, and missingness for the 60 retained predictors is reported in **Supplementary Table 1**. The dataset was partitioned into training and testing sets at an 8:2 ratio using stratified random sampling, before any imputation or standardization. Missing values in continuous variables were imputed using the training-set median, and binary and ordinal variables using the training-set mode. All imputation values were computed solely from the training set and applied unchanged to the testing set and to the external cohort (11). After imputation, continuous predictors were transformed via z-score standardization based on training-set means and standard deviations, with the same parameters applied to the testing set. Binary variables were not standardized (12, 13). Model development, hyperparameter tuning, and preprocessing were performed exclusively on the training set.

### 2.4 Class imbalance handling

Given the substantial imbalance across the four trajectory classes, an inverse-frequency weighting strategy was adopted (14). The weight of each observation was defined as w = N/(K × n_c), where N is the training-set size, K the number of classes and n_c the size of the class to which that observation belongs, so that the weights average one and the effective sample size equals the actual sample size. These weights were applied to all six algorithms through a single shared fitting path and were recomputed within each cross-validation fold from that fold’s training partition only. The support vector machine was fitted with the e1071 implementation (**Supplementary Table 2**). The class weights are reported in **Supplementary Table 3**, and unweighted fitting is retained as a sensitivity analysis. Seven further strategies were compared, namely no adjustment, SMOTE, SMOTE with edited nearest neighbors, ADASYN, random undersampling, a balanced random forest and *post hoc* cost-sensitive thresholds, with all resampling performed inside the training folds only.

### 2.5 Machine learning model construction

Six models were developed for four-class trajectory prediction, comprising five base algorithms spanning different algorithmic families, namely elastic net regularized multinomial logistic regression (Multinomial LR) (15), random forest (RF) (16), extreme gradient boosting (XGBoost) (17), gradient boosting machine (GBM) (18) and support vector machine with radial basis function kernel (SVM), together with a two-layer stacking ensemble. Hyperparameters for each base algorithm were optimized through five-fold cross-validation using the macro-averaged AUC as the selection criterion, and the random seed was uniformly set to 2026. A single stratified five-fold partition was generated once and shared by every algorithm and by the stacking layer. The complete search space and the selected values are given in **Supplementary Table 4**.

The stacking ensemble used RF, XGBoost and GBM as first-level base learners, each fitted with the hyperparameters selected for it above. Out-of-fold prediction probabilities from the same five-fold partition formed a 12-column meta-feature matrix, comprising four class probabilities from each of the three base learners, which was used to train an elastic net regularized multinomial logistic regression as the second-level meta-learner (19, 20).

### 2.6 Model evaluation

Discriminative performance was assessed on the independent testing set. AUC was computed for each trajectory class using a one-versus-rest approach. The macro-averaged AUC, the principal discriminative metric, is the unweighted mean of the four class-specific AUCs, and the macro-averaged receiver operating characteristic curve is obtained by vertical averaging of the four class-specific curves on a common false-positive-rate grid. The micro-averaged AUC, which pools all participant-by-class pairs into a single curve, is reported separately. Classification metrics included overall accuracy, balanced accuracy, Cohen’s kappa, class-specific and macro- and weighted-average F1 scores, log loss, sensitivity, and specificity, with F1 set to zero for a class that exists in the evaluation set but is never predicted. The 95% confidence intervals for all key metrics were derived from 1,000 bootstrap resamples. The algorithm attaining the highest macro-averaged AUC in the internal testing set was pre-specified as the primary model and was used for the SHAP, threshold and external analyses.

Calibration was first assessed on the untransformed predicted probabilities by grouping the testing set into deciles and comparing the mean predicted probability with the observed proportion, and by reporting the calibration slope, calibration-in-the-large, the observed-to-expected ratio and the Brier score (21). Recalibration was then performed by Platt scaling and, separately, by isotonic regression, each fitted exclusively on out-of-fold predictions from the training set and applied unchanged to the testing set and to the external cohort. Decision curve analysis was conducted for each of the four trajectory classes across clinically relevant threshold probability ranges to evaluate net benefit relative to the treat-all and treat-none strategies (22, 23).

The intended clinical use is the identification of participants at risk of rapid progression for closer follow-up. Threshold-dependent performance for the rapid-progression class was therefore reported at the Youden-optimal threshold, at the lowest thresholds achieving a sensitivity of at least 0.90 and 0.80, and at the threshold equal to the observed prevalence, with sensitivity, specificity, positive and negative predictive value, the counts of true and false positives and negatives, the proportion of the cohort flagged, and the number needed to screen at each point.

### 2.7 Feature importance and model interpretation

SHAP values were employed to quantify the contribution of each feature to model predictions (24). SHAP values were computed separately for each of the four trajectory classes using the built-in predcontrib function on all training-set participants (25). For each class, beeswarm plots displaying the twelve highest-ranked predictors were generated to illustrate both feature importance and directional effects. A hierarchically clustered heatmap of mean absolute SHAP values was constructed, together with an UpSet plot to visualize the intersection patterns of the top 15 features across the four classes (26). In the heatmap the mean absolute SHAP value is shown on a logarithmic scale with no within-row or within-column rescaling. SHAP values quantify how much each variable moves the predicted log-odds of a class in the fitted model relative to that class’s base value. They describe the behavior of the model and are not estimates of causal effects.

### 2.8 Parsimonious model construction

To evaluate the trade-off between model complexity and predictive performance, a parsimonious model was built based on consensus features, defined as predictors ranked within the top 15 SHAP values across all four trajectory classes (11). All six models were retrained using this reduced feature set, with hyperparameter tuning procedures kept consistent with those of the full models.

### 2.9 Dependence on baseline CKM stage

Because baseline CKM stage is both a predictor and a determinant of which outcome classes are attainable, three specifications were compared to separate its contribution from that of the remaining predictors. First, a baseline-stage-only benchmark assigned each participant the conditional class frequencies observed for their baseline stage in the training set. Second, all six models were refitted after removing baseline stage from the predictor set. Third, conditional models were fitted within each baseline-stage stratum, retaining a class if it had at least ten training and at least one testing observation; within-stratum macro averages were computed over the classes attainable in that stratum.

### 2.10 External validation

To evaluate the generalizability of the parsimonious model in a real-world clinical setting, a retrospective cohort was constructed using administrative health records provided by the Xining Municipal Center for Disease Control and Prevention, encompassing inpatient, outpatient, and examination data from the Third People’s Hospital of Xining between 2016 and 2022. This study was approved by the Ethics Committee of the Xining Municipal Center for Disease Control and Prevention (approval No. 2023-LLPJ-02). Patients aged ≥45 years at baseline, matching the age eligibility of the development cohort, with at least two recorded assessments separated by at least 12 months and complete CKM staging data at the first and the last assessment were eligible, and those with a baseline CKM stage of 4 were excluded, yielding a final analytic sample of 291 (**Supplementary Figure 1B**). Trajectories were classified using the same four-category scheme as the primary analysis, recomputed from the recorded first and last stages.

Because a subset of predictors was not available in the hospital records, the parsimonious model was pre-specified as the primary external analysis, with the full model retained as a sensitivity analysis. All six parsimonious models were applied to the external cohort without retraining, using preprocessing parameters and recalibration mappings frozen from the CHARLS training set, and discrimination, calibration and decision curve analyses were conducted using identical procedures to those of the primary analysis. The internal-to-external macro-averaged AUC drop was defined as a transportability index.

### 2.11 Sensitivity analysis

Six sensitivity analyses were conducted, addressing missing-value handling, outcome definition, predictor completeness in external validation, and external-cohort follow-up interval. To evaluate the impact of missing-value handling, two analyses were conducted using XGBoost as the representative model. The first adopted a complete-case analysis, in which participants with any missing predictor were excluded before repeating the entire modeling pipeline. The second employed multiple imputation by chained equations (MICE) (27, 28), in which continuous variables were imputed using predictive mean matching and binary variables using logistic regression, with the imputation model fitted on the training rows only and then applied to the testing rows; models were trained separately on each imputed dataset, and the mean and range of Macro-AUC across the 10 imputations were used as the point estimate and uncertainty measure, respectively. To evaluate the impact of outcome definition, two further analyses were conducted: the four classes were collapsed to three by combining mild and rapid progression into any progression, and rapid progression was redefined under an alternative rule. To evaluate the effect of predictor completeness on external performance, the external validation was repeated using the full-predictor models, in which the predictors not measured in the hospital records were assigned their development-cohort imputation values. To evaluate the impact of follow-up interval, the external validation analysis was repeated restricting to patients whose follow-up interval approximated the fixed four-year CHARLS interval.

In addition, learning curves were constructed to establish whether the models had converged and to quantify optimism. Each model was refitted on nested random subsamples of the training set from 20% to 100% of its size, with five repeats at each size, and the training, cross-validated and testing Macro-AUC were recorded at each size. Optimism was defined as the training Macro-AUC minus the cross-validated Macro-AUC at the same size.

## 3 Results

### 3.1 Study sample and CKM stage transitions

A total of 2,971 participants with baseline CKM stages 0 to 3 were included in the final analysis. In the complete matched sample (n = 3,442), the CKM stage transition matrix (**Supplementary Table 5**; **Supplementary Figure 2A**) showed that participants at CKM stages 0 and 1 predominantly transitioned toward higher stages, with 39.9% of stage 0 individuals progressing to stage 2 and 46.9% of stage 1 individuals progressing to stage 2. Among those at stage 2, 67.5% remained at the same stage, whereas among stage 3 participants, 70.3% maintained their original stage and 14.7% progressed to stage 4. No participant at stage 4 (n = 471) was observed at a lower stage at Wave 3, and these participants were excluded (**Supplementary Table 6**).

**Figure 1:**
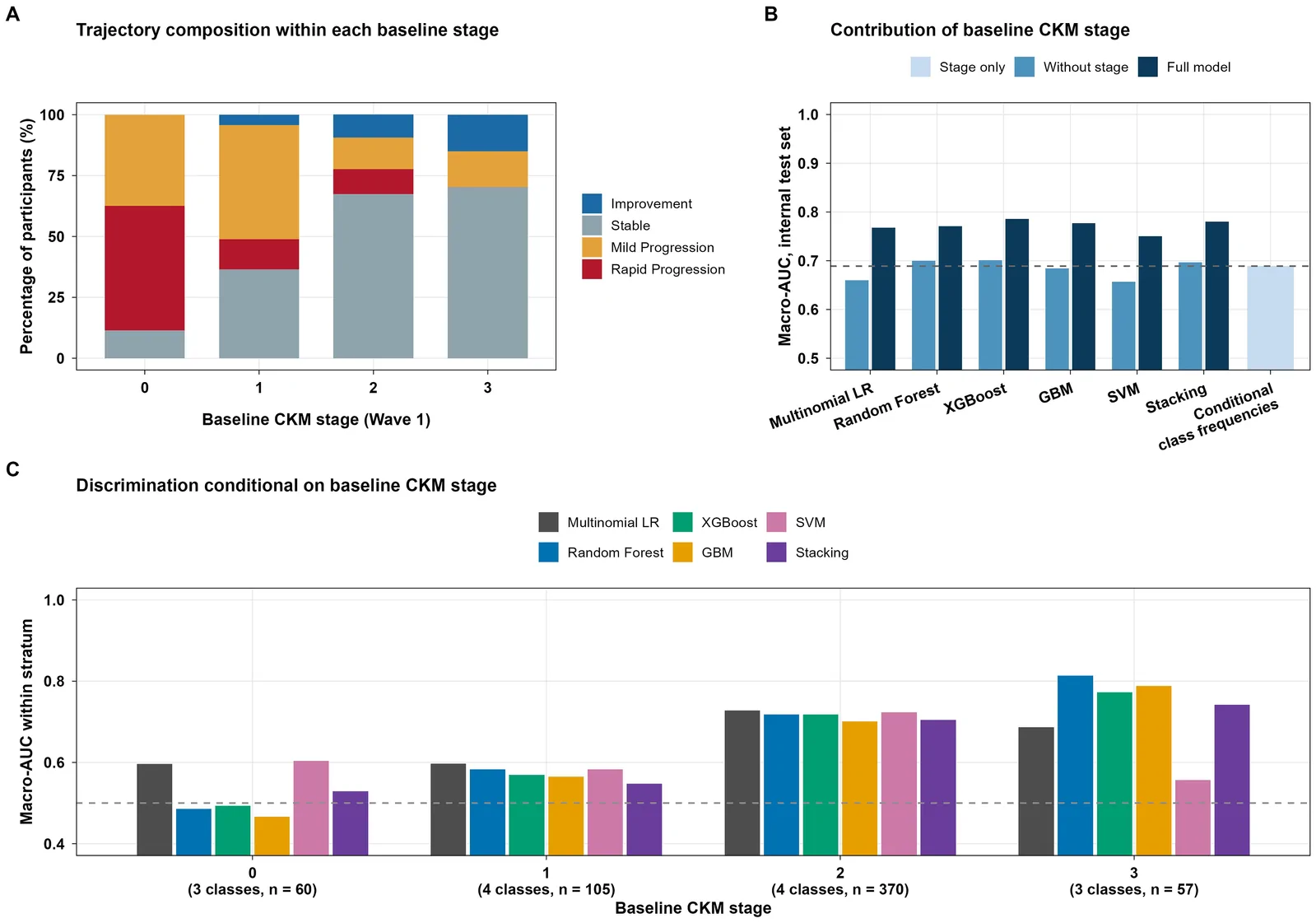
Dependence of model performance on baseline CKM stage. **(A)** Trajectory class composition within each baseline stage. **(B)** Macro-AUC under three specifications: baseline stage alone, all predictors except baseline stage, and the full model. The dashed line marks the stage-only benchmark. **(C)** Macro-AUC of models fitted separately within each baseline stage stratum, with baseline stage excluded. The dashed line marks chance.

Based on the direction and magnitude of stage transitions, participants were classified into four CKM trajectories (**Supplementary Table 7**): Improvement (n = 235, 7.9%), Stable (n = 1,649, 55.5%), Mild Progression (n = 654, 22.0%), and Rapid Progression (n = 433, 14.6%). The attainable class space varied by baseline stage: no participant at stage 0 could improve (**Supplementary Table 6**; **Figure 1A**). Stable accounted for 12% of participants at stage 0, 68% at stage 2 and 70% at stage 3; Rapid Progression accounted for 51% at stage 0 and none at stage 3. The training set comprised 2,379 participants and the testing set 592, with trajectory class proportions preserved across both sets (testing set: Improvement n = 47, Stable n = 329, Mild Progression n = 130, Rapid Progression n = 86).

### 3.2 Model discrimination

**Figure 2:**
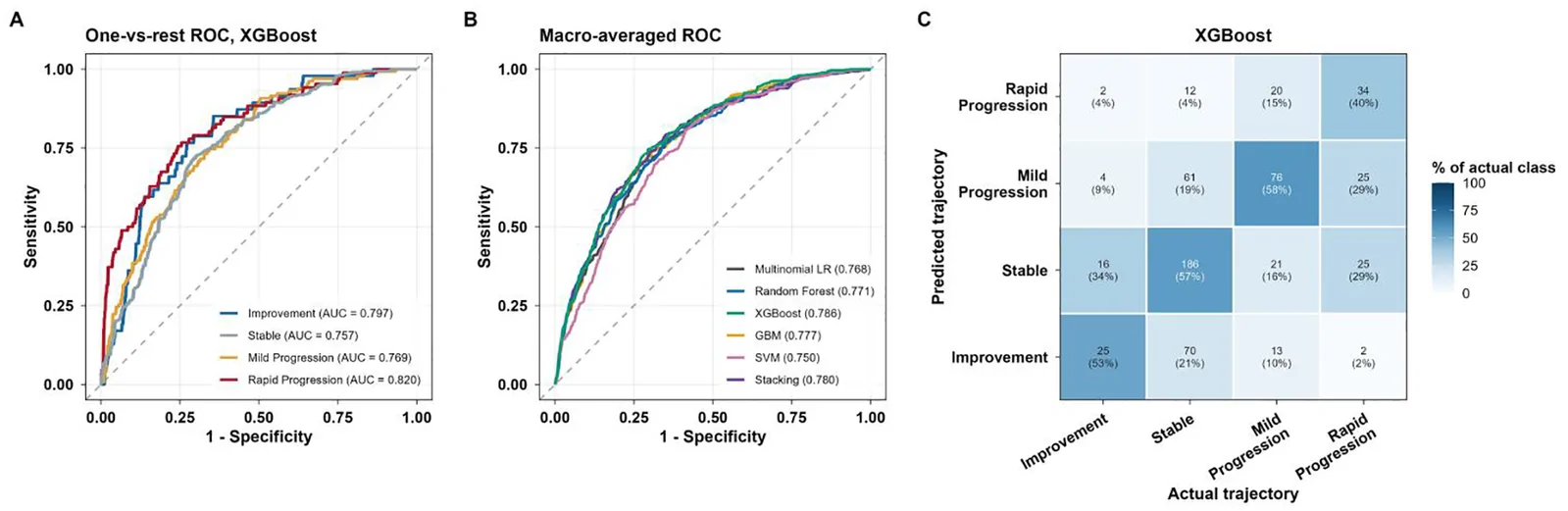
Discrimination of six machine learning models for four-class CKM trajectory prediction in the internal testing set (N = 592). **(A)** One-versus-rest receiver operating characteristic curves for each trajectory class, for the XGBoost model. **(B)** Macro-averaged ROC curves for all six models, obtained by interpolating each class-specific curve onto a common false-positive-rate grid and averaging sensitivities across the four classes. **(C)** Confusion matrix for XGBoost. Rows indicate the predicted trajectory and columns the actual trajectory, with percentages computed within the true class, so that each column sums to 100%.

All six models discriminated better than chance across the four trajectory classes. Macro-averaged AUC on the independent testing set was 0.786 (95% CI 0.757 to 0.813) for XGBoost, 0.780 (0.749 to 0.808) for Stacking, 0.777 (0.747 to 0.806) for GBM, 0.771 (0.741 to 0.801) for Random Forest, 0.768 (0.737 to 0.795) for Multinomial LR and 0.750 (0.720 to 0.778) for the support vector machine (**Table 1**, **Figure 2B**). XGBoost, which attained the highest macro-averaged AUC, was accordingly designated the primary model. The confidence intervals overlap substantially, and DeLong tests comparing XGBoost with the stacking ensemble were non-significant for all four classes (P = 0.28 to 0.91) (**Supplementary Table 8**). Four of the twenty DeLong comparisons with XGBoost reached nominal significance, all favoring XGBoost: the Improvement class against the random forest (P = 0.011) and against the support vector machine (P = 0.010), the Rapid Progression class against GBM (P = 0.041) and the Mild Progression class against the support vector machine (P = 0.044). Micro-averaged AUC, which pools all participant-by-class pairs, exceeded macro-averaged AUC for every model, ranging from 0.749 to 0.842, and was highest for the support vector machine, the model with the lowest macro-averaged AUC (**Supplementary Table 8**; **Supplementary Figure 3**).

**Table 1:** Multiclass performance in the internal testing set and the external cohort.

Model	Macro-AUC	Overall accuracy	Balanced accuracy	Cohen’s κ	Macro F1	Brier score
Internal testing set (n = 592), full-predictor model (60 predictors)						
Multinomial LR	0.768 (0.737–0.795)	0.466 (0.429–0.507)	0.525 (0.478–0.568)	0.270 (0.222–0.319)	0.443 (0.402–0.481)	0.656 (0.640–0.671)
Random forest	0.771 (0.741–0.801)	0.627 (0.589–0.664)	0.501 (0.452–0.549)	0.376 (0.314–0.437)	0.516 (0.465–0.566)	0.566 (0.543–0.586)
XGBoost	0.786 (0.757–0.813)	0.542 (0.503–0.583)	0.519 (0.466–0.571)	0.321 (0.265–0.376)	0.480 (0.434–0.522)	0.593 (0.569–0.616)
GBM	0.777 (0.747–0.806)	0.541 (0.503–0.581)	0.518 (0.468–0.568)	0.315 (0.261–0.370)	0.481 (0.437–0.523)	0.597 (0.570–0.622)
SVM	0.750 (0.720–0.778)	0.603 (0.566–0.642)	0.336 (0.310–0.363)	0.228 (0.177–0.282)	0.321 (0.286–0.356)	0.524 (0.486–0.561)
Stacking	0.780 (0.749–0.808)	0.463 (0.426–0.502)	0.529 (0.478–0.575)	0.270 (0.220–0.321)	0.444 (0.400–0.484)	0.678 (0.668–0.689)
External cohort (n = 291), parsimonious model (6 predictors)						
Multinomial LR	0.713 (0.670–0.755)	0.474 (0.416–0.533)	0.419 (0.364–0.479)	0.243 (0.165–0.325)	0.403 (0.338–0.469)	0.651 (0.623–0.679)
Random forest	0.752 (0.707–0.793)	0.515 (0.457–0.570)	0.475 (0.409–0.546)	0.294 (0.212–0.375)	0.469 (0.402–0.534)	0.603 (0.568–0.637)
XGBoost	0.742 (0.699–0.782)	0.467 (0.412–0.522)	0.445 (0.379–0.519)	0.240 (0.168–0.316)	0.419 (0.359–0.484)	0.636 (0.607–0.665)
GBM	0.735 (0.692–0.778)	0.454 (0.395–0.512)	0.428 (0.359–0.500)	0.214 (0.140–0.292)	0.397 (0.330–0.460)	0.651 (0.611–0.690)
SVM	0.728 (0.684–0.773)	0.543 (0.481–0.595)	0.399 (0.358–0.439)	0.292 (0.209–0.373)	0.392 (0.345–0.434)	0.606 (0.556–0.656)
Stacking	0.751 (0.707–0.790)	0.467 (0.412–0.522)	0.459 (0.385–0.536)	0.243 (0.173–0.316)	0.420 (0.357–0.484)	0.673 (0.657–0.689)

Values are point estimates computed on the full evaluation set, with 95% confidence intervals from 1,000 bootstrap resamples. Macro-AUC is the unweighted mean of the four one-versus-rest class AUCs. Balanced accuracy is the macro-averaged sensitivity across classes. Macro F1 is averaged over all four classes, with F1 set to zero where a class exists in the evaluation set but is never predicted. Kappa is Cohen’s kappa. Brier score is the multiclass score, for which lower is better. The internal testing set rows are the full-predictor model (60 predictors) and the external cohort rows the parsimonious model (6 predictors), which was pre-specified as the primary external analysis; the two blocks therefore describe different models and are not a like-for-like internal-versus-external comparison. No model parameter, preprocessing constant or recalibration function was re-estimated in the external cohort.

AUC, area under the receiver operating characteristic curve; CI, confidence interval; GBM, gradient boosting machine; LR, logistic regression; SVM, support vector machine; XGBoost, extreme gradient boosting; κ, Cohen’s kappa.

Class-specific discrimination followed the same ordering in every model, with the two extreme trajectories the most separable and the two intermediate trajectories the least. For XGBoost the class-specific AUCs were 0.820 for Rapid Progression, 0.797 for Improvement, 0.769 for Mild Progression and 0.757 for Stable (**Supplementary Table 8**; **Figure 2A**).

### 3.3 Classification performance

Overall accuracy on the internal testing set ranged from 0.463 to 0.627 and balanced accuracy from 0.336 to 0.529 (**Table 1**). For XGBoost, 321 of 592 participants were assigned to the correct trajectory (overall accuracy 0.542) and the mean of the four class-specific sensitivities was 0.519, against a macro-averaged AUC of 0.786 (**Figure 2C**).

For XGBoost, class-specific sensitivity was 58.5% for Mild Progression (76 of 130), 56.5% for Stable (186 of 329), 53.2% for Improvement (25 of 47) and 39.5% for Rapid Progression (34 of 86) (**Figure 2C**). Errors concentrated between adjacent classes. Of the 329 participants who were Stable, 61 (19%) were assigned to Mild Progression and 70 (21%) to Improvement, and of the 86 who progressed rapidly, 25 (29%) were assigned to Mild Progression and 25 (29%) to Stable. Misclassification between the two oppositely directed extremes affected 2 of 47 Improvement participants and 2 of 86 Rapid Progression participants.

The support vector machine attained the second-highest overall accuracy (0.603) together with the lowest balanced accuracy (0.336) and the lowest macro-F1 (0.321). The random forest achieved the highest overall accuracy (0.627) and the highest macro-F1 (0.516), whereas the stacking ensemble achieved the highest balanced accuracy (0.529) on the lowest overall accuracy (0.463). Per-model, per-class sensitivity, specificity, predictive values and F1 are given in **Supplementary Table 8** and the corresponding confusion matrices in **Supplementary Figure 4**.

### 3.4 Calibration

**Figure 3:**
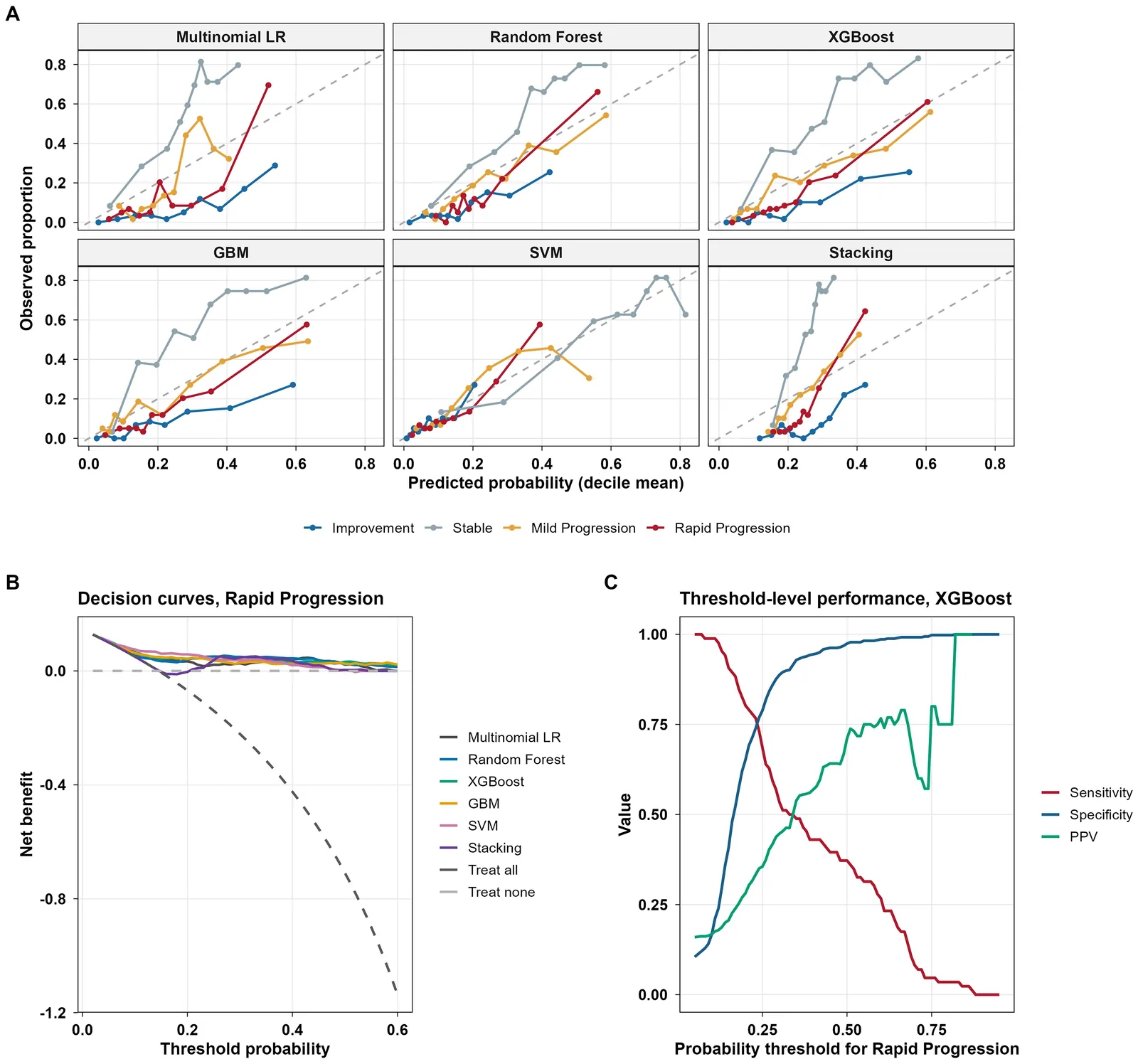
Calibration and clinical utility in the internal testing set (N = 592). **(A)** Calibration curves for all six models, plotting the observed proportion against the mean predicted probability within deciles of predicted risk. The curves show raw predicted probabilities: Platt and isotonic recalibration were fitted on out-of-fold training predictions only, so no curve in this panel has been fitted to the data against which it is plotted. **(B)** Decision curves for the Rapid Progression class, the pre-specified primary use case, showing net benefit against threshold probability for each model together with the treat-all and treat-none reference strategies. **(C)** Sensitivity, specificity and positive predictive value for Rapid Progression as a function of the probability threshold, computed one-versus-rest, for XGBoost.

Calibration of the untransformed predicted probabilities showed a consistent pattern across all six models (**Figure 3A**; **Supplementary Table 9**). For the Stable class the observed proportion exceeded the mean predicted probability throughout most of the predicted range, reaching an observed proportion above 0.80 in the highest deciles of several models, while for the Improvement class the observed proportion fell below the predicted probability. The two progression classes lay closer to the identity line.

Before recalibration the calibration slopes ranged from 0.814 to 3.823 and the observed-to-expected ratios from 0.304 to 2.145, with the stacking ensemble the most severely miscalibrated. After Platt scaling the slopes fell within 0.815 to 1.356 and the observed-to-expected ratios within 0.872 to 1.069 for every model and class, and the multiclass Brier score improved from 0.524 to 0.678 down to 0.503 to 0.523. Macro-averaged AUC moved by no more than 0.007 after recalibration.

### 3.5 Clinical utility and threshold-level performance

Decision curve analysis for the Rapid Progression class showed positive net benefit for all six models across the threshold range of approximately 0.02 to 0.60, exceeding both the treat-all and the treat-none reference strategies throughout (**Figure 3B**). The corresponding curves for the other three classes are given in **Supplementary Figure 5**. Operating points for the rapid-progression class are reported in **Figure 3C**; **Supplementary Table 10**. Net benefit for the Improvement class was negative at all four operating points, and decision curve analysis therefore does not support using the model to identify that class (**Supplementary Table 10**; **Supplementary Figure 5**).

Using XGBoost, the Youden-optimal threshold of 0.23 gave a sensitivity of 0.767 and a specificity of 0.739, identifying 66 of the 86 rapid progressors in the testing set while flagging 198 of 592 participants (33.4%). Positive predictive value was 0.333, negative predictive value 0.949 and the number needed to screen 3.0. Raising sensitivity to 0.907 required lowering the threshold to 0.15, which flagged 63.9% of the cohort and reduced positive predictive value to 0.206 (**Supplementary Table 10**).

Screening the highest-ranked 10% of the cohort by predicted probability identified 41.9% of all rapid progressors, a 4.2-fold enrichment over random selection, and screening 30% identified 72.1% (**Supplementary Table 10**; **Supplementary Figure 6**).

### 3.6 SHAP feature contribution by trajectory class

**Figure 4:**
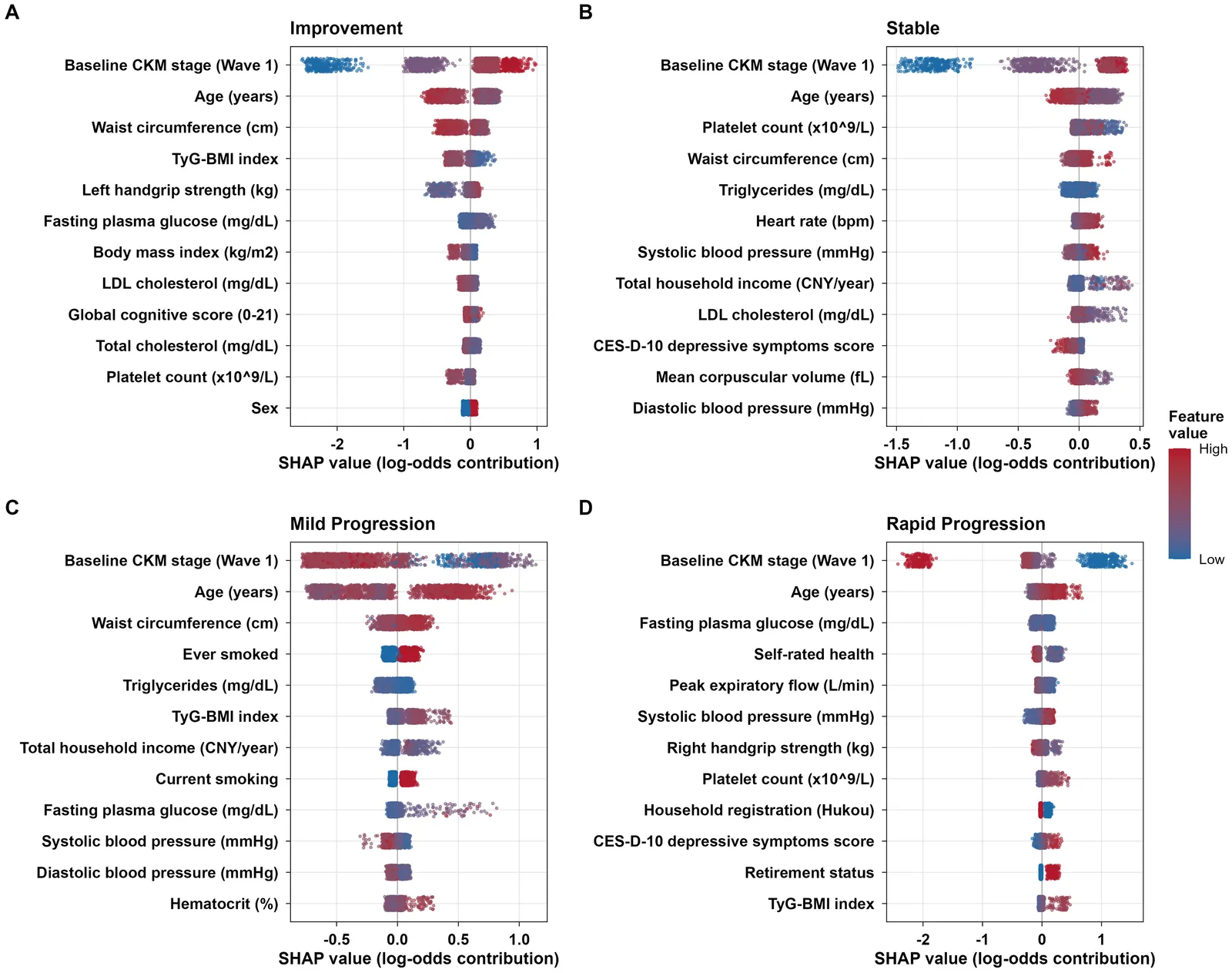
SHAP beeswarm plots showing the contribution of individual predictors for each CKM trajectory class, based on the class-weighted XGBoost model fitted on the training set. **(A)** Improvement. **(B)** Stable. **(C)** Mild progression. **(D)** Rapid progression. The twelve highest-ranked predictors are shown per class, ordered by mean absolute SHAP value.

Baseline CKM stage was the highest-ranked predictor for all four trajectory classes and age the second-highest, and the direction of the baseline-stage contribution reversed between the improvement and the progression classes (**Figure 4**; **Supplementary Table 11**).

For Improvement (**Figure 4A**), higher baseline stages carried strongly positive SHAP values and the lowest stage strongly negative ones, and older age contributed negatively. Waist circumference, TyG-BMI index, fasting plasma glucose and body mass index ranked third to sixth, with higher values of the adiposity and glycemic measures contributing negatively to the predicted probability of improvement. LDL cholesterol, total cholesterol, systolic blood pressure, left handgrip strength, sex and triglycerides completed the twelve shown.

For Stable (**Figure 4B**), baseline CKM stage again contributed positively at higher stages, consistent with the stratum composition in **Figure 1A**. Age, triglycerides, platelet count and systolic blood pressure ranked second to fifth, followed by body weight, diastolic blood pressure, mean corpuscular volume, heart rate, waist circumference, fasting plasma glucose and the TyG-BMI index.

For Mild Progression (**Figure 4C**), the direction of the baseline-stage contribution reversed, with lower baseline stages contributing positively. Age ranked second and a smoking history entered third, with ever having smoked and, in seventh place, currently smoking both contributing positively. Fasting plasma glucose, triglycerides, diastolic blood pressure, the TyG-BMI index, waist circumference, platelet count, LDL cholesterol and body weight completed the panel.

For Rapid Progression (**Figure 4D**), the SHAP distribution for baseline CKM stage was the widest of any predictor in any class, with the lowest stages contributing most positively, and older age contributed positively. Self-rated health ranked third and fasting plasma glucose fourth, followed by peak expiratory flow, systolic blood pressure, the CES-D-10 score, platelet count, right handgrip strength, hemoglobin, sleep duration and the episodic memory score. A self-rated health measure, a respiratory measure, a depressive-symptom score, sleep duration and a cognitive measure ranked among the twelve highest for this class and were largely absent from the other three.

### 3.7 Feature contribution across classes

**Figure 5:**
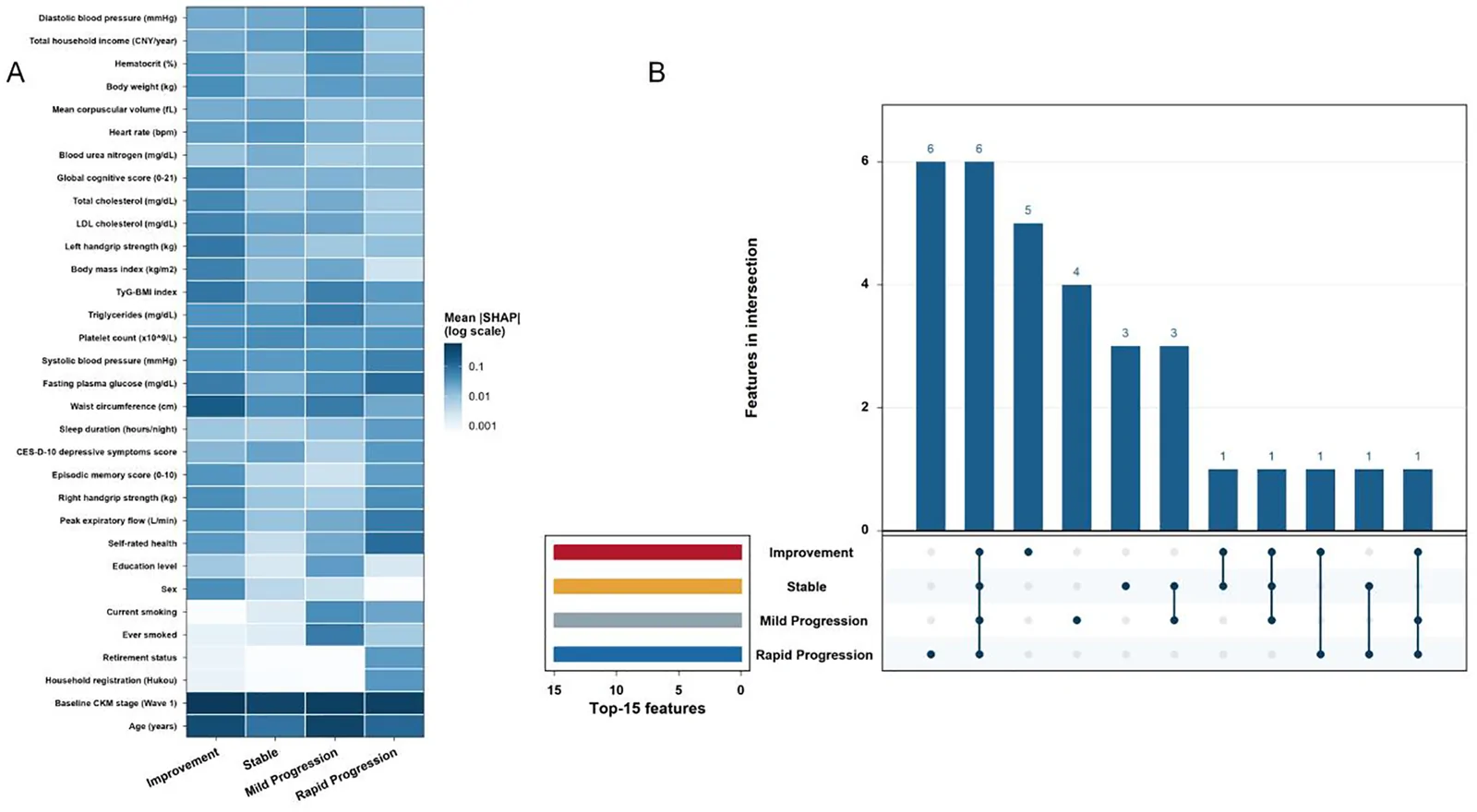
Feature contribution across trajectory classes. **(A)** Heatmap of mean absolute SHAP values for predictors ranked in the top 15 of at least one class. Color is on a logarithmic scale, labelled on the original scale. Rows are ordered by hierarchical clustering. **(B)** UpSet plot of the intersection pattern of the top 15 SHAP predictors across the four classes. Bars give the number of predictors in each intersection.

The heatmap of mean absolute SHAP values on a common logarithmic scale is shown in **Figure 5A**. Baseline CKM stage was the largest contributor in every class and age the second largest, and both were an order of magnitude above the remaining predictors. Smoking contributed negligibly to Improvement and Stable but substantially to Mild Progression. Household registration contributed negligibly to the first three classes but substantially to Rapid Progression, as did sleep duration, self-rated health and the depressive-symptom score. Waist circumference contributed most to Improvement. Sex contributed least to Rapid Progression.

The UpSet plot of the top-15 intersections (**Figure 5B**) shows that the union of the four top-15 sets comprises 29 predictors. Six predictors were ranked in the top 15 of all four trajectory classes and constitute the consensus set used for the parsimonious model (**Supplementary Table 11**). Three predictors were shared by exactly three classes and seven by exactly two. The number of predictors unique to a single class was four for Improvement, two for Stable, two for Mild Progression and five for Rapid Progression.

### 3.8 Dependence on baseline CKM stage

A benchmark assigning each participant the conditional class frequencies of their baseline stage achieved a macro-averaged AUC of 0.689 (**Supplementary Table 12**). Removing baseline stage from the predictor set gave 0.657 to 0.701, below this benchmark for three of the six models. Adding baseline stage back raised macro-averaged AUC by 0.071 to 0.108, whereas the 59 remaining predictors contributed between −0.032 and 0.012 relative to the benchmark (**Figure 1B**).

Within-stratum macro-averaged AUC, fitted separately in each baseline stage, was 0.466 to 0.604 at stage 0, 0.548 to 0.597 at stage 1, 0.701 to 0.728 at stage 2 and 0.557 to 0.814 at stage 3 (**Figures 1A, C**; **Supplementary Table 13**). These averages are taken over three attainable classes at stages 0 and 3 and four at stages 1 and 2. At stage 2 XGBoost predicted rapid progression for no participant, giving a sensitivity and a positive predictive value of zero for that class (**Supplementary Table 14**).

### 3.9 Performance of the parsimonious model

The parsimonious model, built on the six consensus predictors, lost less than 0.02 of macro-averaged AUC relative to the full model for every algorithm (**Supplementary Table 15**; **Supplementary Figure 7**). Only for the support vector machine was the parsimonious model the better of the two (0.778 against 0.750), whereas for Multinomial LR the two were equivalent (0.767 against 0.768). Overall accuracy moved in both directions, rising from 0.603 to 0.652 for the support vector machine and falling from 0.627 to 0.566 for the random forest. Reducing the predictor set from 60 variables to 6 retained more than 98% of full-model discrimination.

### 3.10 External validation

In the external clinical cohort (N = 291) the trajectory distribution was Stable (n = 111, 38.1%), Mild Progression (n = 106, 36.4%), Rapid Progression (n = 55, 18.9%) and Improvement (n = 19, 6.5%) (**Supplementary Figure 2B**), against 55.5% Stable and 22.0% Mild Progression in the development cohort. Baseline characteristics and standardized mean differences between the two cohorts are given in **Supplementary Table 16**; **Supplementary Figure 8**. The follow-up interval was shorter and more variable than the fixed four-year development interval (median 1.96 years) (**Supplementary Table 17**; **Supplementary Figure 9**).

**Figure 6:**
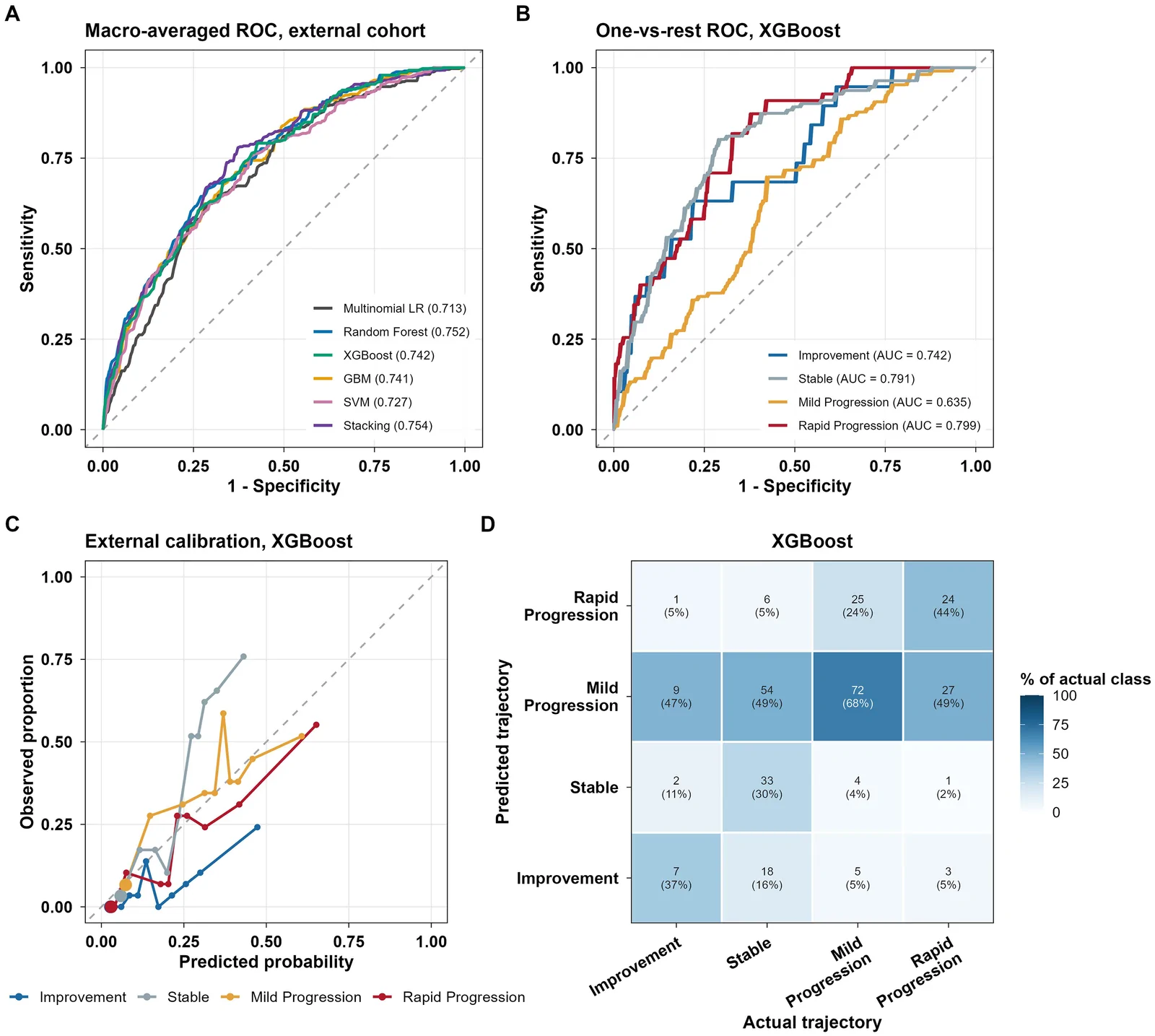
External validation of the parsimonious model in the independent hospital cohort (N = 291). **(A)** Macro-averaged ROC curves for all six frozen models. **(B)** One-versus-rest ROC curves for XGBoost. **(C)** Calibration curves for XGBoost; point size is proportional to the number of participants in the decile. **(D)** Confusion matrix for XGBoost, with rows indicating the predicted trajectory and columns the actual trajectory and percentages computed within the true class, so that each column sums to 100%. No model parameter, preprocessing constant or recalibration function was re-estimated externally: all were frozen from the CHARLS training set.

All six frozen parsimonious models retained moderate discrimination externally (**Figure 6A**; **Supplementary Table 18**). Macro-averaged AUC was 0.752 for the random forest, 0.751 for stacking, 0.742 for XGBoost, 0.735 for GBM, 0.728 for the support vector machine and 0.713 for Multinomial LR. The full models reached 0.672 to 0.738. The external cohort measured 5 of the 6 parsimonious predictors and 46 of the 60 full-model predictors (**Supplementary Table 17**).

For the primary model, class-specific external AUC was 0.798 for Rapid Progression, 0.789 for Stable, 0.746 for Improvement and 0.636 for Mild Progression (**Figure 6B**). Classification degraded, with 136 of 291 participants assigned to the correct trajectory (overall accuracy 0.467) and a mean of the four class-specific sensitivities of 0.443, highest for Mild Progression (67.9%, 72 of 106) and lowest for Stable (29.7%, 33 of 111) (**Figure 6D**). Mild Progression was the largest predicted class externally although it was not the largest observed one.

External calibration deviations from the identity line remained for the Stable and Improvement classes (**Figure 6C**; **Supplementary Table 9**). The recalibration functions frozen from the development cohort did not transport: external calibration slopes spread from 0.317 to 1.504 and the multiclass Brier score did not improve. Internal-to-external macro-averaged AUC fell for every parsimonious model, by 0.010 for the random forest to 0.054 for Multinomial LR (**Supplementary Table 15**; **Supplementary Table 18**). Restricting the external cohort to the 76 participants whose follow-up interval fell between 3.0 and 5.0 years raised the macro-averaged AUC of the primary model from 0.742 to 0.770. Only 20 participants met a 3.5-to-4.5-year criterion, too few for evaluation (**Supplementary Table 17**).

Applying, without re-optimization, the Youden threshold of 0.26 selected for the same model on the internal testing set, the parsimonious model identified 39 of the 55 external rapid progressors (sensitivity 0.709, against 0.640 internally) while flagging 35.4% of the cohort, with a specificity of 0.729, a positive predictive value of 0.379, a negative predictive value of 0.915 and a number needed to screen of 2.6. At the internal high-sensitivity threshold of 0.15, external sensitivity reached 0.927 with 68.7% of the cohort flagged and a positive predictive value of 0.255 (**Supplementary Table 10**).

### 3.11 Sensitivity analysis

Macro-averaged AUC was 0.786 in the main analysis and 0.784 under multiple imputation (**Supplementary Table 19**; **Supplementary Figure 10**). The complete-case analysis yielded 0.670, with 440 of 2,971 participants (14.8%) having no missing predictor. Collapsing the outcome to three classes gave 0.783 averaged over three rather than four classes, and an alternative definition of rapid progression gave 0.770 with 8.3% of participants changing class.

Eight strategies for the class imbalance were compared with the XGBoost configuration held fixed (**Supplementary Table 20**; **Supplementary Figure 11**). Macro-averaged AUC ranged from 0.745 to 0.791. Making no adjustment gave the highest macro-averaged AUC while assigning no participant to the Improvement class, giving a sensitivity and F1 of zero for that class. Inverse-frequency weighting gave 0.785 while recovering the Improvement class. SMOTE reached 0.777, ADASYN 0.778 and SMOTEENN 0.757, and random undersampling and the balanced random forest also fell below weighting.

Learning curves indicated convergence at the available training-set size: the cross-validated macro-averaged AUC rose by only 0.011 to 0.033 between half and the full training set. Apparent optimism differed sharply between model families, from 0.014 for Multinomial LR to 0.140 for the support vector machine, 0.157 for GBM, 0.174 for XGBoost and 0.227 for the random forest; the stacking ensemble was not refitted at every subsample size (**Supplementary Table 21**; **Supplementary Figure 12**).

## 4 Discussion

Our study developed and externally validated a four-class framework for CKM trajectory prediction. XGBoost attained the highest macro-averaged AUC and the stacking ensemble the highest balanced accuracy, but the confidence intervals of the six models overlapped substantially and no algorithm can be identified as superior. SHAP analysis identified baseline CKM stage, age, and metabolic indicators as consensus predictors across trajectories, and showed contributions of self-rated health, depressive symptoms, and sleep duration that were concentrated in the Rapid Progression class. The parsimonious model based on six consensus features retained more than 98% of the discriminative performance of the full model despite a substantial reduction in the number of variables. External validation in an independent hospital-based cohort was consistent with provisional transportability of the parsimonious model, subject to the shorter and more variable follow-up interval in that cohort.

Discrimination and classification accuracy are not equivalent here. Macro-averaged AUC describes ranking within a class, not correct assignment: overall accuracy was 0.463 to 0.627 and balanced accuracy 0.336 to 0.529. The models are therefore intended for screening rapid progression, where the negative predictive value reached 0.949, rather than for assigning individuals to one of four trajectories. Performance is also bounded by baseline stage: a stage-only benchmark reached a macro-averaged AUC of 0.689, the remaining 59 predictors added between −0.032 and 0.012, and within-stage models approached chance at the two lowest stages. These models therefore refine a prognosis largely set by baseline stage rather than identify independent determinants of trajectory. In contrast to previous studies that reduced CKM stage changes to a binary outcome (29, 30), this study adopted a four-class trajectory framework that simultaneously distinguishes improvement, stability, mild progression, and rapid progression. This design carries important clinical implications because different trajectories call for distinct intervention strategies. Individuals at risk of rapid progression require intensified pharmacotherapy and multidisciplinary management, whereas those with mild progression may benefit more from lifestyle interventions and routine monitoring (1, 2). Nevertheless, multiclass prediction poses challenges related to imbalanced class distributions and overlapping features between adjacent trajectories, which collectively increase discrimination difficulty compared with binary classification tasks (10, 11). Among the candidate algorithms the boosted-tree models attained the highest macro-averaged AUC, consistent with the established advantages of gradient boosting approaches in previous cardiovascular prediction research (8, 9), although the differences between algorithms lay within their confidence intervals. By iteratively fitting residuals to construct ensemble learners, gradient boosting is particularly well suited to capturing high-order interactions among predictors in the context of multisystem comorbidity characteristic of CKM syndrome (17).

Baseline CKM stage emerged as the strongest predictor for all four trajectories, contributing positively to Improvement and Stable at higher stages and positively to both progression classes at lower stages. Part of this pattern is structural rather than clinical, because improvement is unattainable from stage 0 and rapid progression from stage 3, so the direction of the contribution partly reflects which classes are attainable at each stage. A simple regression-to-the-mean explanation is insufficient to account for this pattern (31). In real-world clinical practice, individuals at advanced stages often experience escalating symptom burden that triggers more aggressive clinical responses, including intensified drug titration, more systematic dietary and exercise prescriptions, and more frequent specialist follow-up (32, 33). The improvement observed among those with higher baseline stages may therefore reflect the practical effectiveness of stratified interventions in high-risk populations. Conversely, individuals at lower baseline stages have the greatest scope for deterioration and, in the absence of triggers for intensified management, contributed most to the predicted probability of progression. This bidirectional pattern is consistent with the bidirectional CKM stage transitions previously observed in the CHARLS cohort (6) and provides longitudinal evidence from a predictive modeling perspective in support of the stage-based differentiated intervention strategy advocated by the AHA (1).

Core predictors shared across trajectories were concentrated in the metabolic and vascular domains, reflecting the fundamental role of these factors in CKM syndrome. Among these, the stable predictive value of the TyG-BMI index merits particular attention. Previous research has reported positive associations between the TyG-BMI index and cardiovascular disease, chronic kidney disease, and metabolic syndrome, with insulin resistance serving as the central hub linking glycolipid metabolic disorders, glomerular hyperfiltration, and atherosclerosis (34). The shared contributions of age and systolic blood pressure further underscore the indispensable basal role of vascular aging and hemodynamic burden in multisystem CKM injury. These findings reinforce the conceptual framework of CKM syndrome as a multisystem interactive pathological entity (1) and provide biological plausibility for the variable selection underlying the parsimonious model.

Notably, psychosocial factors, including depressive symptoms and sleep duration, showed specifically elevated importance in the Rapid Progression trajectory while contributing minimally to other trajectories. Such specificity has rarely been reported in previous studies of CKM stage evolution. Longitudinal cardiovascular research has consistently demonstrated a significant positive association between depressive symptoms and the incidence of cardiovascular events (35). This study extends that evidence by using SHAP analysis to describe the pattern of feature contributions associated with the model’s predictions of multisystem CKM deterioration. SHAP values quantify the contribution of each feature to the model output and do not establish a causal or mechanistic role. From a pathophysiological perspective, this specific association may stem from profound disruption of the neuroendocrine system by psychosocial stress. Depression and sleep deprivation can induce sustained dysregulation of the HPA axis and excessive sympathetic activation, leading to abnormal secretion of stress hormones such as cortisol, which directly exacerbates insulin resistance, endothelial injury, and systemic inflammation (36, 37). Moreover, a prospective cohort study confirmed a significant 48% increase in coronary heart disease risk associated with short sleep duration, providing high-level evidence for the link between sleep disturbances and cardiovascular outcomes (38). This cascade constitutes a core pathological basis for the transition from early metabolic dysregulation to irreversible target-organ damage in CKM syndrome. In addition, depressed individuals often exhibit reduced treatment adherence and unhealthy lifestyle habits. Previous meta-analyses and systematic reviews have confirmed that depression substantially increases the risk of medication non-adherence in cardiovascular patients through motivational deficits, social withdrawal, and pessimistic expectations regarding treatment benefits (39, 40). The compounding effect of physiological and behavioral factors offers one hypothesis for the concentration of psychosocial contributions in the Rapid Progression class.

Smoking exposure, in contrast, contributed specifically to the Mild Progression trajectory. Smoking induces atherosclerosis through reactive oxygen species accumulation, upregulation of adhesion molecule expression, and inhibition of nitric oxide bioavailability (41). Such vascular damage requires sustained exposure to propel the progression of vascular pathology from the subclinical to the clinical stage. In contrast to the acute multisystem impact exerted by psychosocial factors via HPA axis dysregulation in the Rapid Progression trajectory, the chronic endothelial toxicity of smoking is compatible with gradual CKM stage progression across adjacent stages, offering a plausible pathophysiological interpretation of this pattern.

The parsimonious model built on shared core features retained discriminative performance nearly equivalent to that of the full model while achieving a substantial reduction in the number of variables. All included indicators can be obtained through routine physical examinations and standard laboratory tests, without the need for imaging or specialized assessments. This characteristic is well suited to primary care and community health management settings, enabling preliminary stratification of CKM trajectories under resource-constrained conditions. From a public health perspective, rapid risk stratification based on a limited set of easily accessible indicators can help direct scarce medical resources toward high-risk individuals, representing a concrete implementation of the early screening and stratified management strategy for CKM syndrome advocated by the AHA (1). Five of the six parsimonious predictors were directly obtainable from routine hospital records, and the frozen models retained moderate discrimination without retraining, the random forest showing the smallest internal-to-external drop of 0.010, which supports provisional rather than established transportability across settings.

To the best of our knowledge, existing studies on CKM syndrome prediction have largely been limited to cross-sectional static risk assessments and have predominantly employed binary classification frameworks. Leveraging a nationally representative longitudinal cohort, this study is the first to apply a multiclass machine learning framework to the prediction of CKM progression trajectories, enabling differential identification of four dynamic evolution patterns and providing an effective tool for early and precise stratification of high-risk populations. Furthermore, the parsimonious model was externally validated in an independent clinical cohort, retaining moderate discrimination with six routine parameters and no retraining, although its transportability remains provisional. Several limitations should nevertheless be acknowledged. First, although external validation was performed in an independent hospital-based cohort, the relatively small sample size may have reduced the precision of performance estimates. Further validation in larger, geographically and ethnically diverse populations remains warranted. Second, chronic conditions used in the definition of CKM staging were based on participant self-report, introducing potential recall bias. Moreover, because urinary albumin-to-creatinine ratio data were not collected, early chronic kidney disease was assessed solely based on eGFR, which may have underestimated the true prevalence of CKD (5, 6). Third, the distribution of the four trajectory classes was markedly imbalanced, and although inverse-frequency weighting was employed to mitigate this issue, minority-class recognition remained limited. Future research may explore more advanced strategies such as cost-sensitive learning or stratified sampling. Fourth, the longitudinal analysis was based on only two waves of survey data, and the four-year follow-up interval may have missed interim fluctuations in CKM stage. Fifth, the external follow-up interval was shorter and more variable than the fixed four-year development interval, with a median of 1.96 years and no patient reaching four years, so a stage change observed externally is not clinically equivalent to one observed over four years, and the frozen recalibration functions did not transport. Sixth, apparent optimism was substantially larger for the tree ensembles than for the penalized multinomial model, and the learning curves indicate that little further gain is to be expected from a larger training set. Finally, the exclusion of participants at baseline stage 4 should not be read as evidence that stage 4 is biologically irreversible, a question these data cannot address. The resulting models do not apply to that highest-risk group, which is a limitation of deployment rather than a biological finding.

## 5 Conclusion

This study extended CKM stage change from a binary outcome to a four-class trajectory framework encompassing improvement, stability, mild progression, and rapid progression, and developed a multiclass trajectory prediction framework incorporating six algorithms. XGBoost attained the highest macro-averaged AUC and was used as the primary model, with the six algorithms performing comparably and no single algorithm identifiable as superior. The framework is intended for screening rapid progression rather than for assigning individuals to one of four trajectories. Baseline CKM stage, age, fasting glucose, TyG-BMI index, systolic blood pressure and triglycerides were the six consensus predictors across trajectories, with baseline stage accounting for most of the attainable discrimination and the contributions of self-rated health, depressive symptoms and sleep duration concentrated in the Rapid Progression class. Built on these six routinely available measurements, the parsimonious model preserved discrimination while substantially reducing the number of predictors, and its performance in an independent clinical cohort supports provisional transportability, pending confirmation in larger and more diverse settings.

## Data Availability

The CHARLS data analyzed in this study are publicly available at https://charls.pku.edu.cn/. The external validation data are de-identified administrative health records held by the Xining Municipal Center for Disease Control and Prevention and are not publicly available. They can be obtained from the corresponding author with that Center's permission.
