# Supplementary material for: Interpretable machine learning for multiclass trajectory prediction in cardiovascular-kidney-metabolic syndrome stage: development and external validation
Source: Front Endocrinol (Lausanne). 2026 Sep 4;17:1907821. doi: 10.3389/fendo.2026.1907821 (PMC13585525; doi:10.3389/fendo.2026.1907821)
Supplement: Supplementary file 1 [file Supplementaryfile1.docx]

Supplementary Material

# 1 Supplementary Tables

**Table S1. Variable dictionary, measurement type and missingness in the analytic cohort (N = 2,971).**

| Variable | Type | N | Available | Missing (%) | Statistic |
| --- | --- | --- | --- | --- | --- |
| Arthritis | Binary | 2971 | 2969 | 0.1 | 942 (31.7%) |
| Asthma | Binary | 2971 | 2959 | 0.4 | 105 (3.5%) |
| Cancer | Binary | 2971 | 2965 | 0.2 | 19 (0.6%) |
| Current alcohol use | Binary | 2971 | 2971 | 0 | 1021 (34.4%) |
| Current smoking | Binary | 2971 | 2966 | 0.2 | 838 (28.3%) |
| Digestive disease | Binary | 2971 | 2969 | 0.1 | 584 (19.7%) |
| Ever consumed alcohol | Binary | 2971 | 2967 | 0.1 | 1160 (39.1%) |
| Ever smoked | Binary | 2971 | 2971 | 0 | 1079 (36.3%) |
| Height (m) | Continuous | 2971 | 2971 | 0 | 1.60 ± 0.10 |
| Household registration (Hukou) | Binary | 2971 | 2969 | 0.1 | 2501 (84.2%) |
| Liver disease | Binary | 2971 | 2964 | 0.2 | 81 (2.7%) |
| Lung disease | Binary | 2971 | 2968 | 0.1 | 224 (7.5%) |
| Marital status | Binary | 2971 | 2971 | 0 | 2718 (91.5%) |
| Memory-related disease | Binary | 2971 | 2966 | 0.2 | 24 (0.8%) |
| Psychiatric disorder | Binary | 2971 | 2962 | 0.3 | 27 (0.9%) |
| Residence type | Binary | 2971 | 2971 | 0 | 1955 (65.8%) |
| Retirement status | Binary | 2971 | 2920 | 1.7 | 245 (8.4%) |
| Serum creatinine (mg/dL) | Continuous | 2971 | 2971 | 0 | 1.00 ± 0.05 |
| Sex | Binary | 2971 | 2971 | 0 | 1346 (45.3%) |
| Age (years) | Continuous | 2971 | 2971 | 0 | 57.16 ± 7.84 |
| Blood urea nitrogen (mg/dL) | Continuous | 2971 | 2971 | 0 | 15.42 ± 4.14 |
| Body mass index (kg/m^2^) | Continuous | 2971 | 2971 | 0 | 23.97 ± 3.16 |
| Body weight (kg) | Continuous | 2971 | 2971 | 0 | 60.29 ± 9.92 |
| C-reactive protein (mg/L) | Continuous | 2971 | 2971 | 0 | 2.41 ± 6.62 |
| CES-D-10, depressive symptoms score | Continuous | 2971 | 2952 | 0.6 | 7.94 ± 6.15 |
| Diastolic blood pressure (mmHg) | Continuous | 2971 | 2970 | 0 | 74.92 ± 11.30 |
| Episodic memory score (0-10) | Continuous | 2971 | 2709 | 8.8 | 3.94 ± 1.66 |
| Executive function score (0-11) | Continuous | 2971 | 2643 | 11 | 8.33 ± 2.47 |
| Fasting plasma glucose (mg/dL) | Continuous | 2971 | 2971 | 0 | 108.71 ± 33.99 |
| Global cognitive score (0-21) | Continuous | 2971 | 2473 | 16.8 | 12.46 ± 3.34 |
| HDL cholesterol (mg/dL) | Continuous | 2971 | 2971 | 0 | 50.86 ± 13.85 |
| Heart rate (bpm) | Continuous | 2971 | 2970 | 0 | 72.25 ± 10.07 |
| Hematocrit (%) | Continuous | 2971 | 2969 | 0.1 | 41.81 ± 6.25 |
| Hemoglobin (g/dL) | Continuous | 2971 | 2919 | 1.8 | 14.59 ± 2.23 |
| LDL cholesterol (mg/dL) | Continuous | 2971 | 2968 | 0.1 | 120.29 ± 32.26 |
| Left handgrip strength (kg) | Continuous | 2971 | 2927 | 1.5 | 31.53 ± 10.24 |
| Mean corpuscular volume (fL) | Continuous | 2971 | 2919 | 1.8 | 90.58 ± 8.06 |
| Peak expiratory flow (L/min) | Continuous | 2971 | 2882 | 3 | 307.60 ± 123.75 |
| Platelet count (x10^9^/L) | Continuous | 2971 | 2918 | 1.8 | 213.22 ± 71.94 |
| Right handgrip strength (kg) | Continuous | 2971 | 2922 | 1.6 | 32.89 ± 10.58 |
| Sleep duration (hours/night) | Continuous | 2971 | 2940 | 1 | 6.45 ± 1.81 |
| Systolic blood pressure (mmHg) | Continuous | 2971 | 2971 | 0 | 126.61 ± 17.82 |
| Total cholesterol (mg/dL) | Continuous | 2971 | 2971 | 0 | 196.58 ± 34.68 |
| Triglycerides (mg/dL) | Continuous | 2971 | 2971 | 0 | 129.99 ± 95.17 |
| TyG-BMI index | Continuous | 2971 | 2971 | 0 | 208.36 ± 35.25 |
| Waist circumference (cm) | Continuous | 2971 | 2971 | 0 | 85.44 ± 11.07 |
| White blood cell count (x10^3^/uL) | Continuous | 2971 | 2915 | 1.9 | 6.26 ± 1.88 |
| ADL disability count | Ordinal | 2971 | 2944 | 0.9 | 0.21 ± 0.66 |
| Baseline CKM stage (Wave 1) | Ordinal | 2971 | 2971 | 0 | 1.67 ± 0.80 |
| Education level | Ordinal | 2971 | 2970 | 0 | 1.98 ± 1.04 |
| Glycated hemoglobin HbA1c (%) | Continuous | 2971 | 2971 | 0 | 5.30 ± 0.85 |
| IADL disability count | Ordinal | 2971 | 2971 | 0 | 0.30 ± 0.79 |
| Life satisfaction | Ordinal | 2971 | 2750 | 7.4 | 3.09 ± 0.69 |
| Self-rated health | Ordinal | 2971 | 2970 | 0 | 3.08 ± 0.86 |
| Triglyceride-glucose index (TyG) | Continuous | 2971 | 2971 | 0 | 8.67 ± 0.71 |
| Uric acid (mg/dL) | Continuous | 2971 | 2971 | 0 | 4.39 ± 1.22 |
| Cystatin C (mg/L) | Continuous | 2971 | 2971 | 0 | 1.02 ± 0.25 |
| Physical exercise | Binary | 2971 | 2958 | 0.4 | 1139 (38.5%) |
| Total household income (CNY/year) | Continuous | 2971 | 2383 | 19.7 | 23502.98 ± 21847.63 |
| Total physical activity (MET-min/week) | Continuous | 2971 | 2617 | 11.9 | 3127.40 ± 3842.55 |

**Notes:** Continuous variables are presented as mean ± standard deviation. Binary variables are presented as n (%). Ordinal variables are presented as mean ± standard deviation. All values are from the pre-imputation analytic sample. Missing % indicates the proportion of missing observations for each variable.

**Table S2. Software environment, package versions and random seeds.**

| **Item** | **Value** |
| --- | --- |
| R version | R version 4.4.0 (2024-04-24 ucrt) |
| Platform | x86_64-w64-mingw32 |
| Operating system | Windows 11 x64 (build 26200) |
| Random seed | 2026 |
| Parallel threads | 1 (single-threaded, for exact reproducibility) |

**Notes:** The support vector machine was fitted with e1071 rather than kernlab. The kernlab implementation cannot combine per-class weights with multiclass probability estimates, which is why the submitted analysis fitted the support vector machine without class weights. The radial kernel is parameterised identically in the two packages and the bandwidth grid is still seeded by the kernlab heuristic, so the search space is unchanged.

**Table S3. Stacking ensemble architecture and inverse-frequency class weights.**

| **Component** | **Specification** | **Value** |
| --- | --- | --- |
| Base Model (Level 1) | RF | 4-class probabilities; 60 input features |
| Base Model (Level 1) | XGBoost | 4-class probabilities; 60 input features |
| Base Model (Level 1) | GBM | 4-class probabilities; 60 input features |
| Meta-Learner (Level 2) | Multinomial LR (glmnet) | 4-class final prediction; 12 input features |
| Inverse-frequency class weight | Improvement | n = 188 in training set; weight 3.164 |
| Inverse-frequency class weight | Stable | n = 1320 in training set; weight 0.451 |
| Inverse-frequency class weight | Mild Progression | n = 524 in training set; weight 1.135 |
| Inverse-frequency class weight | Rapid Progression | n = 347 in training set; weight 1.714 |

**Table S4. Hyper-parameter search space and selected values.**

| **Model** | **N combinations** | **Hyperparameter** | **Values searched** |
| --- | --- | --- | --- |
| Multinomial LR | 30 | alpha | 0, 0.25, 0.5, 0.75, 1 |
| Multinomial LR | 30 | lambda | 0.001, 0.005, 0.01, 0.025, 0.05, 0.1 |
| RF | 12 | num.trees | 1000 |
| RF | 12 | mtry | 4, 8, 12, 15 |
| RF | 12 | min.node.size | 5, 10, 20 |
| XGBoost | 24 | nrounds | 200, 400 |
| XGBoost | 24 | eta | 0.03, 0.1 |
| XGBoost | 24 | max_depth | 3, 4, 6 |
| XGBoost | 24 | subsample | 0.8 |
| XGBoost | 24 | colsample | 0.8 |
| XGBoost | 24 | min_child_weight | 1, 5 |
| GBM | 24 | n.trees | 300, 600 |
| GBM | 24 | interaction.depth | 2, 3, 4 |
| GBM | 24 | shrinkage | 0.03, 0.1 |
| GBM | 24 | n.minobsinnode | 10, 20 |
| SVM | 12 | sigma | 0.00643438244134176, 0.0128687648826835, 0.025737529765367 |
| SVM | 12 | C | 0.5, 1, 2, 4 |

**Notes:** Tuning used grid search with five-fold stratified cross-validation on the training set only, with the fold assignment shared across all algorithms, selecting on macro-averaged one-versus-rest AUC. The selected configuration was then refitted on the whole training set. The test set was used once, for final evaluation.

**Table S5. CKM stage transition matrix from Wave 1 (2011) to Wave 3 (2015).**

| Wave 1/Wave 3 | Stage 0 | Stage 1 | Stage 2 | Stage 3 | Stage 4 |
| --- | --- | --- | --- | --- | --- |
| Stage 0 | 41 | 134 | 143 | 26 | 14 |
| Stage 1 | 22 | 191 | 246 | 37 | 28 |
| Stage 2 | 21 | 151 | 1225 | 234 | 185 |
| Stage 3 | 1 | 3 | 37 | 192 | 40 |
| Stage 4 | 0 | 0 | 0 | 0 | 471 |

**Notes:** Cell values represent the number of participants transitioning from the Wave 1 (row) CKM stage to the Wave 3 (column) CKM stage over the 4-year follow-up period (2011–2015). The full sample includes all participants with valid CKM staging at both waves, including those with baseline CKM stage 4. Stage 4 participants (n = 471) showed no stage regression (100% remained at stage 4) and were excluded from the analytic sample.

**Table S6. Reachable and observed outcome classes by baseline CKM stage.**

| **Baseline stage** | **N in stratum** | **Trajectory class** | **N observed** | **% of stratum** | **Structurally reachable** |
| --- | --- | --- | --- | --- | --- |
| 0 | 358 | Improvement | 0 | 0.0 | FALSE |
|  |  | Stable | 41 | 11.5 | TRUE |
|  |  | Mild Progression | 134 | 37.4 | TRUE |
|  |  | Rapid Progression | 183 | 51.1 | TRUE |
| 1 | 524 | Improvement | 22 | 4.2 | TRUE |
|  |  | Stable | 191 | 36.5 | TRUE |
|  |  | Mild Progression | 246 | 46.9 | TRUE |
|  |  | Rapid Progression | 65 | 12.4 | TRUE |
| 2 | 1816 | Improvement | 172 | 9.5 | TRUE |
|  |  | Stable | 1225 | 67.5 | TRUE |
|  |  | Mild Progression | 234 | 12.9 | TRUE |
|  |  | Rapid Progression | 185 | 10.2 | TRUE |
| 3 | 273 | Improvement | 41 | 15.0 | TRUE |
|  |  | Stable | 192 | 70.3 | TRUE |
|  |  | Mild Progression | 40 | 14.7 | TRUE |
|  |  | Rapid Progression | 0 | 0.0 | FALSE |

**Notes:** A class is structurally reachable from a baseline stage if the corresponding change in stage is arithmetically possible from that starting point. Participants at baseline stage 4 were excluded from the analytic cohort because no progression category is reachable from the terminal stage, so their outcome is determined by definition rather than predicted.

**Table S7. Baseline characteristics stratified by CKM trajectory class (N = 2,971).**

| **Characteristic** | **Overall (N = 2,971)** | **Improvement (n = 235)** | **Stable (n = 1,649)** | **Mild progression (n = 654)** | **Rapid progression (n = 433)** | **P-value** |
| --- | --- | --- | --- | --- | --- | --- |
| Sex, male, n (%) | 1,346 (45.3%) | 120 (51.1%) | 696 (42.2%) | 329 (50.3%) | 201 (46.4%) | <0.001 |
| Marital status, married or partnered, n (%) | 2,718 (91.5%) | 220 (93.6%) | 1,510 (91.6%) | 595 (91.0%) | 393 (90.8%) | 0.595 |
| Residence type, rural residence, n (%) | 1,955 (65.8%) | 146 (62.1%) | 1,047 (63.5%) | 458 (70.0%) | 304 (70.2%) | 0.003 |
| Household registration (Hukou), agricultural household registration, n (%) | 2,501 (84.2%) | 202 (86.0%) | 1,389 (84.3%) | 555 (85.0%) | 355 (82.0%) | 0.479 |
| **Self-rated health, n (%)** |  |  |  |  |  | <0.001 |
| 1 = very good | 80 (2.7%) | 4 (1.7%) | 36 (2.2%) | 23 (3.5%) | 17 (3.9%) |  |
| 2 = good | 570 (19.2%) | 40 (17.0%) | 299 (18.1%) | 122 (18.7%) | 109 (25.2%) |  |
| 3 = fair | 1,556 (52.4%) | 112 (47.7%) | 886 (53.8%) | 350 (53.5%) | 208 (48.0%) |  |
| 4 = poor | 575 (19.4%) | 61 (26.0%) | 317 (19.2%) | 130 (19.9%) | 67 (15.5%) |  |
| 5 = very poor | 189 (6.4%) | 18 (7.7%) | 110 (6.7%) | 29 (4.4%) | 32 (7.4%) |  |
| ADL disability count, median (IQR); mean ± SD | 0.00 (0.00, 0.00); 0.21 ± 0.66 | 0.00 (0.00, 0.00); 0.08 ± 0.38 | 0.00 (0.00, 0.00); 0.22 ± 0.67 | 0.00 (0.00, 0.00); 0.21 ± 0.65 | 0.00 (0.00, 0.00); 0.23 ± 0.76 | 0.018 |
| Cancer, yes, n (%) | 19 (0.6%) | 0 (0.0%) | 10 (0.6%) | 6 (0.9%) | 3 (0.7%) | 0.580 |
| Lung disease, yes, n (%) | 224 (7.5%) | 17 (7.2%) | 114 (6.9%) | 52 (8.0%) | 41 (9.5%) | 0.330 |
| Psychiatric disorder, yes, n (%) | 27 (0.9%) | 0 (0.0%) | 18 (1.1%) | 3 (0.5%) | 6 (1.4%) | 0.144 |
| Arthritis, yes, n (%) | 942 (31.7%) | 78 (33.2%) | 526 (31.9%) | 205 (31.3%) | 133 (30.9%) | 0.918 |
| Liver disease, yes, n (%) | 81 (2.7%) | 8 (3.4%) | 37 (2.2%) | 27 (4.1%) | 9 (2.1%) | 0.060 |
| Digestive disease, yes, n (%) | 584 (19.7%) | 49 (20.9%) | 300 (18.2%) | 139 (21.3%) | 96 (22.2%) | 0.155 |
| Asthma, yes, n (%) | 105 (3.5%) | 4 (1.7%) | 56 (3.4%) | 23 (3.5%) | 22 (5.1%) | 0.142 |
| Memory-related disease, yes, n (%) | 24 (0.8%) | 1 (0.4%) | 7 (0.4%) | 10 (1.5%) | 6 (1.4%) | 0.019 |
| Ever consumed alcohol, ever drank alcohol, n (%) | 1,160 (39.1%) | 103 (43.8%) | 615 (37.4%) | 272 (41.7%) | 170 (39.3%) | 0.104 |
| Current alcohol use, drank in the past year, n (%) | 1,021 (34.4%) | 96 (40.9%) | 554 (33.6%) | 231 (35.3%) | 140 (32.3%) | 0.118 |
| Ever smoked, ever smoked, n (%) | 1,079 (36.3%) | 88 (37.4%) | 563 (34.1%) | 275 (42.0%) | 153 (35.3%) | 0.005 |
| Current smoking, currently smoking, n (%) | 838 (28.3%) | 68 (29.1%) | 440 (26.7%) | 217 (33.2%) | 113 (26.1%) | 0.012 |
| Total household income (CNY/year), median (IQR) | 12,500.00 (7,645.00, 19,600.00) | 12,870.00 (6,550.00, 13,850.00) | 12,730.00 (8,800.00, 19,000.00) | 12,080.00 (6,325.00, 22,300.00) | 12,060.00 (7,000.00, 21,600.00) | 0.445 |
| Retirement status, retired, n (%) | 245 (8.4%) | 11 (4.8%) | 131 (8.1%) | 57 (8.9%) | 46 (10.8%) | 0.056 |
| Systolic blood pressure (mmHg), median (IQR) | 124.00 (113.00, 138.00) | 127.00 (114.00, 138.00) | 127.00 (115.00, 142.00) | 120.00 (110.00, 132.00) | 121.00 (111.00, 132.00) | <0.001 |
| Diastolic blood pressure (mmHg), median (IQR) | 74.00 (67.00, 82.00) | 76.00 (68.00, 84.00) | 76.00 (69.00, 84.00) | 71.00 (65.00, 78.00) | 72.00 (65.00, 78.00) | <0.001 |
| Heart rate (bpm), median (IQR) | 72.00 (65.00, 78.00) | 71.00 (65.00, 77.00) | 72.00 (66.00, 79.00) | 71.00 (65.00, 78.00) | 71.00 (64.00, 77.00) | <0.001 |
| Left handgrip strength (kg), median (IQR) | 30.00 (25.00, 38.00) | 32.00 (26.00, 40.00) | 30.00 (25.00, 38.00) | 31.00 (25.00, 38.00) | 30.00 (24.00, 37.00) | 0.010 |
| Right handgrip strength (kg), median (IQR) | 32.00 (26.00, 40.00) | 34.00 (28.00, 42.00) | 32.00 (26.00, 40.00) | 32.00 (27.00, 40.00) | 32.00 (25.00, 38.00) | 0.005 |
| Height (m), median (IQR) | 1.60 (1.53, 1.67) | 1.61 (1.54, 1.67) | 1.60 (1.53, 1.66) | 1.60 (1.54, 1.67) | 1.60 (1.53, 1.67) | 0.173 |
| Body weight (kg), median (IQR) | 59.00 (53.00, 66.00) | 58.00 (53.00, 64.00) | 60.00 (54.00, 67.00) | 59.00 (53.00, 65.00) | 57.00 (52.00, 65.00) | <0.001 |
| Waist circumference (cm), median (IQR) | 86.00 (79.00, 92.00) | 83.00 (77.00, 88.00) | 87.00 (81.00, 93.00) | 85.00 (79.00, 92.00) | 83.00 (78.00, 91.00) | <0.001 |
| Body mass index (kg/m2), median (IQR) | 24.00 (22.00, 26.00) | 23.00 (21.00, 25.00) | 24.00 (22.00, 26.00) | 23.00 (21.25, 26.00) | 23.00 (21.00, 25.00) | <0.001 |
| Peak expiratory flow (L/min), median (IQR) | 300.00 (220.00, 380.00) | 310.00 (240.00, 400.00) | 300.00 (220.00, 380.00) | 310.00 (220.00, 390.00) | 280.00 (200.00, 370.00) | 0.008 |
| CES-D-10 depressive symptoms score, median (IQR) | 7.00 (3.00, 11.00) | 6.00 (3.00, 11.00) | 6.00 (3.00, 11.00) | 7.00 (3.00, 11.00) | 7.00 (3.00, 13.00) | 0.032 |
| **Life satisfaction, n (%)** |  |  |  |  |  | 0.935 |
| 1 = completely satisfied | 54 (2.0%) | 5 (2.3%) | 30 (2.0%) | 14 (2.3%) | 5 (1.2%) |  |
| 2 = very satisfied | 335 (12.2%) | 24 (11.1%) | 179 (11.7%) | 73 (12.1%) | 59 (14.7%) |  |
| 3 = somewhat satisfied | 1,737 (63.2%) | 139 (64.1%) | 974 (63.8%) | 380 (62.8%) | 244 (60.8%) |  |
| 4 = not very satisfied | 571 (20.8%) | 46 (21.2%) | 316 (20.7%) | 126 (20.8%) | 83 (20.7%) |  |
| 5 = not at all satisfied | 53 (1.9%) | 3 (1.4%) | 28 (1.8%) | 12 (2.0%) | 10 (2.5%) |  |
| Sleep duration (hours/night), median (IQR) | 7.00 (5.00, 8.00) | 7.00 (6.00, 8.00) | 7.00 (6.00, 8.00) | 7.00 (5.00, 8.00) | 6.00 (5.00, 8.00) | 0.016 |
| White blood cell count (x10^3/uL), median (IQR) | 6.00 (4.92, 7.31) | 6.00 (4.92, 7.32) | 5.98 (4.91, 7.29) | 6.05 (4.96, 7.37) | 5.97 (4.90, 7.28) | 0.408 |
| Mean corpuscular volume (fL), median (IQR) | 91.00 (87.00, 95.00) | 91.00 (87.00, 95.50) | 91.00 (87.00, 95.00) | 91.00 (87.00, 95.00) | 91.00 (87.00, 96.00) | 0.215 |
| Platelet count (x10^9/L), median (IQR) | 207.00 (165.00, 255.00) | 210.00 (168.00, 251.00) | 207.00 (164.00, 259.00) | 205.00 (165.00, 249.00) | 210.00 (166.00, 258.00) | 0.411 |
| Blood urea nitrogen (mg/dL), median (IQR) | 15.00 (12.00, 18.00) | 15.00 (12.00, 17.00) | 15.00 (12.00, 18.00) | 15.00 (13.00, 18.00) | 15.00 (12.00, 17.00) | 0.544 |
| Fasting plasma glucose (mg/dL), median (IQR) | 102.00 (95.00, 112.00) | 105.00 (98.00, 122.50) | 104.00 (96.00, 113.00) | 100.50 (94.00, 110.00) | 96.00 (91.00, 104.00) | <0.001 |
| Serum creatinine (mg/dL), median (IQR) | 0.77 (0.64, 0.93) | 0.78 (0.65, 0.94) | 0.77 (0.64, 0.92) | 0.78 (0.65, 0.94) | 0.77 (0.64, 0.93) | 0.651 |
| Total cholesterol (mg/dL), median (IQR) | 193.00 (171.00, 217.00) | 190.00 (168.00, 216.00) | 195.00 (173.00, 218.00) | 193.00 (170.00, 217.00) | 191.00 (167.00, 213.00) | 0.024 |
| Triglycerides (mg/dL), median (IQR) | 104.00 (74.50, 151.50) | 124.00 (76.50, 171.50) | 115.00 (80.00, 171.00) | 90.00 (70.00, 122.00) | 95.00 (71.00, 126.00) | <0.001 |
| HDL cholesterol (mg/dL), median (IQR) | 49.00 (41.00, 60.00) | 48.00 (39.00, 58.00) | 48.00 (40.00, 58.00) | 51.00 (43.00, 62.00) | 52.00 (44.00, 62.00) | <0.001 |
| LDL cholesterol (mg/dL), median (IQR) | 117.00 (98.00, 140.00) | 112.00 (92.00, 132.00) | 118.00 (98.00, 141.00) | 118.00 (101.00, 143.00) | 117.00 (98.00, 136.00) | 0.006 |
| C-reactive protein (mg/L), median (IQR) | 0.82 (0.31, 2.21) | 0.77 (0.29, 2.08) | 0.82 (0.31, 2.21) | 0.77 (0.29, 2.08) | 0.77 (0.29, 2.08) | 0.132 |
| Glycated hemoglobin HbA1c (%), median (IQR) | 5.23 (4.70, 5.83) | 5.34 (4.80, 5.93) | 5.30 (4.77, 5.90) | 5.18 (4.65, 5.77) | 5.02 (4.49, 5.62) | <0.001 |
| Uric acid (mg/dL), median (IQR) | 4.23 (3.52, 5.08) | 4.29 (3.57, 5.15) | 4.20 (3.49, 5.05) | 4.28 (3.56, 5.14) | 4.24 (3.53, 5.10) | 0.464 |
| Hematocrit (%), median (IQR) | 42.00 (38.00, 45.00) | 42.00 (38.00, 45.50) | 42.00 (38.00, 45.00) | 42.00 (38.00, 45.00) | 42.00 (38.00, 46.00) | 0.997 |
| Hemoglobin (g/dL), median (IQR) | 14.00 (13.00, 16.00) | 15.00 (13.00, 16.00) | 14.00 (13.00, 16.00) | 14.00 (13.00, 15.00) | 14.00 (13.00, 16.00) | 0.636 |
| Cystatin C (mg/L), median (IQR) | 1.00 (0.90, 1.11) | 0.99 (0.89, 1.10) | 0.99 (0.89, 1.10) | 1.01 (0.91, 1.12) | 1.01 (0.91, 1.12) | 0.661 |
| Age (years), median (IQR) | 57.00 (51.00, 63.00) | 55.00 (51.00, 59.00) | 56.00 (50.00, 62.00) | 59.00 (52.00, 65.00) | 59.00 (52.00, 64.00) | <0.001 |
| **Education level, n (%)** |  |  |  |  |  | 0.209 |
| 1 = illiterate or no formal schooling | 1,315 (44.3%) | 87 (37.0%) | 734 (44.5%) | 302 (46.2%) | 192 (44.3%) |  |
| 2 = primary school | 695 (23.4%) | 55 (23.4%) | 373 (22.6%) | 163 (24.9%) | 104 (24.0%) |  |
| 3 = middle school | 656 (22.1%) | 66 (28.1%) | 373 (22.6%) | 128 (19.6%) | 89 (20.6%) |  |
| 4 = high school or above | 304 (10.2%) | 27 (11.5%) | 168 (10.2%) | 61 (9.3%) | 48 (11.1%) |  |
| Physical exercise, yes, n (%) | 2,871 (96.6%) | 227 (96.6%) | 1,596 (96.8%) | 636 (97.2%) | 412 (95.2%) | 0.283 |
| Total physical activity (MET-min/week), median (IQR) | 6,530.00 (1,732.20, 13,433.83) | 6,530.00 (1,732.20, 13,433.83) | 6,570.00 (1,742.81, 13,516.12) | 6,650.00 (1,764.04, 13,680.70) | 6,260.00 (1,660.58, 12,878.37) | 0.975 |
| Episodic memory score (0-10), median (IQR) | 4.00 (3.00, 5.00) | 4.00 (3.00, 5.00) | 4.00 (3.00, 5.00) | 4.00 (3.00, 5.00) | 4.00 (3.00, 5.00) | 0.514 |
| Executive function score (0-11), median (IQR) | 9.00 (7.00, 10.00) | 9.00 (7.00, 10.00) | 9.00 (7.00, 10.00) | 9.00 (7.00, 10.00) | 9.00 (7.00, 10.00) | 0.998 |
| Global cognitive score (0-21), median (IQR) | 13.00 (11.00, 15.00) | 13.00 (11.00, 15.00) | 13.00 (11.00, 15.00) | 13.00 (11.00, 14.00) | 13.00 (11.00, 14.00) | 0.725 |
| IADL disability count, median (IQR); mean ± SD | 0.00 (0.00, 0.00); 0.30 ± 0.79 | 0.00 (0.00, 0.00); 0.20 ± 0.61 | 0.00 (0.00, 0.00); 0.29 ± 0.78 | 0.00 (0.00, 0.00); 0.32 ± 0.80 | 0.00 (0.00, 0.00); 0.37 ± 0.87 | 0.075 |
| Triglyceride-glucose index (TyG), median (IQR) | 8.58 (8.17, 9.05) | 8.78 (8.23, 9.26) | 8.70 (8.25, 9.18) | 8.42 (8.10, 8.81) | 8.43 (8.08, 8.79) | <0.001 |
| TyG-BMI index, median (IQR) | 203.00 (182.00, 230.00) | 201.00 (182.00, 221.00) | 210.00 (188.00, 237.00) | 198.00 (176.00, 221.00) | 191.00 (173.00, 217.00) | <0.001 |
| **Baseline CKM stage (Wave 1), n (%)** |  |  |  |  |  | <0.001 |
| 0 = stage 0 | 358 (12.0%) | 0 (0.0%) | 41 (2.5%) | 134 (20.5%) | 183 (42.3%) |  |
| 1 = stage 1 | 524 (17.6%) | 22 (9.4%) | 191 (11.6%) | 246 (37.6%) | 65 (15.0%) |  |
| 2 = stage 2 | 1,816 (61.1%) | 172 (73.2%) | 1,225 (74.3%) | 234 (35.8%) | 185 (42.7%) |  |
| 3 = stage 3 | 273 (9.2%) | 41 (17.4%) | 192 (11.6%) | 40 (6.1%) | 0 (0.0%) |  |

**Notes:** CKM trajectories are defined as: Improvement (decrease ≥1 CKM stage); Stable (no stage change); Mild Progression (increase of 1 CKM stage); Rapid Progression (increase ≥2 CKM stages). All values are from the analytic sample. Continuous variables are summarised as median (interquartile range) and compared with the Kruskal–Wallis test; categorical variables are summarised as n (%) and compared with the chi-square test, or Fisher's exact test with simulated P values where expected counts are small. Percentages for categorical variables are calculated over observations with the variable recorded. ADL and IADL disability counts are zero-inflated. The class-specific means and standard deviations reported here imply that no fewer than 82% of participants in every trajectory class report no ADL limitation, and no fewer than 71% report no IADL limitation, so the median and both quartiles are necessarily 0.00 in every column and the interquartile range carries no information about between-class differences. The mean and standard deviation are therefore reported alongside the median (IQR) for these two rows.

**Table S8. Class-specific discrimination, classification, confusion breakdown and pairwise comparison on the internal test set (N = 592).**

| **Model** | **Trajectory class** | **N true** | **N pred.** | **Predicted class distribution, n (%)** | **AUC** | **Sens.** | **Spec.** | **PPV** | **NPV** | **F1** | **F1 status** | **Brier, class** | **P vs XGBoost** | **Macro-AUC** | **Micro-AUC** | **Macro = mean** | **Overall acc.** | **Balanced acc.** | **Kappa** | **Macro-F1, all 4** | **Macro-F1, estimable** | **N never predicted** | **Weighted F1** | **Log loss** | **Brier, overall** |
| --- | --- | --- | --- | --- | --- | --- | --- | --- | --- | --- | --- | --- | --- | --- | --- | --- | --- | --- | --- | --- | --- | --- | --- | --- | --- |
| Multinomial LR | Improvement | 47 | 197 | 33 (70.2) / 8 (17.0) / 3 (6.4) / 3 (6.4) | 0.786 | 0.702 | 0.699 | 0.168 | 0.965 | 0.27 | Estimable | 0.107 | 0.573 |  |  |  |  |  |  |  |  |  |  |  |  |
|  | Stable | 329 | 193 | 135 (41.0) / 144 (43.8) / 29 (8.8) / 21 (6.4) | 0.75 | 0.438 | 0.814 | 0.746 | 0.536 | 0.552 | Estimable | 0.289 | 0.574 |  |  |  |  |  |  |  |  |  |  |  |  |
|  | Mild Progression | 130 | 89 | 17 (13.1) / 25 (19.2) / 49 (37.7) / 39 (30.0) | 0.733 | 0.377 | 0.913 | 0.551 | 0.839 | 0.447 | Estimable | 0.155 | 0.063 |  |  |  |  |  |  |  |  |  |  |  |  |
|  | Rapid Progression | 86 | 113 | 12 (14.0) / 16 (18.6) / 8 (9.3) / 50 (58.1) | 0.803 | 0.581 | 0.875 | 0.442 | 0.925 | 0.503 | Estimable | 0.105 | 0.457 |  |  |  |  |  |  |  |  |  |  |  |  |
|  | All classes | 592 | 592 |  | 0.768 |  |  |  |  |  |  |  |  | 0.768 | 0.749 | TRUE | 0.466 | 0.525 | 0.27 | 0.443 | 0.443 | 0 | 0.499 | 1.17 | 0.656 |
| RF | Improvement | 47 | 43 | 13 (27.7) / 28 (59.6) / 5 (10.6) / 1 (2.1) | 0.765 | 0.277 | 0.945 | 0.302 | 0.938 | 0.289 | Estimable | 0.078 | 0.011 |  |  |  |  |  |  |  |  |  |  |  |  |
|  | Stable | 329 | 350 | 25 (7.6) / 251 (76.3) / 50 (15.2) / 3 (0.9) | 0.758 | 0.763 | 0.624 | 0.717 | 0.678 | 0.739 | Estimable | 0.24 | 0.913 |  |  |  |  |  |  |  |  |  |  |  |  |
|  | Mild Progression | 130 | 147 | 3 (2.3) / 44 (33.8) / 71 (54.6) / 12 (9.2) | 0.762 | 0.546 | 0.835 | 0.483 | 0.867 | 0.513 | Estimable | 0.147 | 0.322 |  |  |  |  |  |  |  |  |  |  |  |  |
|  | Rapid Progression | 86 | 52 | 2 (2.3) / 27 (31.4) / 21 (24.4) / 36 (41.9) | 0.798 | 0.419 | 0.968 | 0.692 | 0.907 | 0.522 | Estimable | 0.1 | 0.17 |  |  |  |  |  |  |  |  |  |  |  |  |
|  | All classes | 592 | 592 |  | 0.771 |  |  |  |  |  |  |  |  | 0.771 | 0.833 | TRUE | 0.627 | 0.501 | 0.376 | 0.516 | 0.516 | 0 | 0.622 | 1.027 | 0.566 |
| XGBoost | Improvement | 47 | 110 | 25 (53.2) / 16 (34.0) / 4 (8.5) / 2 (4.3) | 0.797 | 0.532 | 0.844 | 0.227 | 0.954 | 0.318 | Estimable | 0.09 | Reference |  |  |  |  |  |  |  |  |  |  |  |  |
|  | Stable | 329 | 248 | 70 (21.3) / 186 (56.5) / 61 (18.5) / 12 (3.6) | 0.757 | 0.565 | 0.764 | 0.75 | 0.584 | 0.645 | Estimable | 0.257 | Reference |  |  |  |  |  |  |  |  |  |  |  |  |
|  | Mild Progression | 130 | 166 | 13 (10.0) / 21 (16.2) / 76 (58.5) / 20 (15.4) | 0.769 | 0.585 | 0.805 | 0.458 | 0.873 | 0.514 | Estimable | 0.146 | Reference |  |  |  |  |  |  |  |  |  |  |  |  |
|  | Rapid Progression | 86 | 68 | 2 (2.3) / 25 (29.1) / 25 (29.1) / 34 (39.5) | 0.82 | 0.395 | 0.933 | 0.5 | 0.901 | 0.442 | Estimable | 0.1 | Reference |  |  |  |  |  |  |  |  |  |  |  |  |
|  | All classes | 592 | 592 |  | 0.786 |  |  |  |  |  |  |  |  | 0.786 | 0.805 | TRUE | 0.542 | 0.519 | 0.321 | 0.48 | 0.48 | 0 | 0.56 | 1.057 | 0.593 |
| GBM | Improvement | 47 | 105 | 24 (51.1) / 17 (36.2) / 5 (10.6) / 1 (2.1) | 0.797 | 0.511 | 0.851 | 0.229 | 0.953 | 0.316 | Estimable | 0.091 | 0.954 |  |  |  |  |  |  |  |  |  |  |  |  |
|  | Stable | 329 | 253 | 68 (20.7) / 185 (56.2) / 57 (17.3) / 19 (5.8) | 0.749 | 0.562 | 0.741 | 0.731 | 0.575 | 0.636 | Estimable | 0.255 | 0.281 |  |  |  |  |  |  |  |  |  |  |  |  |
|  | Mild Progression | 130 | 159 | 10 (7.7) / 28 (21.5) / 74 (56.9) / 18 (13.8) | 0.758 | 0.569 | 0.816 | 0.465 | 0.871 | 0.512 | Estimable | 0.148 | 0.138 |  |  |  |  |  |  |  |  |  |  |  |  |
|  | Rapid Progression | 86 | 75 | 3 (3.5) / 23 (26.7) / 23 (26.7) / 37 (43.0) | 0.804 | 0.43 | 0.925 | 0.493 | 0.905 | 0.46 | Estimable | 0.102 | 0.041 |  |  |  |  |  |  |  |  |  |  |  |  |
|  | All classes | 592 | 592 |  | 0.777 |  |  |  |  |  |  |  |  | 0.777 | 0.8 | TRUE | 0.541 | 0.518 | 0.315 | 0.481 | 0.481 | 0 | 0.558 | 1.063 | 0.597 |
| SVM | Improvement | 47 | 0 | 0 (0.0) / 43 (91.5) / 4 (8.5) / 0 (0.0) | 0.735 | 0 | 1 | NA | 0.921 | 0 | Class never predicted; F1 set to 0 by pre-specified rule | 0.069 | 0.01 |  |  |  |  |  |  |  |  |  |  |  |  |
|  | Stable | 329 | 472 | 0 (0.0) / 309 (93.9) / 20 (6.1) / 0 (0.0) | 0.739 | 0.939 | 0.38 | 0.655 | 0.833 | 0.772 | Estimable | 0.197 | 0.209 |  |  |  |  |  |  |  |  |  |  |  |  |
|  | Mild Progression | 130 | 107 | 0 (0.0) / 87 (66.9) / 39 (30.0) / 4 (3.1) | 0.732 | 0.3 | 0.853 | 0.364 | 0.812 | 0.329 | Estimable | 0.157 | 0.044 |  |  |  |  |  |  |  |  |  |  |  |  |
|  | Rapid Progression | 86 | 13 | 0 (0.0) / 33 (38.4) / 44 (51.2) / 9 (10.5) | 0.792 | 0.105 | 0.992 | 0.692 | 0.867 | 0.182 | Estimable | 0.101 | 0.231 |  |  |  |  |  |  |  |  |  |  |  |  |
|  | All classes | 592 | 592 |  | 0.75 |  |  |  |  |  |  |  |  | 0.75 | 0.842 | TRUE | 0.603 | 0.336 | 0.228 | 0.321 | 0.428 | 1 | 0.527 | 0.951 | 0.524 |
| Stacking | Improvement | 47 | 181 | 34 (72.3) / 7 (14.9) / 5 (10.6) / 1 (2.1) | 0.783 | 0.723 | 0.73 | 0.188 | 0.968 | 0.298 | Estimable | 0.1 | 0.277 |  |  |  |  |  |  |  |  |  |  |  |  |
|  | Stable | 329 | 170 | 118 (35.9) / 129 (39.2) / 64 (19.5) / 18 (5.5) | 0.758 | 0.392 | 0.844 | 0.759 | 0.526 | 0.517 | Estimable | 0.313 | 0.905 |  |  |  |  |  |  |  |  |  |  |  |  |
|  | Mild Progression | 130 | 166 | 23 (17.7) / 14 (10.8) / 74 (56.9) / 19 (14.6) | 0.764 | 0.569 | 0.801 | 0.446 | 0.869 | 0.5 | Estimable | 0.152 | 0.45 |  |  |  |  |  |  |  |  |  |  |  |  |
|  | Rapid Progression | 86 | 75 | 6 (7.0) / 20 (23.3) / 23 (26.7) / 37 (43.0) | 0.816 | 0.43 | 0.925 | 0.493 | 0.905 | 0.46 | Estimable | 0.113 | 0.791 |  |  |  |  |  |  |  |  |  |  |  |  |
|  | All classes | 592 | 592 |  | 0.78 |  |  |  |  |  |  |  |  | 0.78 | 0.751 | TRUE | 0.463 | 0.529 | 0.27 | 0.444 | 0.444 | 0 | 0.488 | 1.238 | 0.678 |

**Notes:** The four class rows of each model give one-versus-rest discrimination, the classification metrics at the argmax decision rule, the confusion breakdown and the DeLong comparison against XGBoost, the primary model; the "All classes" row of each model gives the model-level summary, so the columns from Macro-AUC onward are populated on that row only. The confusion cell gives the number of participants of that true class assigned to each of the four predicted classes in the order Improvement, Stable, Mild Progression, Rapid Progression, with the row percentage in parentheses; the four counts therefore sum to N true and the four percentages to 100%. Macro-AUC is the unweighted mean of the four one-versus-rest AUCs, and equals the mean of the per-class AUC column for every model; micro-AUC pools all participant-by-class pairs into a single curve and is dominated by the majority class, so the two are reported side by side rather than interchangeably. Macro-F1 is averaged over all four classes with F1 set to zero when a class exists in the test set but is never predicted, which applies only to the Improvement class under the support vector machine; the macro-F1 computed over estimable classes alone is given beside it. Four of the twenty DeLong comparisons reached nominal significance.

**Table S9. Calibration before and after frozen recalibration, internal test set and external validation cohort.**

| **Cohort** | **Model** | **Probabilities** | **Class** | **Calibration slope** | **CITL** | **Observed** | **Expected** | **O/E ratio** | **Brier, overall** | **Macro-AUC** |
| --- | --- | --- | --- | --- | --- | --- | --- | --- | --- | --- |
| Internal | Multinomial LR | Raw | Improvement | 1.424 | -1.314 | 0.079 | 0.261 | 0.304 |  |  |
|  |  |  | Stable | 1.749 | 2.022 | 0.556 | 0.276 | 2.01 |  |  |
|  |  |  | Mild Progression | 1.565 | 0.463 | 0.22 | 0.238 | 0.921 |  |  |
|  |  |  | Rapid Progression | 1.658 | -0.007 | 0.145 | 0.224 | 0.649 |  |  |
|  |  |  | All classes |  |  |  |  |  | 0.6557 | 0.768 |
| Internal | Multinomial LR | Platt (frozen) | Improvement | 1.093 | 0.083 | 0.079 | 0.086 | 0.923 |  |  |
|  |  |  | Stable | 1.267 | -0.183 | 0.556 | 0.57 | 0.974 |  |  |
|  |  |  | Mild Progression | 0.875 | -0.061 | 0.22 | 0.208 | 1.057 |  |  |
|  |  |  | Rapid Progression | 1.345 | 0.61 | 0.145 | 0.136 | 1.069 |  |  |
|  |  |  | All classes |  |  |  |  |  | 0.5131 | 0.771 |
| Internal | Multinomial LR | Isotonic (frozen) | Improvement | 1.075 | 0.056 | 0.079 | 0.085 | 0.93 |  |  |
|  |  |  | Stable | 1.234 | -0.2 | 0.556 | 0.575 | 0.966 |  |  |
|  |  |  | Mild Progression | 0.817 | -0.113 | 0.22 | 0.206 | 1.063 |  |  |
|  |  |  | Rapid Progression | 1.398 | 0.762 | 0.145 | 0.133 | 1.093 |  |  |
|  |  |  | All classes |  |  |  |  |  | 0.5033 | 0.775 |
| Internal | RF | Raw | Improvement | 1.184 | -0.726 | 0.079 | 0.17 | 0.466 |  |  |
|  |  |  | Stable | 1.47 | 1.174 | 0.556 | 0.362 | 1.536 |  |  |
|  |  |  | Mild Progression | 1.109 | -0.124 | 0.22 | 0.253 | 0.867 |  |  |
|  |  |  | Rapid Progression | 1.679 | 0.198 | 0.145 | 0.215 | 0.677 |  |  |
|  |  |  | All classes |  |  |  |  |  | 0.5655 | 0.771 |
| Internal | RF | Platt (frozen) | Improvement | 0.922 | -0.222 | 0.079 | 0.084 | 0.945 |  |  |
|  |  |  | Stable | 1.126 | -0.087 | 0.556 | 0.564 | 0.985 |  |  |
|  |  |  | Mild Progression | 0.815 | -0.123 | 0.22 | 0.21 | 1.047 |  |  |
|  |  |  | Rapid Progression | 1.156 | 0.286 | 0.145 | 0.142 | 1.024 |  |  |
|  |  |  | All classes |  |  |  |  |  | 0.5041 | 0.769 |
| Internal | RF | Isotonic (frozen) | Improvement | 0.79 | -0.49 | 0.079 | 0.086 | 0.928 |  |  |
|  |  |  | Stable | 1.073 | -0.058 | 0.556 | 0.562 | 0.988 |  |  |
|  |  |  | Mild Progression | 0.715 | -0.217 | 0.22 | 0.211 | 1.041 |  |  |
|  |  |  | Rapid Progression | 1.104 | 0.21 | 0.145 | 0.141 | 1.03 |  |  |
|  |  |  | All classes |  |  |  |  |  | 0.5055 | 0.768 |
| Internal | XGBoost | Raw | Improvement | 1.1 | -1.198 | 0.079 | 0.209 | 0.379 |  |  |
|  |  |  | Stable | 1.346 | 1.38 | 0.556 | 0.324 | 1.715 |  |  |
|  |  |  | Mild Progression | 0.941 | -0.239 | 0.22 | 0.248 | 0.887 |  |  |
|  |  |  | Rapid Progression | 1.481 | -0.134 | 0.145 | 0.219 | 0.663 |  |  |
|  |  |  | All classes |  |  |  |  |  | 0.5932 | 0.786 |
| Internal | XGBoost | Platt (frozen) | Improvement | 0.981 | -0.201 | 0.079 | 0.091 | 0.872 |  |  |
|  |  |  | Stable | 1.254 | -0.04 | 0.556 | 0.551 | 1.009 |  |  |
|  |  |  | Mild Progression | 0.859 | -0.083 | 0.22 | 0.211 | 1.039 |  |  |
|  |  |  | Rapid Progression | 1.204 | 0.287 | 0.145 | 0.147 | 0.988 |  |  |
|  |  |  | All classes |  |  |  |  |  | 0.5031 | 0.785 |
| Internal | XGBoost | Isotonic (frozen) | Improvement | 0.915 | -0.342 | 0.079 | 0.093 | 0.856 |  |  |
|  |  |  | Stable | 1.161 | -0.011 | 0.556 | 0.549 | 1.012 |  |  |
|  |  |  | Mild Progression | 0.73 | -0.195 | 0.22 | 0.212 | 1.036 |  |  |
|  |  |  | Rapid Progression | 1.141 | 0.203 | 0.145 | 0.146 | 0.995 |  |  |
|  |  |  | All classes |  |  |  |  |  | 0.504 | 0.781 |
| Internal | GBM | Raw | Improvement | 0.979 | -1.301 | 0.079 | 0.206 | 0.386 |  |  |
|  |  |  | Stable | 1.198 | 1.256 | 0.556 | 0.33 | 1.682 |  |  |
|  |  |  | Mild Progression | 0.835 | -0.309 | 0.22 | 0.244 | 0.9 |  |  |
|  |  |  | Rapid Progression | 1.253 | -0.365 | 0.145 | 0.22 | 0.661 |  |  |
|  |  |  | All classes |  |  |  |  |  | 0.5966 | 0.777 |
| Internal | GBM | Platt (frozen) | Improvement | 1.002 | -0.119 | 0.079 | 0.088 | 0.904 |  |  |
|  |  |  | Stable | 1.356 | -0.105 | 0.556 | 0.559 | 0.995 |  |  |
|  |  |  | Mild Progression | 0.888 | -0.037 | 0.22 | 0.208 | 1.057 |  |  |
|  |  |  | Rapid Progression | 1.193 | 0.289 | 0.145 | 0.146 | 0.996 |  |  |
|  |  |  | All classes |  |  |  |  |  | 0.5083 | 0.778 |
| Internal | GBM | Isotonic (frozen) | Improvement | 0.959 | -0.192 | 0.079 | 0.087 | 0.91 |  |  |
|  |  |  | Stable | 1.268 | -0.089 | 0.556 | 0.559 | 0.995 |  |  |
|  |  |  | Mild Progression | 0.735 | -0.178 | 0.22 | 0.207 | 1.06 |  |  |
|  |  |  | Rapid Progression | 1.079 | 0.107 | 0.145 | 0.147 | 0.988 |  |  |
|  |  |  | All classes |  |  |  |  |  | 0.509 | 0.777 |
| Internal | SVM | Raw  Raw  Raw  Raw  Raw | Improvement | 1.058 | 0.149 | 0.079 | 0.078 | 1.02 |  |  |
|  |  |  | Stable | 1.048 | -0.076 | 0.556 | 0.567 | 0.98 |  |  |
|  |  |  | Mild Progression | 0.814 | -0.169 | 0.22 | 0.215 | 1.024 |  |  |
|  |  |  | Rapid Progression | 1.365 | 0.59 | 0.145 | 0.14 | 1.035 |  |  |
|  |  |  | All classes |  |  |  |  |  | 0.5237 | 0.75 |
| Internal | SVM | Platt (frozen) | Improvement | 0.97 | -0.052 | 0.079 | 0.079 | 1.011 |  |  |
|  |  |  | Stable | 1.086 | -0.114 | 0.556 | 0.572 | 0.972 |  |  |
|  |  |  | Mild Progression | 0.838 | -0.122 | 0.22 | 0.211 | 1.041 |  |  |
|  |  |  | Rapid Progression | 1.31 | 0.527 | 0.145 | 0.138 | 1.049 |  |  |
|  |  |  | All classes |  |  |  |  |  | 0.5232 | 0.75 |
| Internal | SVM | Isotonic (frozen) | Improvement | 0.924 | -0.148 | 0.079 | 0.079 | 1.01 |  |  |
|  |  |  | Stable | 0.949 | -0.067 | 0.556 | 0.573 | 0.97 |  |  |
|  |  |  | Mild Progression | 0.638 | -0.314 | 0.22 | 0.21 | 1.045 |  |  |
|  |  |  | Rapid Progression | 1.324 | 0.557 | 0.145 | 0.138 | 1.049 |  |  |
|  |  |  | All classes |  |  |  |  |  | 0.5176 | 0.756 |
| Internal | Stacking | Raw | Improvement | 2.371 | -0.328 | 0.079 | 0.258 | 0.308 |  |  |
|  |  |  | Stable | 3.823 | 4.308 | 0.556 | 0.259 | 2.145 |  |  |
|  |  |  | Mild Progression | 2.192 | 1.078 | 0.22 | 0.243 | 0.903 |  |  |
|  |  |  | Rapid Progression | 3.255 | 1.651 | 0.145 | 0.24 | 0.606 |  |  |
|  |  |  | All classes |  |  |  |  |  | 0.6783 | 0.78 |
| Internal | Stacking | Platt (frozen) | Improvement | 1.012 | -0.059 | 0.079 | 0.085 | 0.933 |  |  |
|  |  |  | Stable | 1.111 | -0.072 | 0.556 | 0.563 | 0.988 |  |  |
|  |  |  | Mild Progression | 0.852 | -0.094 | 0.22 | 0.211 | 1.04 |  |  |
|  |  |  | Rapid Progression | 1.271 | 0.468 | 0.145 | 0.141 | 1.03 |  |  |
|  |  |  | All classes |  |  |  |  |  | 0.503 | 0.779 |
| Internal | Stacking | Isotonic (frozen) | Improvement | 0.988 | -0.122 | 0.079 | 0.086 | 0.921 |  |  |
|  |  |  | Stable | 1.027 | -0.054 | 0.556 | 0.565 | 0.984 |  |  |
|  |  |  | Mild Progression | 0.759 | -0.155 | 0.22 | 0.208 | 1.056 |  |  |
|  |  |  | Rapid Progression | 1.246 | 0.434 | 0.145 | 0.141 | 1.029 |  |  |
|  |  |  | All classes |  |  |  |  |  | 0.504 | 0.776 |
| External | Multinomial LR | Raw | Improvement | 0.833 | -1.209 | 0.065 | 0.147 | 0.443 |  |  |
|  |  |  | Stable | 1.039 | 0.52 | 0.381 | 0.286 | 1.332 |  |  |
|  |  |  | Mild Progression | 0.841 | 0.193 | 0.364 | 0.296 | 1.231 |  |  |
|  |  |  | Rapid Progression | 1.958 | 0.137 | 0.189 | 0.27 | 0.699 |  |  |
|  |  |  | All classes |  |  |  |  |  | 0.6387 |  |
| External | Multinomial LR | Platt (frozen) | Improvement | 0.627 | -0.416 | 0.065 | 0.033 | 1.957 |  |  |
|  |  |  | Stable | 0.814 | -0.64 | 0.381 | 0.519 | 0.734 |  |  |
|  |  |  | Mild Progression | 0.446 | -0.078 | 0.364 | 0.281 | 1.298 |  |  |
|  |  |  | Rapid Progression | 1.504 | 0.84 | 0.189 | 0.167 | 1.135 |  |  |
|  |  |  | All classes |  |  |  |  |  | 0.6378 |  |
| External | Multinomial LR | Isotonic (frozen) | Improvement | 0.551 | -0.611 | 0.065 | 0.032 | 2.026 |  |  |
|  |  |  | Stable | 0.846 | -0.649 | 0.381 | 0.518 | 0.736 |  |  |
|  |  |  | Mild Progression | 0.468 | -0.071 | 0.364 | 0.289 | 1.26 |  |  |
|  |  |  | Rapid Progression | 1.28 | 0.644 | 0.189 | 0.16 | 1.18 |  |  |
|  |  |  | All classes |  |  |  |  |  | 0.6331 |  |
| External | RF | Raw | Improvement | 1.039 | -0.547 | 0.065 | 0.111 | 0.588 |  |  |
|  |  |  | Stable | 1.737 | 0.68 | 0.381 | 0.334 | 1.143 |  |  |
|  |  |  | Mild Progression | 0.83 | -0.048 | 0.364 | 0.356 | 1.022 |  |  |
|  |  |  | Rapid Progression | 1.234 | 0.208 | 0.189 | 0.199 | 0.95 |  |  |
|  |  |  | All classes |  |  |  |  |  | 0.5929 |  |
| External | RF | Platt (frozen) | Improvement | 0.781 | -0.162 | 0.065 | 0.044 | 1.481 |  |  |
|  |  |  | Stable | 1.343 | -0.65 | 0.381 | 0.504 | 0.757 |  |  |
|  |  |  | Mild Progression | 0.616 | -0.021 | 0.364 | 0.317 | 1.15 |  |  |
|  |  |  | Rapid Progression | 0.842 | 0.272 | 0.189 | 0.135 | 1.399 |  |  |
|  |  |  | All classes |  |  |  |  |  | 0.6078 |  |
| External | RF | Isotonic (frozen) | Improvement | 0.5 | -0.89 | 0.065 | 0.042 | 1.566 |  |  |
|  |  |  | Stable | 1.32 | -0.596 | 0.381 | 0.495 | 0.77 |  |  |
|  |  |  | Mild Progression | 0.563 | -0.084 | 0.364 | 0.33 | 1.104 |  |  |
|  |  |  | Rapid Progression | 0.789 | 0.217 | 0.189 | 0.133 | 1.422 |  |  |
|  |  |  | All classes |  |  |  |  |  | 0.6021 |  |
| External | XGBoost | Raw | Improvement | 0.953 | -0.91 | 0.065 | 0.132 | 0.494 |  |  |
|  |  |  | Stable | 1.386 | 0.752 | 0.381 | 0.292 | 1.305 |  |  |
|  |  |  | Mild Progression | 0.737 | -0.184 | 0.364 | 0.381 | 0.956 |  |  |
|  |  |  | Rapid Progression | 1.17 | 0.145 | 0.189 | 0.195 | 0.971 |  |  |
|  |  |  | All classes |  |  |  |  |  | 0.6106 |  |
| External | XGBoost | Platt (frozen) | Improvement | 0.869 | 0.014 | 0.065 | 0.047 | 1.399 |  |  |
|  |  |  | Stable | 1.287 | -0.555 | 0.381 | 0.491 | 0.777 |  |  |
|  |  |  | Mild Progression | 0.654 | -0.053 | 0.364 | 0.331 | 1.1 |  |  |
|  |  |  | Rapid Progression | 0.945 | 0.474 | 0.189 | 0.132 | 1.437 |  |  |
|  |  |  | All classes |  |  |  |  |  | 0.6136 |  |
| External | XGBoost | Isotonic (frozen) | Improvement | 0.707 | -0.385 | 0.065 | 0.045 | 1.449 |  |  |
|  |  |  | Stable | 1.224 | -0.561 | 0.381 | 0.492 | 0.776 |  |  |
|  |  |  | Mild Progression | 0.644 | -0.063 | 0.364 | 0.334 | 1.09 |  |  |
|  |  |  | Rapid Progression | 0.873 | 0.405 | 0.189 | 0.129 | 1.468 |  |  |
|  |  |  | All classes |  |  |  |  |  | 0.6098 |  |
| External | GBM | Raw | Improvement | 0.65 | -1.323 | 0.065 | 0.119 | 0.549 |  |  |
|  |  |  | Stable | 1.206 | 0.48 | 0.381 | 0.315 | 1.21 |  |  |
|  |  |  | Mild Progression | 0.639 | -0.183 | 0.364 | 0.367 | 0.993 |  |  |
|  |  |  | Rapid Progression | 1.137 | 0.09 | 0.189 | 0.199 | 0.95 |  |  |
|  |  |  | All classes |  |  |  |  |  | 0.6194 |  |
| External | GBM | Platt (frozen) | Improvement | 0.674 | -0.518 | 0.065 | 0.044 | 1.468 |  |  |
|  |  |  | Stable | 1.419 | -0.748 | 0.381 | 0.523 | 0.729 |  |  |
|  |  |  | Mild Progression | 0.67 | 0.042 | 0.364 | 0.304 | 1.198 |  |  |
|  |  |  | Rapid Progression | 1.073 | 0.688 | 0.189 | 0.128 | 1.472 |  |  |
|  |  |  | All classes |  |  |  |  |  | 0.6336 |  |
| External | GBM | Isotonic (frozen) | Improvement | 0.633 | -0.574 | 0.065 | 0.043 | 1.533 |  |  |
|  |  |  | Stable | 1.372 | -0.716 | 0.381 | 0.519 | 0.735 |  |  |
|  |  |  | Mild Progression | 0.669 | 0.025 | 0.364 | 0.31 | 1.174 |  |  |
|  |  |  | Rapid Progression | 1.125 | 0.791 | 0.189 | 0.128 | 1.478 |  |  |
|  |  |  | All classes |  |  |  |  |  | 0.6305 |  |
| External | SVM | Raw | Improvement | 0.338 | -1.607 | 0.065 | 0.051 | 1.268 |  |  |
|  |  |  | Stable | 0.768 | -0.766 | 0.381 | 0.559 | 0.682 |  |  |
|  |  |  | Mild Progression | 0.332 | -0.112 | 0.364 | 0.238 | 1.531 |  |  |
|  |  |  | Rapid Progression | 1.428 | 0.946 | 0.189 | 0.151 | 1.251 |  |  |
|  |  |  | All classes |  |  |  |  |  | 0.6838 |  |
| External | SVM | Platt (frozen) | Improvement | 0.317 | -1.646 | 0.065 | 0.05 | 1.303 |  |  |
|  |  |  | Stable | 0.779 | -0.795 | 0.381 | 0.566 | 0.674 |  |  |
|  |  |  | Mild Progression | 0.344 | -0.091 | 0.364 | 0.234 | 1.556 |  |  |
|  |  |  | Rapid Progression | 1.375 | 0.885 | 0.189 | 0.15 | 1.264 |  |  |
|  |  |  | All classes |  |  |  |  |  | 0.686 |  |
| External | SVM | Isotonic (frozen) | Improvement | 0.356 | -1.478 | 0.065 | 0.046 | 1.415 |  |  |
|  |  |  | Stable | 0.712 | -0.759 | 0.381 | 0.563 | 0.678 |  |  |
|  |  |  | Mild Progression | 0.126 | -0.353 | 0.364 | 0.243 | 1.501 |  |  |
|  |  |  | Rapid Progression | 1.287 | 0.784 | 0.189 | 0.149 | 1.272 |  |  |
|  |  |  | All classes |  |  |  |  |  | 0.6817 |  |
| External | Stacking | Raw | Improvement | 1.984 | -0.251 | 0.065 | 0.212 | 0.308 |  |  |
|  |  |  | Stable | 4.064 | 4.014 | 0.381 | 0.245 | 1.559 |  |  |
|  |  |  | Mild Progression | 1.703 | 0.924 | 0.364 | 0.294 | 1.239 |  |  |
|  |  |  | Rapid Progression | 3.123 | 1.782 | 0.189 | 0.249 | 0.759 |  |  |
|  |  |  | All classes |  |  |  |  |  | 0.6755 |  |
| External | Stacking | Platt (frozen) | Improvement | 0.837 | -0.029 | 0.065 | 0.045 | 1.459 |  |  |
|  |  |  | Stable | 1.242 | -0.569 | 0.381 | 0.491 | 0.776 |  |  |
|  |  |  | Mild Progression | 0.656 | 0.015 | 0.364 | 0.312 | 1.167 |  |  |
|  |  |  | Rapid Progression | 1.208 | 0.648 | 0.189 | 0.152 | 1.244 |  |  |
|  |  |  | All classes |  |  |  |  |  | 0.6064 |  |
| External | Stacking | Isotonic (frozen) | Improvement | 0.795 | -0.155 | 0.065 | 0.046 | 1.411 |  |  |
|  |  |  | Stable | 1.198 | -0.503 | 0.381 | 0.481 | 0.793 |  |  |
|  |  |  | Mild Progression | 0.639 | -0.015 | 0.364 | 0.321 | 1.136 |  |  |
|  |  |  | Rapid Progression | 1.242 | 0.7 | 0.189 | 0.152 | 1.243 |  |  |
|  |  |  | All classes |  |  |  |  |  | 0.5999 |  |

**Notes:** Calibration was first assessed on the untransformed predicted probabilities, then recalibration was performed by Platt scaling and, separately, by isotonic regression, each fitted exclusively on out-of-fold predictions from the CHARLS training set and applied unchanged to the test set and to the external cohort. No calibration model was fitted on the data used to evaluate it, so the reported calibration is not optimistic. The four class rows of each model-and-calibration block give the class-specific calibration and the "All classes" row gives the multiclass Brier score and, internally, the Macro-AUC; Macro-AUC was not recomputed externally. A calibration slope of 1.0 and an O/E ratio of 1.0 indicate perfect calibration, and slopes below 1.0 indicate predictions that are too extreme. Internally the recalibration worked as intended, bringing every slope within 0.815 to 1.356 and every observed-to-expected ratio within 0.872 to 1.069 under Platt scaling while leaving Macro-AUC unchanged to within 0.007. Externally it did not: slopes spread from 0.317 to 1.504 and the multiclass Brier score did not improve, so recalibration constants estimated in a community cohort with a fixed four-year interval do not transport to a hospital cohort with a shorter and more variable one. The 1,888-row decision-curve table is not reproduced here; the curves are shown in Figure S5 and the table is supplied in the machine-readable supplement. O/E, observed to expected.

**Table S10. Threshold-level performance and screening yield for the identification of rapid progression.**

| **Section** | **Cohort** | **Class** | **Prevalence** | **Model** | **Operating point** | **Threshold** | **N** | **N events** | **Sens.** | **Spec.** | **PPV** | **NPV** | **F1** | **TP** | **FP** | **FN** | **TN** | **N flagged** | **% flagged** | **N screened per case** | **Net benefit** | **Internal Sens., ref** | **Internal PPV, ref** | **Missed cases, %** | **Enrichment** |
| --- | --- | --- | --- | --- | --- | --- | --- | --- | --- | --- | --- | --- | --- | --- | --- | --- | --- | --- | --- | --- | --- | --- | --- | --- | --- |
| Operating points | Internal | Rapid Prog. | 0.145 | Multinomial LR | Youden-optimal | 0.37 | 592 | 86 | 0.581 | 0.913 | 0.532 | 0.928 | 0.556 | 50 | 44 | 36 | 462 | 94 | 0.159 | 1.88 | 0.041 |  |  |  |  |
| Operating points | Internal | Rapid Prog. | 0.145 | Multinomial LR | Sens. >= 0.90 | 0.15 | 592 | 86 | 0.907 | 0.417 | 0.209 | 0.963 | 0.34 | 78 | 295 | 8 | 211 | 373 | 0.63 | 4.782 | 0.044 |  |  |  |  |
| Operating points | Internal | Rapid Prog. | 0.145 | Multinomial LR | Sens. >= 0.80 | 0.2 | 592 | 86 | 0.837 | 0.589 | 0.257 | 0.955 | 0.393 | 72 | 208 | 14 | 298 | 280 | 0.473 | 3.889 | 0.034 |  |  |  |  |
| Operating points | Internal | Rapid Prog. | 0.145 | Multinomial LR | Prevalence | 0.15 | 592 | 86 | 0.907 | 0.417 | 0.209 | 0.963 | 0.34 | 78 | 295 | 8 | 211 | 373 | 0.63 | 4.782 | 0.044 |  |  |  |  |
| Operating points | Internal | Rapid Prog. | 0.145 | RF | Youden-optimal | 0.23 | 592 | 86 | 0.64 | 0.828 | 0.387 | 0.931 | 0.482 | 55 | 87 | 31 | 419 | 142 | 0.24 | 2.582 | 0.049 |  |  |  |  |
| Operating points | Internal | Rapid Prog. | 0.145 | RF | Sens. >= 0.90 | 0.15 | 592 | 86 | 0.919 | 0.342 | 0.192 | 0.961 | 0.317 | 79 | 333 | 7 | 173 | 412 | 0.696 | 5.215 | 0.034 |  |  |  |  |
| Operating points | Internal | Rapid Prog. | 0.145 | RF | Sens. >= 0.80 | 0.17 | 592 | 86 | 0.837 | 0.488 | 0.218 | 0.946 | 0.345 | 72 | 259 | 14 | 247 | 331 | 0.559 | 4.597 | 0.032 |  |  |  |  |
| Operating points | Internal | Rapid Prog. | 0.145 | RF | Prevalence | 0.15 | 592 | 86 | 0.919 | 0.342 | 0.192 | 0.961 | 0.317 | 79 | 333 | 7 | 173 | 412 | 0.696 | 5.215 | 0.034 |  |  |  |  |
| Operating points | Internal | Rapid Prog. | 0.145 | XGBoost | Youden-optimal | 0.23 | 592 | 86 | 0.767 | 0.739 | 0.333 | 0.949 | 0.465 | 66 | 132 | 20 | 374 | 198 | 0.334 | 3 | 0.045 |  |  |  |  |
| Operating points | Internal | Rapid Prog. | 0.145 | XGBoost | Sens. >= 0.90 | 0.15 | 592 | 86 | 0.907 | 0.407 | 0.206 | 0.963 | 0.336 | 78 | 300 | 8 | 206 | 378 | 0.639 | 4.846 | 0.042 |  |  |  |  |
| Operating points | Internal | Rapid Prog. | 0.145 | XGBoost | Sens. >= 0.80 | 0.2 | 592 | 86 | 0.802 | 0.652 | 0.282 | 0.951 | 0.417 | 69 | 176 | 17 | 330 | 245 | 0.414 | 3.551 | 0.042 |  |  |  |  |
| Operating points | Internal | Rapid Prog. | 0.145 | XGBoost | Prevalence | 0.15 | 592 | 86 | 0.907 | 0.407 | 0.206 | 0.963 | 0.336 | 78 | 300 | 8 | 206 | 378 | 0.639 | 4.846 | 0.042 |  |  |  |  |
| Operating points | Internal | Rapid Prog. | 0.145 | GBM | Youden-optimal | 0.23 | 592 | 86 | 0.721 | 0.761 | 0.339 | 0.941 | 0.461 | 62 | 121 | 24 | 385 | 183 | 0.309 | 2.952 | 0.044 |  |  |  |  |
| Operating points | Internal | Rapid Prog. | 0.145 | GBM | Sens. >= 0.90 | 0.14 | 592 | 86 | 0.907 | 0.407 | 0.206 | 0.963 | 0.336 | 78 | 300 | 8 | 206 | 378 | 0.639 | 4.846 | 0.049 |  |  |  |  |
| Operating points | Internal | Rapid Prog. | 0.145 | GBM | Sens. >= 0.80 | 0.19 | 592 | 86 | 0.802 | 0.64 | 0.275 | 0.95 | 0.409 | 69 | 182 | 17 | 324 | 251 | 0.424 | 3.638 | 0.044 |  |  |  |  |
| Operating points | Internal | Rapid Prog. | 0.145 | GBM | Prevalence | 0.15 | 592 | 86 | 0.884 | 0.47 | 0.221 | 0.96 | 0.353 | 76 | 268 | 10 | 238 | 344 | 0.581 | 4.526 | 0.048 |  |  |  |  |
| Operating points | Internal | Rapid Prog. | 0.145 | SVM | Youden-optimal | 0.2 | 592 | 86 | 0.64 | 0.838 | 0.401 | 0.932 | 0.493 | 55 | 82 | 31 | 424 | 137 | 0.231 | 2.491 | 0.058 |  |  |  |  |
| Operating points | Internal | Rapid Prog. | 0.145 | SVM | Sens. >= 0.90 | 0.07 | 592 | 86 | 0.907 | 0.35 | 0.192 | 0.957 | 0.316 | 78 | 329 | 8 | 177 | 407 | 0.688 | 5.218 | 0.09 |  |  |  |  |
| Operating points | Internal | Rapid Prog. | 0.145 | SVM | Sens. >= 0.80 | 0.1 | 592 | 86 | 0.826 | 0.534 | 0.231 | 0.947 | 0.361 | 71 | 236 | 15 | 270 | 307 | 0.519 | 4.324 | 0.076 |  |  |  |  |
| Operating points | Internal | Rapid Prog. | 0.145 | SVM | Prevalence | 0.15 | 592 | 86 | 0.686 | 0.739 | 0.309 | 0.933 | 0.426 | 59 | 132 | 27 | 374 | 191 | 0.323 | 3.237 | 0.06 |  |  |  |  |
| Operating points | Internal | Rapid Prog. | 0.145 | Stacking | Youden-optimal | 0.26 | 592 | 86 | 0.663 | 0.848 | 0.425 | 0.937 | 0.518 | 57 | 77 | 29 | 429 | 134 | 0.226 | 2.351 | 0.051 |  |  |  |  |
| Operating points | Internal | Rapid Prog. | 0.145 | Stacking | Sens. >= 0.90 | 0.21 | 592 | 86 | 0.919 | 0.439 | 0.218 | 0.969 | 0.352 | 79 | 284 | 7 | 222 | 363 | 0.613 | 4.595 | 0.006 |  |  |  |  |
| Operating points | Internal | Rapid Prog. | 0.145 | Stacking | Sens. >= 0.80 | 0.24 | 592 | 86 | 0.802 | 0.66 | 0.286 | 0.952 | 0.422 | 69 | 172 | 17 | 334 | 241 | 0.407 | 3.493 | 0.025 |  |  |  |  |
| Operating points | Internal | Rapid Prog. | 0.145 | Stacking | Prevalence | 0.15 | 592 | 86 | 0.988 | 0.024 | 0.147 | 0.923 | 0.256 | 85 | 494 | 1 | 12 | 579 | 0.978 | 6.812 | -0.004 |  |  |  |  |
| Operating points | Internal | Improvement | 0.079 | XGBoost | Youden-optimal | 0.24 | 592 | 47 | 0.787 | 0.705 | 0.187 | 0.975 | 0.302 | 37 | 161 | 10 | 384 | 198 | 0.334 | 5.351 | -0.023 |  |  |  |  |
| Operating points | Internal | Improvement | 0.079 | XGBoost | Sens. >= 0.90 | 0.13 | 592 | 47 | 0.915 | 0.442 | 0.124 | 0.984 | 0.218 | 43 | 304 | 4 | 241 | 347 | 0.586 | 8.07 | -0.004 |  |  |  |  |
| Operating points | Internal | Improvement | 0.079 | XGBoost | Sens. >= 0.80 | 0.21 | 592 | 47 | 0.851 | 0.637 | 0.168 | 0.98 | 0.281 | 40 | 198 | 7 | 347 | 238 | 0.402 | 5.95 | -0.021 |  |  |  |  |
| Operating points | Internal | Improvement | 0.079 | XGBoost | Prevalence | 0.15 | 592 | 47 | 0.894 | 0.486 | 0.13 | 0.981 | 0.228 | 42 | 280 | 5 | 265 | 322 | 0.544 | 7.667 | -0.013 |  |  |  |  |
| Operating points | Internal | Stable | 0.556 | XGBoost | Youden-optimal | 0.31 | 592 | 329 | 0.729 | 0.688 | 0.745 | 0.67 | 0.737 | 240 | 82 | 89 | 181 | 322 | 0.544 | 1.342 | 0.343 |  |  |  |  |
| Operating points | Internal | Stable | 0.556 | XGBoost | Sens. >= 0.90 | 0.21 | 592 | 329 | 0.906 | 0.414 | 0.659 | 0.779 | 0.763 | 298 | 154 | 31 | 109 | 452 | 0.764 | 1.517 | 0.434 |  |  |  |  |
| Operating points | Internal | Stable | 0.556 | XGBoost | Sens. >= 0.80 | 0.27 | 592 | 329 | 0.818 | 0.574 | 0.706 | 0.716 | 0.758 | 269 | 112 | 60 | 151 | 381 | 0.644 | 1.416 | 0.384 |  |  |  |  |
| Operating points | Internal | Stable | 0.556 | XGBoost | Prevalence | 0.15 | 592 | 329 | 0.954 | 0.27 | 0.621 | 0.826 | 0.752 | 314 | 192 | 15 | 71 | 506 | 0.855 | 1.611 | 0.473 |  |  |  |  |
| Operating points | Internal | Mild Prog. | 0.220 | XGBoost | Youden-optimal | 0.26 | 592 | 130 | 0.731 | 0.673 | 0.386 | 0.899 | 0.505 | 95 | 151 | 35 | 311 | 246 | 0.416 | 2.589 | 0.071 |  |  |  |  |
| Operating points | Internal | Mild Prog. | 0.220 | XGBoost | Sens. >= 0.90 | 0.13 | 592 | 130 | 0.908 | 0.494 | 0.335 | 0.95 | 0.49 | 118 | 234 | 12 | 228 | 352 | 0.595 | 2.983 | 0.14 |  |  |  |  |
| Operating points | Internal | Mild Prog. | 0.220 | XGBoost | Sens. >= 0.80 | 0.2 | 592 | 130 | 0.8 | 0.591 | 0.355 | 0.913 | 0.492 | 104 | 189 | 26 | 273 | 293 | 0.495 | 2.817 | 0.096 |  |  |  |  |
| Operating points | Internal | Mild Prog. | 0.220 | XGBoost | Prevalence | 0.15 | 592 | 130 | 0.854 | 0.515 | 0.331 | 0.926 | 0.477 | 111 | 224 | 19 | 238 | 335 | 0.566 | 3.018 | 0.121 |  |  |  |  |
| Operating points | External | Rapid Prog. | 0.189 | XGBoost (parsimonious) | Internally derived, Youden-optimal | 0.26 | 291 | 55 | 0.709 | 0.729 | 0.379 | 0.915 | 0.494 | 39 | 64 | 16 | 172 | 103 | 0.354 | 2.64 | 0.057 | 0.640 | 0.387 | 29.1 |  |
| Operating points | External | Rapid Prog. | 0.189 | XGBoost (parsimonious) | Internally derived, Sens. >= 0.90 | 0.15 | 291 | 55 | 0.927 | 0.369 | 0.255 | 0.956 | 0.400 | 51 | 149 | 4 | 87 | 200 | 0.687 | 3.92 | 0.085 | 0.907 | 0.188 | 7.3 |  |
| Operating points | External | Rapid Prog. | 0.189 | XGBoost (parsimonious) | Internally derived, Sens. >= 0.80 | 0.18 | 291 | 55 | 0.927 | 0.403 | 0.266 | 0.960 | 0.413 | 51 | 141 | 4 | 95 | 192 | 0.660 | 3.76 | 0.069 | 0.837 | 0.228 | 7.3 |  |
| Operating points | External | Rapid Prog. | 0.189 | XGBoost (parsimonious) | Internally derived, prevalence | 0.15 | 291 | 55 | 0.927 | 0.369 | 0.255 | 0.956 | 0.400 | 51 | 149 | 4 | 87 | 200 | 0.687 | 3.92 | 0.085 | 0.907 | 0.188 | 7.3 |  |
| Threshold sweep | Internal | Rapid Prog. | 0.145 | XGBoost | - | 0.05 | 592 | 86 | 1 |  | 0.160 |  |  | 86 | 453 | 0 | 53 | 539 | 0.910 | 6.267 |  |  |  | 0 |  |
| Threshold sweep | Internal | Rapid Prog. | 0.145 | XGBoost | - | 0.06 | 592 | 86 | 1 |  | 0.161 |  |  | 86 | 449 | 0 | 57 | 535 | 0.904 | 6.221 |  |  |  | 0 |  |
| Threshold sweep | Internal | Rapid Prog. | 0.145 | XGBoost | - | 0.07 | 592 | 86 | 1 |  | 0.162 |  |  | 86 | 445 | 0 | 61 | 531 | 0.897 | 6.174 |  |  |  | 0 |  |
| Threshold sweep | Internal | Rapid Prog. | 0.145 | XGBoost | - | 0.08 | 592 | 86 | 0.988 |  | 0.162 |  |  | 85 | 441 | 1 | 65 | 526 | 0.889 | 6.188 |  |  |  | 1.2 |  |
| Threshold sweep | Internal | Rapid Prog. | 0.145 | XGBoost | - | 0.09 | 592 | 86 | 0.988 |  | 0.163 |  |  | 85 | 435 | 1 | 71 | 520 | 0.878 | 6.118 |  |  |  | 1.2 |  |
| Threshold sweep | Internal | Rapid Prog. | 0.145 | XGBoost | - | 0.1 | 592 | 86 | 0.988 |  | 0.168 |  |  | 85 | 422 | 1 | 84 | 507 | 0.856 | 5.965 |  |  |  | 1.2 |  |
| Threshold sweep | Internal | Rapid Prog. | 0.145 | XGBoost | - | 0.11 | 592 | 86 | 0.988 |  | 0.176 |  |  | 85 | 399 | 1 | 107 | 484 | 0.818 | 5.694 |  |  |  | 1.2 |  |
| Threshold sweep | Internal | Rapid Prog. | 0.145 | XGBoost | - | 0.12 | 592 | 86 | 0.977 |  | 0.179 |  |  | 84 | 385 | 2 | 121 | 469 | 0.792 | 5.583 |  |  |  | 2.3 |  |
| Threshold sweep | Internal | Rapid Prog. | 0.145 | XGBoost | - | 0.13 | 592 | 86 | 0.953 |  | 0.187 |  |  | 82 | 356 | 4 | 150 | 438 | 0.740 | 5.341 |  |  |  | 4.7 |  |
| Threshold sweep | Internal | Rapid Prog. | 0.145 | XGBoost | - | 0.14 | 592 | 86 | 0.942 |  | 0.2 |  |  | 81 | 324 | 5 | 182 | 405 | 0.684 | 5 |  |  |  | 5.8 |  |
| Threshold sweep | Internal | Rapid Prog. | 0.145 | XGBoost | - | 0.15 | 592 | 86 | 0.907 |  | 0.206 |  |  | 78 | 300 | 8 | 206 | 378 | 0.639 | 4.846 |  |  |  | 9.3 |  |
| Threshold sweep | Internal | Rapid Prog. | 0.145 | XGBoost | - | 0.16 | 592 | 86 | 0.895 |  | 0.226 |  |  | 77 | 264 | 9 | 242 | 341 | 0.576 | 4.429 |  |  |  | 10.5 |  |
| Threshold sweep | Internal | Rapid Prog. | 0.145 | XGBoost | - | 0.17 | 592 | 86 | 0.884 |  | 0.239 |  |  | 76 | 242 | 10 | 264 | 318 | 0.537 | 4.184 |  |  |  | 11.6 |  |
| Threshold sweep | Internal | Rapid Prog. | 0.145 | XGBoost | - | 0.18 | 592 | 86 | 0.849 |  | 0.253 |  |  | 73 | 216 | 13 | 290 | 289 | 0.488 | 3.959 |  |  |  | 15.1 |  |
| Threshold sweep | Internal | Rapid Prog. | 0.145 | XGBoost | - | 0.19 | 592 | 86 | 0.826 |  | 0.270 |  |  | 71 | 192 | 15 | 314 | 263 | 0.444 | 3.704 |  |  |  | 17.4 |  |
| Threshold sweep | Internal | Rapid Prog. | 0.145 | XGBoost | - | 0.2 | 592 | 86 | 0.802 |  | 0.282 |  |  | 69 | 176 | 17 | 330 | 245 | 0.414 | 3.551 |  |  |  | 19.8 |  |
| Threshold sweep | Internal | Rapid Prog. | 0.145 | XGBoost | - | 0.21 | 592 | 86 | 0.791 |  | 0.304 |  |  | 68 | 156 | 18 | 350 | 224 | 0.378 | 3.294 |  |  |  | 20.9 |  |
| Threshold sweep | Internal | Rapid Prog. | 0.145 | XGBoost | - | 0.22 | 592 | 86 | 0.779 |  | 0.316 |  |  | 67 | 145 | 19 | 361 | 212 | 0.358 | 3.164 |  |  |  | 22.1 |  |
| Threshold sweep | Internal | Rapid Prog. | 0.145 | XGBoost | - | 0.23 | 592 | 86 | 0.767 |  | 0.333 |  |  | 66 | 132 | 20 | 374 | 198 | 0.334 | 3 |  |  |  | 23.3 |  |
| Threshold sweep | Internal | Rapid Prog. | 0.145 | XGBoost | - | 0.24 | 592 | 86 | 0.733 |  | 0.35 |  |  | 63 | 117 | 23 | 389 | 180 | 0.304 | 2.857 |  |  |  | 26.7 |  |
| Threshold sweep | Internal | Rapid Prog. | 0.145 | XGBoost | - | 0.25 | 592 | 86 | 0.686 |  | 0.355 |  |  | 59 | 107 | 27 | 399 | 166 | 0.280 | 2.814 |  |  |  | 31.4 |  |
| Threshold sweep | Internal | Rapid Prog. | 0.145 | XGBoost | - | 0.26 | 592 | 86 | 0.640 |  | 0.374 |  |  | 55 | 92 | 31 | 414 | 147 | 0.248 | 2.673 |  |  |  | 36 |  |
| Threshold sweep | Internal | Rapid Prog. | 0.145 | XGBoost | - | 0.27 | 592 | 86 | 0.628 |  | 0.403 |  |  | 54 | 80 | 32 | 426 | 134 | 0.226 | 2.481 |  |  |  | 37.2 |  |
| Threshold sweep | Internal | Rapid Prog. | 0.145 | XGBoost | - | 0.28 | 592 | 86 | 0.593 |  | 0.418 |  |  | 51 | 71 | 35 | 435 | 122 | 0.206 | 2.392 |  |  |  | 40.7 |  |
| Threshold sweep | Internal | Rapid Prog. | 0.145 | XGBoost | - | 0.29 | 592 | 86 | 0.570 |  | 0.4375 |  |  | 49 | 63 | 37 | 443 | 112 | 0.189 | 2.286 |  |  |  | 43 |  |
| Threshold sweep | Internal | Rapid Prog. | 0.145 | XGBoost | - | 0.3 | 592 | 86 | 0.535 |  | 0.447 |  |  | 46 | 57 | 40 | 449 | 103 | 0.174 | 2.239 |  |  |  | 46.5 |  |
| Threshold sweep | Internal | Rapid Prog. | 0.145 | XGBoost | - | 0.31 | 592 | 86 | 0.512 |  | 0.454 |  |  | 44 | 53 | 42 | 453 | 97 | 0.164 | 2.205 |  |  |  | 48.8 |  |
| Threshold sweep | Internal | Rapid Prog. | 0.145 | XGBoost | - | 0.32 | 592 | 86 | 0.512 |  | 0.463 |  |  | 44 | 51 | 42 | 455 | 95 | 0.160 | 2.159 |  |  |  | 48.8 |  |
| Threshold sweep | Internal | Rapid Prog. | 0.145 | XGBoost | - | 0.33 | 592 | 86 | 0.5 |  | 0.462 |  |  | 43 | 50 | 43 | 456 | 93 | 0.157 | 2.163 |  |  |  | 50 |  |
| Threshold sweep | Internal | Rapid Prog. | 0.145 | XGBoost | - | 0.34 | 592 | 86 | 0.5 |  | 0.5 |  |  | 43 | 43 | 43 | 463 | 86 | 0.145 | 2 |  |  |  | 50 |  |
| Threshold sweep | Internal | Rapid Prog. | 0.145 | XGBoost | - | 0.35 | 592 | 86 | 0.488 |  | 0.538 |  |  | 42 | 36 | 44 | 470 | 78 | 0.132 | 1.857 |  |  |  | 51.2 |  |
| Threshold sweep | Internal | Rapid Prog. | 0.145 | XGBoost | - | 0.36 | 592 | 86 | 0.488 |  | 0.553 |  |  | 42 | 34 | 44 | 472 | 76 | 0.128 | 1.810 |  |  |  | 51.2 |  |
| Threshold sweep | Internal | Rapid Prog. | 0.145 | XGBoost | - | 0.37 | 592 | 86 | 0.465 |  | 0.556 |  |  | 40 | 32 | 46 | 474 | 72 | 0.122 | 1.8 |  |  |  | 53.5 |  |
| Threshold sweep | Internal | Rapid Prog. | 0.145 | XGBoost | - | 0.38 | 592 | 86 | 0.453 |  | 0.557 |  |  | 39 | 31 | 47 | 475 | 70 | 0.118 | 1.795 |  |  |  | 54.7 |  |
| Threshold sweep | Internal | Rapid Prog. | 0.145 | XGBoost | - | 0.39 | 592 | 86 | 0.430 |  | 0.561 |  |  | 37 | 29 | 49 | 477 | 66 | 0.111 | 1.784 |  |  |  | 57 |  |
| Threshold sweep | Internal | Rapid Prog. | 0.145 | XGBoost | - | 0.4 | 592 | 86 | 0.430 |  | 0.569 |  |  | 37 | 28 | 49 | 478 | 65 | 0.110 | 1.757 |  |  |  | 57 |  |
| Threshold sweep | Internal | Rapid Prog. | 0.145 | XGBoost | - | 0.41 | 592 | 86 | 0.430 |  | 0.578 |  |  | 37 | 27 | 49 | 479 | 64 | 0.108 | 1.730 |  |  |  | 57 |  |
| Threshold sweep | Internal | Rapid Prog. | 0.145 | XGBoost | - | 0.42 | 592 | 86 | 0.430 |  | 0.597 |  |  | 37 | 25 | 49 | 481 | 62 | 0.105 | 1.676 |  |  |  | 57 |  |
| Threshold sweep | Internal | Rapid Prog. | 0.145 | XGBoost | - | 0.43 | 592 | 86 | 0.419 |  | 0.632 |  |  | 36 | 21 | 50 | 485 | 57 | 0.096 | 1.583 |  |  |  | 58.1 |  |
| Threshold sweep | Internal | Rapid Prog. | 0.145 | XGBoost | - | 0.44 | 592 | 86 | 0.407 |  | 0.636 |  |  | 35 | 20 | 51 | 486 | 55 | 0.093 | 1.571 |  |  |  | 59.3 |  |
| Threshold sweep | Internal | Rapid Prog. | 0.145 | XGBoost | - | 0.45 | 592 | 86 | 0.395 |  | 0.642 |  |  | 34 | 19 | 52 | 487 | 53 | 0.090 | 1.559 |  |  |  | 60.5 |  |
| Threshold sweep | Internal | Rapid Prog. | 0.145 | XGBoost | - | 0.46 | 592 | 86 | 0.395 |  | 0.642 |  |  | 34 | 19 | 52 | 487 | 53 | 0.090 | 1.559 |  |  |  | 60.5 |  |
| Threshold sweep | Internal | Rapid Prog. | 0.145 | XGBoost | - | 0.47 | 592 | 86 | 0.395 |  | 0.642 |  |  | 34 | 19 | 52 | 487 | 53 | 0.090 | 1.559 |  |  |  | 60.5 |  |
| Threshold sweep | Internal | Rapid Prog. | 0.145 | XGBoost | - | 0.48 | 592 | 86 | 0.372 |  | 0.64 |  |  | 32 | 18 | 54 | 488 | 50 | 0.084 | 1.5625 |  |  |  | 62.8 |  |
| Threshold sweep | Internal | Rapid Prog. | 0.145 | XGBoost | - | 0.49 | 592 | 86 | 0.372 |  | 0.667 |  |  | 32 | 16 | 54 | 490 | 48 | 0.081 | 1.5 |  |  |  | 62.8 |  |
| Threshold sweep | Internal | Rapid Prog. | 0.145 | XGBoost | - | 0.5 | 592 | 86 | 0.372 |  | 0.711 |  |  | 32 | 13 | 54 | 493 | 45 | 0.076 | 1.406 |  |  |  | 62.8 |  |
| Threshold sweep | Internal | Rapid Prog. | 0.145 | XGBoost | - | 0.51 | 592 | 86 | 0.360 |  | 0.738 |  |  | 31 | 11 | 55 | 495 | 42 | 0.071 | 1.355 |  |  |  | 64 |  |
| Threshold sweep | Internal | Rapid Prog. | 0.145 | XGBoost | - | 0.52 | 592 | 86 | 0.349 |  | 0.732 |  |  | 30 | 11 | 56 | 495 | 41 | 0.069 | 1.367 |  |  |  | 65.1 |  |
| Threshold sweep | Internal | Rapid Prog. | 0.145 | XGBoost | - | 0.53 | 592 | 86 | 0.326 |  | 0.718 |  |  | 28 | 11 | 58 | 495 | 39 | 0.066 | 1.393 |  |  |  | 67.4 |  |
| Threshold sweep | Internal | Rapid Prog. | 0.145 | XGBoost | - | 0.54 | 592 | 86 | 0.326 |  | 0.718 |  |  | 28 | 11 | 58 | 495 | 39 | 0.066 | 1.393 |  |  |  | 67.4 |  |
| Threshold sweep | Internal | Rapid Prog. | 0.145 | XGBoost | - | 0.55 | 592 | 86 | 0.314 |  | 0.75 |  |  | 27 | 9 | 59 | 497 | 36 | 0.061 | 1.333 |  |  |  | 68.6 |  |
| Threshold sweep | Internal | Rapid Prog. | 0.145 | XGBoost | - | 0.56 | 592 | 86 | 0.314 |  | 0.75 |  |  | 27 | 9 | 59 | 497 | 36 | 0.061 | 1.333 |  |  |  | 68.6 |  |
| Threshold sweep | Internal | Rapid Prog. | 0.145 | XGBoost | - | 0.57 | 592 | 86 | 0.314 |  | 0.75 |  |  | 27 | 9 | 59 | 497 | 36 | 0.061 | 1.333 |  |  |  | 68.6 |  |
| Threshold sweep | Internal | Rapid Prog. | 0.145 | XGBoost | - | 0.58 | 592 | 86 | 0.302 |  | 0.743 |  |  | 26 | 9 | 60 | 497 | 35 | 0.059 | 1.346 |  |  |  | 69.8 |  |
| Threshold sweep | Internal | Rapid Prog. | 0.145 | XGBoost | - | 0.59 | 592 | 86 | 0.279 |  | 0.75 |  |  | 24 | 8 | 62 | 498 | 32 | 0.054 | 1.333 |  |  |  | 72.1 |  |
| Threshold sweep | Internal | Rapid Prog. | 0.145 | XGBoost | - | 0.6 | 592 | 86 | 0.267 |  | 0.767 |  |  | 23 | 7 | 63 | 499 | 30 | 0.051 | 1.304 |  |  |  | 73.3 |  |
| Threshold sweep | Internal | Rapid Prog. | 0.145 | XGBoost | - | 0.61 | 592 | 86 | 0.233 |  | 0.741 |  |  | 20 | 7 | 66 | 499 | 27 | 0.046 | 1.35 |  |  |  | 76.7 |  |
| Threshold sweep | Internal | Rapid Prog. | 0.145 | XGBoost | - | 0.62 | 592 | 86 | 0.233 |  | 0.769 |  |  | 20 | 6 | 66 | 500 | 26 | 0.044 | 1.3 |  |  |  | 76.7 |  |
| Threshold sweep | Internal | Rapid Prog. | 0.145 | XGBoost | - | 0.63 | 592 | 86 | 0.233 |  | 0.769 |  |  | 20 | 6 | 66 | 500 | 26 | 0.044 | 1.3 |  |  |  | 76.7 |  |
| Threshold sweep | Internal | Rapid Prog. | 0.145 | XGBoost | - | 0.64 | 592 | 86 | 0.209 |  | 0.75 |  |  | 18 | 6 | 68 | 500 | 24 | 0.041 | 1.333 |  |  |  | 79.1 |  |
| Threshold sweep | Internal | Rapid Prog. | 0.145 | XGBoost | - | 0.65 | 592 | 86 | 0.186 |  | 0.762 |  |  | 16 | 5 | 70 | 501 | 21 | 0.035 | 1.3125 |  |  |  | 81.4 |  |
| Threshold sweep | Internal | Rapid Prog. | 0.145 | XGBoost | - | 0.66 | 592 | 86 | 0.174 |  | 0.789 |  |  | 15 | 4 | 71 | 502 | 19 | 0.032 | 1.267 |  |  |  | 82.6 |  |
| Threshold sweep | Internal | Rapid Prog. | 0.145 | XGBoost | - | 0.67 | 592 | 86 | 0.174 |  | 0.789 |  |  | 15 | 4 | 71 | 502 | 19 | 0.032 | 1.267 |  |  |  | 82.6 |  |
| Threshold sweep | Internal | Rapid Prog. | 0.145 | XGBoost | - | 0.68 | 592 | 86 | 0.140 |  | 0.75 |  |  | 12 | 4 | 74 | 502 | 16 | 0.027 | 1.333 |  |  |  | 86 |  |
| Threshold sweep | Internal | Rapid Prog. | 0.145 | XGBoost | - | 0.69 | 592 | 86 | 0.105 |  | 0.692 |  |  | 9 | 4 | 77 | 502 | 13 | 0.022 | 1.444 |  |  |  | 89.5 |  |
| Threshold sweep | Internal | Rapid Prog. | 0.145 | XGBoost | - | 0.7 | 592 | 86 | 0.081 |  | 0.636 |  |  | 7 | 4 | 79 | 502 | 11 | 0.019 | 1.571 |  |  |  | 91.9 |  |
| Threshold sweep | Internal | Rapid Prog. | 0.145 | XGBoost | - | 0.71 | 592 | 86 | 0.070 |  | 0.6 |  |  | 6 | 4 | 80 | 502 | 10 | 0.017 | 1.667 |  |  |  | 93 |  |
| Threshold sweep | Internal | Rapid Prog. | 0.145 | XGBoost | - | 0.72 | 592 | 86 | 0.070 |  | 0.6 |  |  | 6 | 4 | 80 | 502 | 10 | 0.017 | 1.667 |  |  |  | 93 |  |
| Threshold sweep | Internal | Rapid Prog. | 0.145 | XGBoost | - | 0.73 | 592 | 86 | 0.047 |  | 0.571 |  |  | 4 | 3 | 82 | 503 | 7 | 0.012 | 1.75 |  |  |  | 95.3 |  |
| Threshold sweep | Internal | Rapid Prog. | 0.145 | XGBoost | - | 0.74 | 592 | 86 | 0.047 |  | 0.571 |  |  | 4 | 3 | 82 | 503 | 7 | 0.012 | 1.75 |  |  |  | 95.3 |  |
| Threshold sweep | Internal | Rapid Prog. | 0.145 | XGBoost | - | 0.75 | 592 | 86 | 0.047 |  | 0.8 |  |  | 4 | 1 | 82 | 505 | 5 | 0.008 | 1.25 |  |  |  | 95.3 |  |
| Threshold sweep | Internal | Rapid Prog. | 0.145 | XGBoost | - | 0.76 | 592 | 86 | 0.047 |  | 0.8 |  |  | 4 | 1 | 82 | 505 | 5 | 0.008 | 1.25 |  |  |  | 95.3 |  |
| Threshold sweep | Internal | Rapid Prog. | 0.145 | XGBoost | - | 0.77 | 592 | 86 | 0.035 |  | 0.75 |  |  | 3 | 1 | 83 | 505 | 4 | 0.007 | 1.333 |  |  |  | 96.5 |  |
| Threshold sweep | Internal | Rapid Prog. | 0.145 | XGBoost | - | 0.78 | 592 | 86 | 0.035 |  | 0.75 |  |  | 3 | 1 | 83 | 505 | 4 | 0.007 | 1.333 |  |  |  | 96.5 |  |
| Threshold sweep | Internal | Rapid Prog. | 0.145 | XGBoost | - | 0.79 | 592 | 86 | 0.035 |  | 0.75 |  |  | 3 | 1 | 83 | 505 | 4 | 0.007 | 1.333 |  |  |  | 96.5 |  |
| Threshold sweep | Internal | Rapid Prog. | 0.145 | XGBoost | - | 0.8 | 592 | 86 | 0.035 |  | 0.75 |  |  | 3 | 1 | 83 | 505 | 4 | 0.007 | 1.333 |  |  |  | 96.5 |  |
| Threshold sweep | Internal | Rapid Prog. | 0.145 | XGBoost | - | 0.81 | 592 | 86 | 0.035 |  | 0.75 |  |  | 3 | 1 | 83 | 505 | 4 | 0.007 | 1.333 |  |  |  | 96.5 |  |
| Threshold sweep | Internal | Rapid Prog. | 0.145 | XGBoost | - | 0.82 | 592 | 86 | 0.035 |  | 1 |  |  | 3 | 0 | 83 | 506 | 3 | 0.005 | 1 |  |  |  | 96.5 |  |
| Threshold sweep | Internal | Rapid Prog. | 0.145 | XGBoost | - | 0.83 | 592 | 86 | 0.035 |  | 1 |  |  | 3 | 0 | 83 | 506 | 3 | 0.005 | 1 |  |  |  | 96.5 |  |
| Threshold sweep | Internal | Rapid Prog. | 0.145 | XGBoost | - | 0.84 | 592 | 86 | 0.023 |  | 1 |  |  | 2 | 0 | 84 | 506 | 2 | 0.003 | 1 |  |  |  | 97.7 |  |
| Threshold sweep | Internal | Rapid Prog. | 0.145 | XGBoost | - | 0.85 | 592 | 86 | 0.023 |  | 1 |  |  | 2 | 0 | 84 | 506 | 2 | 0.003 | 1 |  |  |  | 97.7 |  |
| Threshold sweep | Internal | Rapid Prog. | 0.145 | XGBoost | - | 0.86 | 592 | 86 | 0.023 |  | 1 |  |  | 2 | 0 | 84 | 506 | 2 | 0.003 | 1 |  |  |  | 97.7 |  |
| Threshold sweep | Internal | Rapid Prog. | 0.145 | XGBoost | - | 0.87 | 592 | 86 | 0.012 |  | 1 |  |  | 1 | 0 | 85 | 506 | 1 | 0.002 | 1 |  |  |  | 98.8 |  |
| Threshold sweep | Internal | Rapid Prog. | 0.145 | XGBoost | - | 0.88 | 592 | 86 | 0 |  | NA |  |  | 0 | 0 | 86 | 506 | 0 | 0 | NA |  |  |  | 100 |  |
| Threshold sweep | Internal | Rapid Prog. | 0.145 | XGBoost | - | 0.89 | 592 | 86 | 0 |  | NA |  |  | 0 | 0 | 86 | 506 | 0 | 0 | NA |  |  |  | 100 |  |
| Threshold sweep | Internal | Rapid Prog. | 0.145 | XGBoost | - | 0.9 | 592 | 86 | 0 |  | NA |  |  | 0 | 0 | 86 | 506 | 0 | 0 | NA |  |  |  | 100 |  |
| Threshold sweep | Internal | Rapid Prog. | 0.145 | XGBoost | - | 0.91 | 592 | 86 | 0 |  | NA |  |  | 0 | 0 | 86 | 506 | 0 | 0 | NA |  |  |  | 100 |  |
| Threshold sweep | Internal | Rapid Prog. | 0.145 | XGBoost | - | 0.92 | 592 | 86 | 0 |  | NA |  |  | 0 | 0 | 86 | 506 | 0 | 0 | NA |  |  |  | 100 |  |
| Threshold sweep | Internal | Rapid Prog. | 0.145 | XGBoost | - | 0.93 | 592 | 86 | 0 |  | NA |  |  | 0 | 0 | 86 | 506 | 0 | 0 | NA |  |  |  | 100 |  |
| Threshold sweep | Internal | Rapid Prog. | 0.145 | XGBoost | - | 0.94 | 592 | 86 | 0 |  | NA |  |  | 0 | 0 | 86 | 506 | 0 | 0 | NA |  |  |  | 100 |  |
| Threshold sweep | Internal | Rapid Prog. | 0.145 | XGBoost | - | 0.95 | 592 | 86 | 0 |  | NA |  |  | 0 | 0 | 86 | 506 | 0 | 0 | NA |  |  |  | 100 |  |
| Screening yield | Internal | Rapid Prog. | 0.145 | XGBoost | - | - | 592 | 86 | 0.419 |  |  |  |  | 36 |  |  |  | 59 | 0.10 |  |  |  |  |  | 4.2 |
| Screening yield | Internal | Rapid Prog. | 0.145 | XGBoost | - | - | 592 | 86 | 0.581 |  |  |  |  | 50 |  |  |  | 118 | 0.20 |  |  |  |  |  | 2.92 |
| Screening yield | Internal | Rapid Prog. | 0.145 | XGBoost | - | - | 592 | 86 | 0.721 |  |  |  |  | 62 |  |  |  | 178 | 0.30 |  |  |  |  |  | 2.4 |
| Screening yield | Internal | Rapid Prog. | 0.145 | XGBoost | - | - | 592 | 86 | 0.791 |  |  |  |  | 68 |  |  |  | 237 | 0.40 |  |  |  |  |  | 1.98 |
| Screening yield | Internal | Rapid Prog. | 0.145 | XGBoost | - | - | 592 | 86 | 0.849 |  |  |  |  | 73 |  |  |  | 296 | 0.50 |  |  |  |  |  | 1.7 |

**Notes:** The intended clinical use was pre-specified as the identification of participants at risk of rapid progression for closer follow-up, not the assignment of every participant to one of the four trajectories. The section column identifies three blocks whose unit of observation differs: operating-point rows are indexed by model and operating point, threshold-sweep rows by predicted probability, and landmark rows by the percentage of the cohort screened. Four operating points are reported: the Youden-optimal threshold, the lowest thresholds achieving a sensitivity of at least 0.90 and of at least 0.80, and the threshold equal to the observed prevalence. External rows apply, without re-optimisation, the thresholds selected on the internal testing set for the same model, and carry that model’s internal sensitivity and positive predictive value alongside for comparison; the operating-point labels therefore describe the rule used to choose the threshold internally and not a criterion met externally. All external rows use the parsimonious model, matching the pre-specified primary external analysis, whereas the internal operating-point rows use the full-predictor model; the two blocks are not directly comparable. In the threshold-sweep block only sensitivity and positive predictive value are tabulated; specificity, negative predictive value and F1 are available in the machine-readable supplement. Missed cases are rapid progressors whose predicted probability fell below the threshold (equal to FN); unnecessary follow-up equals FP. The number needed to screen is given as N screened per case.

**Table S11. Top 15 SHAP features by trajectory class and the consensus feature set.**

| **Trajectory class** | **Rank** | **Feature** | **Consensus feature (top 15 in all four classes)** | **Mean absolute SHAP across classes (consensus features)** |
| --- | --- | --- | --- | --- |
| Improvement | 1 | Baseline CKM stage (Wave 1) | Yes | 0.499 |
|  | 2 | Age (years) | Yes | 0.227 |
|  | 3 | Waist circumference (cm) | No |  |
|  | 4 | TyG-BMI index | Yes | 0.049 |
|  | 5 | Fasting plasma glucose (mg/dL) | Yes | 0.058 |
|  | 6 | Body mass index (kg/m2) | No |  |
|  | 7 | LDL cholesterol (mg/dL) | No |  |
|  | 8 | Total cholesterol (mg/dL) | No |  |
|  | 9 | Systolic blood pressure (mmHg) | Yes | 0.043 |
|  | 10 | Left handgrip strength (kg) | No |  |
|  | 11 | Sex | No |  |
|  | 12 | Triglycerides (mg/dL) | Yes |  |
|  | 13 | Global cognitive score (0-21) | No |  |
|  | 14 | Right handgrip strength (kg) | No |  |
|  | 15 | Episodic memory score (0-10) | No |  |
| Stable | 1 | Baseline CKM stage (Wave 1) | Yes | 0.499 |
|  | 2 | Age (years) | Yes | 0.227 |
|  | 3 | Triglycerides (mg/dL) | Yes |  |
|  | 4 | Platelet count (x10^9/L) | No |  |
|  | 5 | Systolic blood pressure (mmHg) | Yes | 0.043 |
|  | 6 | Body weight (kg) | No |  |
|  | 7 | Diastolic blood pressure (mmHg) | No |  |
|  | 8 | Mean corpuscular volume (fL) | No |  |
|  | 9 | Heart rate (bpm) | No |  |
|  | 10 | Waist circumference (cm) | No |  |
|  | 11 | Fasting plasma glucose (mg/dL) | Yes | 0.058 |
|  | 12 | TyG-BMI index | Yes | 0.049 |
|  | 13 | Total cholesterol (mg/dL) | No |  |
|  | 14 | LDL cholesterol (mg/dL) | No |  |
|  | 15 | HDL cholesterol (mg/dL) | No |  |
| Mild Progression | 1 | Baseline CKM stage (Wave 1) | Yes | 0.499 |
|  | 2 | Age (years) | Yes | 0.227 |
|  | 3 | Ever smoked | No |  |
|  | 4 | Fasting plasma glucose (mg/dL) | Yes | 0.058 |
|  | 5 | Triglycerides (mg/dL) | Yes |  |
|  | 6 | Diastolic blood pressure (mmHg) | No |  |
|  | 7 | Current smoking | No |  |
|  | 8 | TyG-BMI index | Yes | 0.049 |
|  | 9 | Waist circumference (cm) | No |  |
|  | 10 | Platelet count (x10^9/L) | No |  |
|  | 11 | LDL cholesterol (mg/dL) | No |  |
|  | 12 | Body weight (kg) | No |  |
|  | 13 | Systolic blood pressure (mmHg) | Yes | 0.043 |
|  | 14 | HDL cholesterol (mg/dL) | No |  |
|  | 15 | Peak expiratory flow (L/min) | No |  |
| Rapid Progression | 1 | Baseline CKM stage (Wave 1) | Yes | 0.499 |
|  | 2 | Age (years) | Yes | 0.227 |
|  | 3 | Self-rated health | No |  |
|  | 4 | Fasting plasma glucose (mg/dL) | Yes | 0.058 |
|  | 5 | Peak expiratory flow (L/min) | No |  |
|  | 6 | Systolic blood pressure (mmHg) | Yes | 0.043 |
|  | 7 | CES-D-10 depressive symptoms score | No |  |
|  | 8 | Platelet count (x10^9/L) | No |  |
|  | 9 | Right handgrip strength (kg) | No |  |
|  | 10 | Hemoglobin (g/dL) | No |  |
|  | 11 | Sleep duration (hours/night) | No |  |
|  | 12 | Episodic memory score (0-10) | No |  |
|  | 13 | Triglycerides (mg/dL) | Yes |  |
|  | 14 | Household registration (Hukou) | No |  |
|  | 15 | TyG-BMI index | Yes | 0.049 |

**Notes**: Features are ranked by mean absolute SHAP value computed with the predcontrib function on all training-set participants. SHAP values were calculated separately for each trajectory class using the one-versus-rest decomposition from the XGBoost multi-class model (multi:softprob objective). A SHAP value is the contribution of a variable to the predicted log-odds of that class relative to the class base value; it describes the behaviour of the fitted model and is not an estimate of a causal effect. The union of the four top-15 sets comprises 29 predictors. The consensus column is derived from these rows: a feature is marked Yes when it appears in the top 15 of all four classes, which identifies six predictors - baseline CKM stage, age, fasting plasma glucose, TyG-BMI index, systolic blood pressure and triglycerides - and these are the six used for the parsimonious model.

**Table S12. Attribution of discrimination to baseline CKM stage on the internal test set (N = 592).**

| **Specification** | **Model** | **N Predictors** | **Macro-AUC** | **Micro-AUC** | **Accuracy** | **Macro F1** | **Brier** | **Gain from other predictors** | **Gain from adding stage** | **Total gain over stage only** |
| --- | --- | --- | --- | --- | --- | --- | --- | --- | --- | --- |
| Baseline CKM stage only | Conditional class frequencies | 1 | 0.689 | 0.820 | 0.649 | 0.444 | 0.527 |  |  |  |
| All predictors EXCEPT baseline stage | Multinomial LR | 59 | 0.660 | 0.647 | 0.358 | 0.316 | 0.716 |  |  |  |
|  | RF | 59 | 0.700 | 0.782 | 0.515 | 0.392 | 0.630 |  |  |  |
|  | XGBoost | 59 | 0.701 | 0.743 | 0.466 | 0.394 | 0.651 |  |  |  |
|  | GBM | 59 | 0.684 | 0.720 | 0.444 | 0.368 | 0.678 |  |  |  |
|  | SVM | 59 | 0.657 | 0.792 | 0.557 | 0.224 | 0.586 |  |  |  |
|  | Stacking | 59 | 0.697 | 0.676 | 0.367 | 0.356 | 0.708 |  |  |  |
| Full model (including baseline stage) | Multinomial LR | 60 | 0.768 | 0.749 | 0.466 | 0.443 | 0.656 | -0.029 | 0.108 | 0.079 |
|  | RF | 60 | 0.771 | 0.833 | 0.627 | 0.516 | 0.566 | 0.011 | 0.071 | 0.082 |
|  | XGBoost | 60 | 0.786 | 0.805 | 0.542 | 0.480 | 0.593 | 0.012 | 0.085 | 0.097 |
|  | GBM | 60 | 0.777 | 0.800 | 0.541 | 0.481 | 0.597 | -0.005 | 0.093 | 0.088 |
|  | SVM | 60 | 0.750 | 0.842 | 0.603 | 0.321 | 0.524 | -0.032 | 0.093 | 0.061 |
|  | Stacking | 60 | 0.780 | 0.751 | 0.463 | 0.444 | 0.678 | 0.008 | 0.083 | 0.091 |

**Notes:** Three specifications are compared. The stage-only benchmark assigns each participant the conditional class frequencies observed for their baseline CKM stage in the training set, and therefore uses no other information. The stage-free models are refitted after removing baseline CKM stage from the predictor set. The full models are those reported in Table 1. All models use inverse-frequency class weights. Macro-AUC is the unweighted mean of the four one-versus-rest AUCs. gains are differences in Macro-AUC and are shown on the full-model rows only. Gain from other predictors is the stage-free model minus the stage-only benchmark; gain from adding stage is the full model minus the stage-free model.

**Table S13. Outcome classes modelled and discrimination within each baseline CKM stage stratum.**

| **Baseline stage** | **N train** | **N test** | **Classes modelled** | **N classes** | **Classes dropped (reason)** | **Model** | **Macro-AUC** | **Accuracy** | **Macro F1** | **Brier** |
| --- | --- | --- | --- | --- | --- | --- | --- | --- | --- | --- |
| 0 | 298 | 60 | Stable, MildProg, RapidProg | 3 | Improvement (n_train = 0, n_test = 0) | Multinomial LR | 0.596 | 0.550 | 0.422 | 0.626 |
|  |  |  |  |  |  | RF | 0.486 | 0.483 | 0.303 | 0.624 |
|  |  |  |  |  |  | XGBoost | 0.493 | 0.500 | 0.309 | 0.705 |
|  |  |  |  |  |  | GBM | 0.466 | 0.517 | 0.330 | 0.718 |
|  |  |  |  |  |  | SVM | 0.604 | 0.633 | 0.383 | 0.528 |
|  |  |  |  |  |  | Stacking | 0.529 | 0.483 | 0.331 | 0.658 |
| 1 | 419 | 105 | Improvement, Stable, MildProg, RapidProg | 4 |  | Multinomial LR | 0.597 | 0.362 | 0.307 | 0.724 |
|  |  |  |  |  |  | RF | 0.583 | 0.514 | 0.367 | 0.642 |
|  |  |  |  |  |  | XGBoost | 0.569 | 0.486 | 0.347 | 0.717 |
|  |  |  |  |  |  | GBM | 0.565 | 0.448 | 0.341 | 0.718 |
|  |  |  |  |  |  | SVM | 0.583 | 0.448 | 0.264 | 0.630 |
|  |  |  |  |  |  | Stacking | 0.548 | 0.362 | 0.278 | 0.712 |
| 2 | 1446 | 370 | Improvement, Stable, MildProg, RapidProg | 4 |  | Multinomial LR | 0.728 | 0.376 | 0.355 | 0.688 |
|  |  |  |  |  |  | RF | 0.718 | 0.654 | 0.367 | 0.547 |
|  |  |  |  |  |  | XGBoost | 0.718 | 0.622 | 0.363 | 0.513 |
|  |  |  |  |  |  | GBM | 0.701 | 0.505 | 0.392 | 0.615 |
|  |  |  |  |  |  | SVM | 0.723 | 0.692 | 0.276 | 0.453 |
|  |  |  |  |  |  | Stacking | 0.705 | 0.459 | 0.365 | 0.693 |
| 3 | 216 | 57 | Improvement, Stable, MildProg | 3 | RapidProg (n_train = 0, n_test = 0) | Multinomial LR | 0.687 | 0.526 | 0.434 | 0.589 |
|  |  |  |  |  |  | RF | 0.814 | 0.719 | 0.497 | 0.436 |
|  |  |  |  |  |  | XGBoost | 0.773 | 0.684 | 0.472 | 0.385 |
|  |  |  |  |  |  | GBM | 0.788 | 0.684 | 0.519 | 0.417 |
|  |  |  |  |  |  | SVM | 0.557 | 0.702 | 0.275 | 0.455 |
|  |  |  |  |  |  | Stacking | 0.742 | 0.649 | 0.524 | 0.579 |

**Notes:** Because the outcome is defined as the change in CKM stage between waves, some classes cannot occur within a given baseline stratum. Improvement is impossible from stage 0, as no lower stage exists. Rapid progression, defined as an increase of two or more stages, is impossible from stage 3, as stage 4 is the ceiling of the staging system and a two-stage increase would require a stage that does not exist. Mild progression from stage 3 to stage 4 remains possible and was modelled. A class was retained for modelling only if it had at least 10 training observations and at least one test observation, so that every cross-validation fold retained the class and a per-class AUC was estimable. Participants whose observed class was not retained were excluded from that stratum.

**Table S14. Class-specific discrimination within each baseline CKM stage stratum, XGBoost model.**

| **Baseline stage** | **Trajectory class** | **N** | **AUC** | **Sensitivity** | **Specificity** | **PPV** | **NPV** |
| --- | --- | --- | --- | --- | --- | --- | --- |
| 0 | Stable | 5 | 0.484 | 0.000 | 0.964 | 0.000 | 0.914 |
|  | Mild Progression | 15 | 0.479 | 0.333 | 0.644 | 0.238 | 0.744 |
|  | Rapid Progression | 40 | 0.516 | 0.625 | 0.400 | 0.676 | 0.348 |
| 1 | Improvement | 5 | 0.478 | 0.000 | 1.000 |  | 0.952 |
|  | Stable | 34 | 0.552 | 0.412 | 0.676 | 0.378 | 0.706 |
|  | Mild Progression | 54 | 0.542 | 0.611 | 0.451 | 0.541 | 0.523 |
|  | Rapid Progression | 12 | 0.703 | 0.333 | 0.968 | 0.571 | 0.918 |
| 2 | Improvement | 31 | 0.753 | 0.258 | 0.906 | 0.200 | 0.930 |
|  | Stable | 250 | 0.663 | 0.788 | 0.400 | 0.732 | 0.475 |
|  | Mild Progression | 55 | 0.829 | 0.455 | 0.914 | 0.481 | 0.906 |
|  | Rapid Progression | 34 | 0.628 | 0.000 | 0.973 | 0.000 | 0.906 |
| 3 | Improvement | 11 | 0.866 | 0.364 | 0.935 | 0.571 | 0.860 |
|  | Stable | 40 | 0.813 | 0.850 | 0.529 | 0.810 | 0.600 |
|  | Mild Progression | 6 | 0.641 | 0.167 | 0.863 | 0.125 | 0.898 |

**Notes:** Only the classes retained within each stratum appear, so the number of rows differs between strata by design. A blank PPV indicates that the model predicted the class for no participant in that stratum, leaving the quantity undefined.

**Table S15. Performance of the full-predictor and parsimonious models on the internal test set.**

| **Model** | **Trajectory class** | **N predictors, full** | **N predictors, parsimonious** | **AUC, full** | **AUC, parsimonious** | **Delta AUC (pars - full)** | **P (DeLong)** | **CV Macro-AUC, full** | **CV Macro-AUC, pars** | **Optimism, full** | **Optimism, pars** | **Accuracy, full** | **Accuracy, pars** | **Macro-F1, full** | **Macro-F1, pars** | **Brier, full** | **Brier, pars** |
| --- | --- | --- | --- | --- | --- | --- | --- | --- | --- | --- | --- | --- | --- | --- | --- | --- | --- |
| Multinomial LR | Improvement | 60 | 6 | 0.786 | 0.763 | -0.023 | 0.098 |  |  |  |  |  |  |  |  |  |  |
|  | Stable | 60 | 6 | 0.75 | 0.756 | +0.006 | 0.54 |  |  |  |  |  |  |  |  |  |  |
|  | Mild Progression | 60 | 6 | 0.733 | 0.749 | +0.016 | 0.032 |  |  |  |  |  |  |  |  |  |  |
|  | Rapid Progression | 60 | 6 | 0.803 | 0.799 | -0.004 | 0.722 |  |  |  |  |  |  |  |  |  |  |
|  | All classes (Macro) | 60 | 6 | 0.768 | 0.767 | -0.001 | - | 0.75 | 0.755 | -0.018 | -0.012 | 0.466 | 0.444 | 0.443 | 0.424 | 0.656 | 0.66 |
| RF | Improvement | 60 | 6 | 0.765 | 0.771 | +0.006 | 0.82 |  |  |  |  |  |  |  |  |  |  |
|  | Stable | 60 | 6 | 0.758 | 0.765 | +0.007 | 0.607 |  |  |  |  |  |  |  |  |  |  |
|  | Mild Progression | 60 | 6 | 0.762 | 0.763 | +0.001 | 0.954 |  |  |  |  |  |  |  |  |  |  |
|  | Rapid Progression | 60 | 6 | 0.798 | 0.751 | -0.047 | 0.005 |  |  |  |  |  |  |  |  |  |  |
|  | All classes (Macro) | 60 | 6 | 0.771 | 0.762 | -0.009 | - | 0.769 | 0.765 | -0.002 | 0.002 | 0.627 | 0.566 | 0.516 | 0.503 | 0.566 | 0.588 |
| XGBoost | Improvement | 60 | 6 | 0.797 | 0.774 | -0.023 | 0.24 |  |  |  |  |  |  |  |  |  |  |
|  | Stable | 60 | 6 | 0.757 | 0.75 | -0.007 | 0.493 |  |  |  |  |  |  |  |  |  |  |
|  | Mild Progression | 60 | 6 | 0.769 | 0.767 | -0.002 | 0.801 |  |  |  |  |  |  |  |  |  |  |
|  | Rapid Progression | 60 | 6 | 0.82 | 0.798 | -0.022 | 0.157 |  |  |  |  |  |  |  |  |  |  |
|  | All classes (Macro) | 60 | 6 | 0.786 | 0.772 | -0.014 | - | 0.765 | 0.764 | -0.02 | -0.009 | 0.542 | 0.508 | 0.48 | 0.477 | 0.593 | 0.627 |
| GBM | Improvement | 60 | 6 | 0.797 | 0.774 | -0.023 | 0.192 |  |  |  |  |  |  |  |  |  |  |
|  | Stable | 60 | 6 | 0.749 | 0.759 | +0.010 | 0.379 |  |  |  |  |  |  |  |  |  |  |
|  | Mild Progression | 60 | 6 | 0.758 | 0.765 | +0.007 | 0.544 |  |  |  |  |  |  |  |  |  |  |
|  | Rapid Progression | 60 | 6 | 0.804 | 0.782 | -0.022 | 0.237 |  |  |  |  |  |  |  |  |  |  |
|  | All classes (Macro) | 60 | 6 | 0.777 | 0.77 | -0.007 | - | 0.759 | 0.764 | -0.018 | -0.006 | 0.541 | 0.514 | 0.481 | 0.473 | 0.597 | 0.62 |
| SVM | Improvement | 60 | 6 | 0.735 | 0.76 | +0.025 | 0.418 |  |  |  |  |  |  |  |  |  |  |
|  | Stable | 60 | 6 | 0.739 | 0.771 | +0.032 | 0.029 |  |  |  |  |  |  |  |  |  |  |
|  | Mild Progression | 60 | 6 | 0.732 | 0.77 | +0.038 | 0.025 |  |  |  |  |  |  |  |  |  |  |
|  | Rapid Progression | 60 | 6 | 0.792 | 0.812 | +0.020 | 0.395 |  |  |  |  |  |  |  |  |  |  |
|  | All classes (Macro) | 60 | 6 | 0.75 | 0.778 | 0.029 | - | 0.742 | 0.769 | -0.008 | -0.01 | 0.603 | 0.652 | 0.321 | 0.447 | 0.524 | 0.492 |
| Stacking | Improvement | 60 | 6 | 0.783 | 0.777 | -0.006 | 0.665 |  |  |  |  |  |  |  |  |  |  |
|  | Stable | 60 | 6 | 0.758 | 0.767 | +0.009 | 0.315 |  |  |  |  |  |  |  |  |  |  |
|  | Mild Progression | 60 | 6 | 0.764 | 0.764 | +0.000 | 0.996 |  |  |  |  |  |  |  |  |  |  |
|  | Rapid Progression | 60 | 6 | 0.816 | 0.789 | -0.027 | 0.043 |  |  |  |  |  |  |  |  |  |  |
|  | All classes (Macro) | 60 | 6 | 0.78 | 0.774 | -0.006 | - | 0.766 | 0.766 | -0.014 | -0.009 | 0.463 | 0.471 | 0.444 | 0.453 | 0.678 | 0.674 |

**Notes:** Both model versions were tuned by the same protocol on the same folds. The four class rows of each model give the one-versus-rest AUCs of the two versions and the DeLong test between them; the "All classes" row gives the macro-averaged and model-level results, so the columns from CV Macro-AUC onward are populated on that row only. Optimism is the training-set Macro-AUC minus the cross-validated value at the same sample size; negative values indicate that the test-set value exceeded the cross-validated one. The parsimonious model changed Macro-AUC by between -0.014 and +0.029; for the support vector machine the six-predictor model was the better of the two by 0.029, with overall accuracy rising from 0.603 to 0.652 and Macro-F1 from 0.321 to 0.447. Two of the 24 class-specific DeLong comparisons reached nominal significance: the random forest lost Rapid Progression discrimination (0.798 to 0.751, P = 0.005) and Multinomial LR gained Mild Progression discrimination (0.733 to 0.749, P = 0.032). Because the consensus set was derived from the training folds, the optimism of the parsimonious model is reported alongside that of the full model rather than in place of it.

**Table S16. Development versus external cohort: baseline characteristics, standardised mean differences and external missingness.**

| **Characteristic** | **Development (N = 2,971)** | **External (N = 291)** | **SMD** | **Comparability** | **N missing (external)** | **% missing (external)** |
| --- | --- | --- | --- | --- | --- | --- |
| Sex, male vs female, n (%) | 1,346 (45.3%) | 151 (51.9%) | -0.132 | Modest difference | 0 | 0.0 |
| Marital status, married or partnered vs not partnered, n (%) | 2,718 (91.5%) | 244 (83.8%) | +0.234 | Modest difference | 0 | 0.0 |
| Residence type, rural vs urban, n (%) | 1,955 (65.8%) | 111 (38.1%) | +0.576 | Substantial difference (\|SMD\| >= 0.25) | 0 | 0.0 |
| Household registration (Hukou), agricultural vs non-agricultural, n (%) | 2,501 (84.2%) | 160 (55.0%) | +0.671 | Substantial difference (\|SMD\| >= 0.25) | 0 | 0.0 |
| **Self-rated health, n (%)** | n = 2,970 | Not available |  | Variable absent externally | 291 | 100.0 |
| 1 = very good | 80 (2.7%) | Not available |  | Variable absent externally | 291 | 100.0 |
| 2 = good | 570 (19.2%) | Not available |  | Variable absent externally | 291 | 100.0 |
| 3 = fair | 1,556 (52.4%) | Not available |  | Variable absent externally | 291 | 100.0 |
| 4 = poor | 575 (19.4%) | Not available |  | Variable absent externally | 291 | 100.0 |
| 5 = very poor | 189 (6.4%) | Not available |  | Variable absent externally | 291 | 100.0 |
| ADL disability count, median (IQR); mean ± SD | 0.00 (0.00, 0.00); 0.21 ± 0.66 | Not available |  | Variable absent externally | 291 | 100.0 |
| Cancer, yes vs no, n (%) | 19 (0.6%) | 9 (3.1%) | -0.182 | Modest difference | 0 | 0.0 |
| Lung disease, yes vs no, n (%) | 224 (7.5%) | 41 (14.1%) | -0.212 | Modest difference | 0 | 0.0 |
| Psychiatric disorder, yes vs no, n (%) | 27 (0.9%) | 6 (2.1%) | -0.095 | Similar (\|SMD\| < 0.10) | 0 | 0.0 |
| Arthritis, yes vs no, n (%) | 942 (31.7%) | 70 (24.1%) | +0.172 | Modest difference | 0 | 0.0 |
| Liver disease, yes vs no, n (%) | 81 (2.7%) | 26 (8.9%) | -0.267 | Substantial difference (\|SMD\| >= 0.25) | 0 | 0.0 |
| Digestive disease, yes vs no, n (%) | 584 (19.7%) | 44 (15.1%) | +0.120 | Modest difference | 0 | 0.0 |
| Asthma, yes vs no, n (%) | 105 (3.5%) | 18 (6.2%) | -0.123 | Modest difference | 0 | 0.0 |
| Memory-related disease, yes vs no, n (%) | 24 (0.8%) | 6 (2.1%) | -0.105 | Modest difference | 0 | 0.0 |
| Ever consumed alcohol, ever vs never, n (%) | 1,160 (39.1%) | 113 (44.0%) | -0.099 | Similar (\|SMD\| < 0.10) | 34 | 11.7 |
| Current alcohol use, drank in the past year vs did not, n (%) | 1,021 (34.4%) | 80 (31.1%) | +0.069 | Similar (\|SMD\| < 0.10) | 34 | 11.7 |
| Ever smoked, ever vs never, n (%) | 1,079 (36.3%) | 107 (41.2%) | -0.099 | Similar (\|SMD\| < 0.10) | 31 | 10.7 |
| Current smoking, currently smoking vs not currently, n (%) | 838 (28.3%) | 68 (26.2%) | +0.047 | Similar (\|SMD\| < 0.10) | 31 | 10.7 |
| Total household income (CNY/year), median (IQR) | 12,500.00 (7,645.00, 19,600.00) | Not available |  | Variable absent externally | 291 | 100.0 |
| Retirement status, retired vs not retired, n (%) | 245 (8.4%) | 91 (33.8%) | -0.656 | Substantial difference (\|SMD\| >= 0.25) | 22 | 7.6 |
| Systolic blood pressure (mmHg), median (IQR) | 124.00 (113.00, 138.00) | 137.00 (124.00, 149.00) | -0.575 | Substantial difference (\|SMD\| >= 0.25) | 0 | 0.0 |
| Diastolic blood pressure (mmHg), median (IQR) | 74.00 (67.00, 82.00) | 83.00 (73.50, 93.00) | -0.649 | Substantial difference (\|SMD\| >= 0.25) | 0 | 0.0 |
| Heart rate (bpm), median (IQR) | 72.00 (65.00, 78.00) | 77.00 (69.00, 85.00) | -0.425 | Substantial difference (\|SMD\| >= 0.25) | 0 | 0.0 |
| Left handgrip strength (kg), median (IQR) | 30.00 (25.00, 38.00) | Not available |  | Variable absent externally | 291 | 100.0 |
| Right handgrip strength (kg), median (IQR) | 32.00 (26.00, 40.00) | Not available |  | Variable absent externally | 291 | 100.0 |
| Height (m), median (IQR) | 1.60 (1.53, 1.67) | 1.60 (1.55, 1.65) | +0.000 | Similar (\|SMD\| < 0.10) | 0 | 0.0 |
| Body weight (kg), median (IQR) | 59.00 (53.00, 66.00) | 68.00 (60.00, 76.00) | -0.727 | Substantial difference (\|SMD\| >= 0.25) | 0 | 0.0 |
| Waist circumference (cm), median (IQR) | 86.00 (79.00, 92.00) | 92.00 (85.00, 98.00) | -0.590 | Substantial difference (\|SMD\| >= 0.25) | 0 | 0.0 |
| Body mass index (kg/m2), median (IQR) | 24.00 (22.00, 26.00) | 26.50 (24.40, 29.45) | -0.735 | Substantial difference (\|SMD\| >= 0.25) | 0 | 0.0 |
| Peak expiratory flow (L/min), median (IQR) | 300.00 (220.00, 380.00) | 270.00 (195.00, 340.00) | +0.336 | Substantial difference (\|SMD\| >= 0.25) | 63 | 21.6 |
| CES-D-10 depressive symptoms score, median (IQR) | 7.00 (3.00, 11.00) | Not available |  | Variable absent externally | 291 | 100.0 |
| **Life satisfaction, n (%)** | n = 2,750 | Not available |  | Variable absent externally | 291 | 100.0 |
| 1 = completely satisfied | 54 (2.0%) | Not available |  | Variable absent externally | 291 | 100.0 |
| 2 = very satisfied | 335 (12.2%) | Not available |  | Variable absent externally | 291 | 100.0 |
| 3 = somewhat satisfied | 1,737 (63.2%) | Not available |  | Variable absent externally | 291 | 100.0 |
| 4 = not very satisfied | 571 (20.8%) | Not available |  | Variable absent externally | 291 | 100.0 |
| 5 = not at all satisfied | 53 (1.9%) | Not available |  | Variable absent externally | 291 | 100.0 |
| Sleep duration (hours/night), median (IQR) | 7.00 (5.00, 8.00) | Not available |  | Variable absent externally | 291 | 100.0 |
| White blood cell count (x10^3/uL), median (IQR) | 6.00 (4.92, 7.31) | 6.40 (5.30, 7.80) | -0.235 | Modest difference | 0 | 0.0 |
| Mean corpuscular volume (fL), median (IQR) | 91.00 (87.00, 95.00) | 92.10 (87.50, 96.70) | -0.209 | Modest difference | 0 | 0.0 |
| Platelet count (x10^9/L), median (IQR) | 207.00 (165.00, 255.00) | 189.00 (153.00, 234.00) | +0.214 | Modest difference | 0 | 0.0 |
| Blood urea nitrogen (mg/dL), median (IQR) | 15.00 (12.00, 18.00) | 16.20 (13.10, 19.90) | -0.325 | Substantial difference (\|SMD\| >= 0.25) | 0 | 0.0 |
| Fasting plasma glucose (mg/dL), median (IQR) | 102.00 (95.00, 112.00) | 121.00 (103.50, 133.50) | -0.391 | Substantial difference (\|SMD\| >= 0.25) | 0 | 0.0 |
| Serum creatinine (mg/dL), median (IQR) | 0.77 (0.64, 0.93) | 1.01 (0.73, 1.27) | -0.695 | Substantial difference (\|SMD\| >= 0.25) | 0 | 0.0 |
| Total cholesterol (mg/dL), median (IQR) | 193.00 (171.00, 217.00) | 205.00 (180.00, 235.50) | -0.321 | Substantial difference (\|SMD\| >= 0.25) | 0 | 0.0 |
| Triglycerides (mg/dL), median (IQR) | 104.00 (74.50, 151.50) | 155.00 (110.00, 238.50) | -0.549 | Substantial difference (\|SMD\| >= 0.25) | 0 | 0.0 |
| HDL cholesterol (mg/dL), median (IQR) | 49.00 (41.00, 60.00) | 47.00 (40.00, 55.50) | +0.272 | Substantial difference (\|SMD\| >= 0.25) | 0 | 0.0 |
| LDL cholesterol (mg/dL), median (IQR) | 117.00 (98.00, 140.00) | 126.00 (103.00, 145.50) | -0.131 | Modest difference | 0 | 0.0 |
| C-reactive protein (mg/L), median (IQR) | 0.82 (0.31, 2.21) | 1.83 (1.16, 2.97) | -0.017 | Similar (\|SMD\| < 0.10) | 28 | 9.6 |
| Glycated hemoglobin HbA1c (%), median (IQR) | 5.23 (4.70, 5.83) | 5.95 (5.40, 6.32) | -0.790 | Substantial difference (\|SMD\| >= 0.25) | 23 | 7.9 |
| Uric acid (mg/dL), median (IQR) | 4.23 (3.52, 5.08) | 5.20 (4.40, 6.30) | -0.759 | Substantial difference (\|SMD\| >= 0.25) | 19 | 6.5 |
| Hematocrit (%), median (IQR) | 42.00 (38.00, 45.00) | 45.90 (42.20, 49.50) | -0.693 | Substantial difference (\|SMD\| >= 0.25) | 0 | 0.0 |
| Hemoglobin (g/dL), median (IQR) | 14.00 (13.00, 16.00) | 15.90 (14.60, 17.20) | -0.612 | Substantial difference (\|SMD\| >= 0.25) | 0 | 0.0 |
| Cystatin C (mg/L), median (IQR) | 1.00 (0.90, 1.11) | 1.15 (0.97, 1.37) | -0.730 | Substantial difference (\|SMD\| >= 0.25) | 47 | 16.2 |
| Age (years), median (IQR) | 57.00 (51.00, 63.00) | 65.10 (59.50, 70.40) | -0.925 | Substantial difference (\|SMD\| >= 0.25) | 0 | 0.0 |
| **Education level, n (%)** | 2,970 | 265 | 0.208 | Modest difference | 26 | 8.9 |
| 1 = illiterate or no formal schooling | 1,315 (44.3%) | 101 (38.1%) | +0.125 | Modest difference | 26 | 8.9 |
| 2 = primary school | 695 (23.4%) | 53 (20.0%) | +0.083 | Similar (\|SMD\| < 0.10) | 26 | 8.9 |
| 3 = middle school | 656 (22.1%) | 66 (24.9%) | -0.067 | Similar (\|SMD\| < 0.10) | 26 | 8.9 |
| 4 = high school or above | 304 (10.2%) | 45 (17.0%) | -0.198 | Modest difference | 26 | 8.9 |
| Physical exercise, yes vs no, n (%) | 2,871 (96.6%) | Not available |  | Variable absent externally | 291 | 100.0 |
| Total physical activity (MET-min/week), median (IQR) | 6,530.00 (1,732.20, 13,433.83) | Not available |  | Variable absent externally | 291 | 100.0 |
| Episodic memory score (0-10), median (IQR) | 4.00 (3.00, 5.00) | Not available |  | Variable absent externally | 291 | 100.0 |
| Executive function score (0-11), median (IQR) | 9.00 (7.00, 10.00) | Not available |  | Variable absent externally | 291 | 100.0 |
| Global cognitive score (0-21), median (IQR) | 13.00 (11.00, 15.00) | Not available |  | Variable absent externally | 291 | 100.0 |
| IADL disability count, median (IQR); mean ± SD | 0.00 (0.00, 0.00); 0.30 ± 0.79 | Not available |  | Variable absent externally | 291 | 100.0 |
| Triglyceride-glucose index (TyG), median (IQR) | 8.58 (8.17, 9.05) | 9.14 (8.68, 9.64) | -0.694 | Substantial difference (\|SMD\| >= 0.25) | 0 | 0.0 |
| TyG-BMI index, median (IQR) | 203.00 (182.00, 230.00) | 244.30 (211.95, 281.35) | -0.848 | Substantial difference (\|SMD\| >= 0.25) | 0 | 0.0 |
| **Baseline CKM stage (Wave 1), n (%)** | 2,971 | 291 | 0.482 | Substantial difference (\|SMD\| >= 0.25) | 0 | 0.0 |
| 0 = stage 0 | 358 (12.0%) | 28 (9.6%) | +0.078 | Similar (\|SMD\| < 0.10) | 0 | 0.0 |
| 1 = stage 1 | 524 (17.6%) | 54 (18.6%) | -0.024 | Similar (\|SMD\| < 0.10) | 0 | 0.0 |
| 2 = stage 2 | 1,816 (61.1%) | 132 (45.4%) | +0.320 | Substantial difference (\|SMD\| >= 0.25) | 0 | 0.0 |
| 3 = stage 3 | 273 (9.2%) | 77 (26.5%) | -0.463 | Substantial difference (\|SMD\| >= 0.25) | 0 | 0.0 |

**Notes:** Continuous variables are summarised as median (interquartile range); categorical variables as n (%). SMD is defined as (m_1_ − m_2_) / sqrt((s_1_² + s_2_²) / 2). For categorical variables the SMD is computed from the proportions underlying the counts shown here; for continuous variables it is computed from the means and standard deviations of the two cohorts, which are not displayed, so for right-skewed variables the median (IQR) shown will not reproduce the SMD arithmetically. Comparability is graded as Similar (|SMD| < 0.10), Modest difference (0.10 ≤ |SMD| < 0.25) or Substantial difference (|SMD| ≥ 0.25). These are descriptive thresholds; no hypothesis test is implied. Of the 60 candidate variables, 46 were measurable in the external cohort; the remaining 14 are not recorded in the external administrative records and are reported as Not available with 291 (100.0%) missing and no SMD.

**Table S17. Composition of the external validation cohort: assessments, follow-up interval, predictor availability and interval-restricted performance.**

| **Domain** | **Item** | **Value** |
| --- | --- | --- |
| Assessments per patient | 2 assessments | 152 patients |
|  | 3 assessments | 139 patients |
| Follow-up interval (years) | N | 291 |
|  | Mean | 2.26 |
|  | SD | 0.82 |
|  | Median | 1.96 |
|  | Q1 | 1.52 |
|  | Q3 | 3.01 |
|  | Min | 1.01 |
|  | Max | 3.92 |
|  | Development interval | 4 years (fixed, Wave 1 2011 to Wave 3 2015) |
|  | External participants reaching the 4-year development interval | 0 |
| Trajectory label | Recomputed from recorded stages using the development rule | 291 (label supplied in the extract not used) |
| Predictor availability | Full model | 46 of 60 predictors measured externally (76.7%) |
|  | Parsimonious (consensus) model | 5 of 6 predictors measured externally (83.3%) |
|  | Predictors not measured externally | 14: self-reported health, functional, psychosocial, socioeconomic, physical-activity, sleep, handgrip and cognitive measures |
| Interval-restricted performance (XGBoost, parsimonious model) | All external participants | n = 291; Macro-AUC 0.742, accuracy 0.467, macro F1 0.419 - primary external analysis |
|  | 3.0-5.0 years | n = 76; Macro-AUC 0.770, accuracy 0.513, macro F1 0.454 |
|  | 3.5-4.5 years | n = 20 - not evaluated: fewer than 50 participants or <2 classes |
|  | 2.0-6.0 years | n = 140; Macro-AUC 0.767, accuracy 0.479, macro F1 0.436 |

**Notes:** Trajectories were recomputed from the recorded stages using the identical rule as the development cohort rather than taken from any label supplied in the extract. Forty-six of the 60 full-model predictors (76.7%) and 5 of the 6 parsimonious predictors (83.3%) were measured in the hospital records; the fourteen absent predictors are the self-reported health, functional, psychosocial, socioeconomic, physical-activity, sleep, handgrip and cognitive measures, none of which forms part of routine hospital documentation. The interval-restricted rows refer to the parsimonious XGBoost model, matching the primary external analysis.

**Table S18. External validation performance of the full-predictor and parsimonious models (N = 291).**

| **Model** | **Model version** | **N predictors** | **Measured externally** | **Macro-AUC (95% CI)** | **AUC: Improvement** | **AUC: Stable** | **AUC: Mild Progression** | **AUC: Rapid Progression** |
| --- | --- | --- | --- | --- | --- | --- | --- | --- |
| Stacking | Full | 60 | 46 | 0.738 (0.693–0.777) | 0.721 | 0.768 | 0.655 | 0.808 |
| XGBoost |  |  |  | 0.725 (0.682–0.769) | 0.703 | 0.759 | 0.644 | 0.793 |
| GBM |  |  |  | 0.702 (0.660–0.746) | 0.661 | 0.749 | 0.628 | 0.771 |
| RF |  |  |  | 0.727 (0.679–0.771) | 0.692 | 0.779 | 0.651 | 0.786 |
| SVM |  |  |  | 0.672 (0.635–0.712) | 0.574 | 0.714 | 0.590 | 0.811 |
| Multinomial LR |  |  |  | 0.714 (0.677–0.756) | 0.682 | 0.728 | 0.619 | 0.829 |
| Stacking | Parsimonious | 6 | 5 | 0.751 (0.707-0.790) | 0.746 | 0.781 | 0.676 | 0.802 |
| XGBoost |  |  |  | 0.742 (0.700-0.783) | 0.746 | 0.789 | 0.636 | 0.798 |
| GBM |  |  |  | 0.735 (0.692-0.778) | 0.728 | 0.78 | 0.654 | 0.78 |
| RF |  |  |  | 0.752 (0.707-0.793) | 0.765 | 0.785 | 0.67 | 0.787 |
| SVM |  |  |  | 0.728 (0.684-0.773) | 0.721 | 0.753 | 0.639 | 0.8 |
| Multinomial LR |  |  |  | 0.713 (0.670-0.755) | 0.693 | 0.733 | 0.63 | 0.795 |

**Notes:** Per-class AUC is one-versus-rest; Macro-AUC is their unweighted mean, reported with 95% bootstrap confidence intervals (1,000 resamples). Both model versions were applied to the external cohort without retraining or recalibration, using preprocessing parameters frozen from the CHARLS training set. The parsimonious model is the primary external analysis, because the external cohort measures 46 of the 60 full-model predictors; in the full-predictor rows the remaining 14 take the development imputation value, so those rows are reported as a sensitivity analysis.

**Table S19. Sensitivity analyses for missing-data handling and outcome definition.**

| **Analysis** | **N train** | **N test** | **Macro-AUC** | **Micro-AUC** | **Overall accuracy** | **Macro-F1** | **Brier** | **Note** |
| --- | --- | --- | --- | --- | --- | --- | --- | --- |
| Main analysis (median/mode imputation, 4 classes) | 2379 | 592 | 0.786 | 0.805 | 0.542 | 0.48 | 0.593 | Reference for all comparisons below |
| S1 Complete-case analysis | 361 | 79 | 0.67 | 0.769 | 0.557 | 0.381 | 0.609 | 14.8% of the cohort had no missing predictor |
| S2 Multiple imputation (m = 10, Rubin-pooled mean) | 2379 | 592 | 0.784 | 0.807 | 0.546 | 0.483 | 0.592 | Macro-AUC range across imputations 0.776-0.789 |
| S2, imputation 1 | 2379 | 592 | 0.787 | 0.811 | 0.547 | 0.484 | 0.585 | Single imputed dataset contributing to the pooled row above |
| S2, imputation 2 | 2379 | 592 | 0.784 | 0.808 | 0.544 | 0.481 | 0.591 | Single imputed dataset contributing to the pooled row above |
| S2, imputation 3 | 2379 | 592 | 0.788 | 0.809 | 0.551 | 0.485 | 0.590 | Single imputed dataset contributing to the pooled row above |
| S2, imputation 4 | 2379 | 592 | 0.788 | 0.809 | 0.542 | 0.479 | 0.589 | Single imputed dataset contributing to the pooled row above |
| S2, imputation 5 | 2379 | 592 | 0.784 | 0.808 | 0.549 | 0.488 | 0.590 | Single imputed dataset contributing to the pooled row above |
| S2, imputation 6 | 2379 | 592 | 0.779 | 0.803 | 0.541 | 0.475 | 0.597 | Single imputed dataset contributing to the pooled row above |
| S2, imputation 7 | 2379 | 592 | 0.776 | 0.801 | 0.542 | 0.479 | 0.600 | Single imputed dataset contributing to the pooled row above |
| S2, imputation 8 | 2379 | 592 | 0.789 | 0.811 | 0.5625 | 0.498 | 0.588 | Single imputed dataset contributing to the pooled row above |
| S2, imputation 9 | 2379 | 592 | 0.785 | 0.808 | 0.546 | 0.489 | 0.591 | Single imputed dataset contributing to the pooled row above |
| S2, imputation 10 | 2379 | 592 | 0.779 | 0.804 | 0.535 | 0.474 | 0.595 | Single imputed dataset contributing to the pooled row above |
| S3 Collapsed 3-class outcome (any progression) | 2379 | 592 | 0.783 | 0.821 | 0.63 | 0.546 | 0.48 | Averaged over 3 classes; not directly comparable with the 4-class rows |
| S4 Alternative rapid-progression definition | 2379 | 592 | 0.77 | 0.821 | 0.564 | 0.455 | 0.569 | 8.3% of participants change class under this definition |

Notes: All rows are for the XGBoost model. The main analysis is repeated under a complete-case restriction, under multiple imputation, under a collapsed three-class outcome, and under an alternative definition of rapid progression; the ten single imputed datasets contributing to the Rubin-pooled row are listed immediately beneath it rather than in a separate panel. The multiple-imputation model was fitted on the training rows only and applied to the test rows, so test-set information did not contribute to it; continuous variables were imputed by predictive mean matching and binary variables by logistic regression, with ten imputations, and the result reproduces the main analysis to within 0.002 Macro-AUC. The complete-case analysis retained 440 of 2,971 participants (14.8%), leaving 361 for training and 79 for testing

**Table S20. Impact of class-imbalance handling strategies on XGBoost performance.**

| **Strategy** | **N training after resampling** | **Class counts after** | **Macro-AUC (CV)** | **Macro-AUC (test)** | **Micro-AUC (test)** | **Overall accuracy** | **Balanced accuracy** | **Macro-F1** | **Weighted F1** | **Kappa** | **Brier** | **Sens., Rapid Prog.** | **PPV, Rapid Prog.** | **AUC, Rapid Prog.** | **Sens., Improvement** | **F1, Improvement** |
| --- | --- | --- | --- | --- | --- | --- | --- | --- | --- | --- | --- | --- | --- | --- | --- | --- |
| No adjustment | 2379 | 188 / 1320 / 524 / 347 | 0.772 | 0.791 | 0.859 | 0.642 | 0.409 | 0.416 | 0.59 | 0.322 | 0.497 | 0.36 | 0.738 | 0.815 | 0 | 0 |
| Inverse-frequency weighting | 2379 | 188 / 1320 / 524 / 347 | 0.766 | 0.785 | 0.809 | 0.544 | 0.516 | 0.483 | 0.561 | 0.317 | 0.589 | 0.43 | 0.514 | 0.823 | 0.489 | 0.309 |
| SMOTE | 5280 | 1320 / 1320 / 1320 / 1320 | 0.765 | 0.777 | 0.847 | 0.627 | 0.442 | 0.462 | 0.603 | 0.336 | 0.52 | 0.384 | 0.673 | 0.829 | 0.106 | 0.143 |
| SMOTEENN | 4059 | 1319 / 280 / 1178 / 1282 | 0.75 | 0.757 | 0.657 | 0.355 | 0.474 | 0.356 | 0.341 | 0.185 | 0.76 | 0.535 | 0.348 | 0.794 | 0.574 | 0.241 |
| ADASYN | 5250 | 1306 / 1320 / 1328 / 1296 | 0.766 | 0.778 | 0.848 | 0.622 | 0.439 | 0.461 | 0.596 | 0.324 | 0.521 | 0.36 | 0.646 | 0.822 | 0.128 | 0.185 |
| Random undersampling | 752 | 188 / 188 / 188 / 188 | 0.743 | 0.745 | 0.723 | 0.443 | 0.507 | 0.432 | 0.461 | 0.231 | 0.675 | 0.547 | 0.49 | 0.802 | 0.617 | 0.279 |
| Balanced random forest | 2379 | 188 / 188 / 188 / 188 | 0.75 | 0.761 | 0.805 | 0.557 | 0.479 | 0.475 | 0.569 | 0.301 | 0.623 | 0.419 | 0.61 | 0.803 | 0.34 | 0.237 |
| Cost-sensitive thresholds (post hoc) | 2379 | 188 / 1320 / 524 / 347 | NA | 0.778 | 0.53 | 0.25 | 0.468 | 0.275 | 0.183 | 0.141 | 0.896 | 0.547 | 0.346 | 0.823 | 0.872 | 0.222 |

**Abbreviations:** ADASYN, adaptive synthetic sampling; AUC, area under the receiver operating characteristic curve; CV, cross-validation; F1, F1-score; SMOTE, synthetic minority oversampling technique; SMOTEENN, SMOTE combined with edited nearest neighbours.

**Notes:** Eight strategies were compared with the XGBoost configuration held fixed, so that only the strategy varied. Resampling was applied inside the training folds only, so no synthetic or discarded observation entered a validation or test fold, and all strategies were evaluated on the same independent test set (n = 592). Macro-AUC ranged from 0.745 to 0.791 while the class-specific consequences differed sharply: making no adjustment gave the nominally highest Macro-AUC and the highest overall accuracy while assigning no participant at all to the Improvement class, so its sensitivity and F1 for that class were both zero. The synthetic oversampling methods did not improve on inverse-frequency weighting; SMOTEENN raised minority-class sensitivity but had the lowest Macro-F1 and Kappa of the resampling strategies. Cost-sensitive thresholds were applied post hoc to the fitted model, so no cross-validated estimate is available for that row. The inverse-frequency weighting row is a refit of the primary configuration and may differ from the main-text table in the third decimal place, because XGBoost is not bit-reproducible across threads.

**Table S21. Learning-curve convergence and apparent optimism at the full training-set size.**

| **Model** | **Fraction of training set** | **N train** | **Train Macro-AUC, mean** | **Train Macro-AUC, SD** | **CV Macro-AUC, mean** | **CV Macro-AUC, SD** | **Test Macro-AUC, mean** | **Test Macro-AUC, SD** | **Optimism, mean** | **CV gain, 50% to 100%** |
| --- | --- | --- | --- | --- | --- | --- | --- | --- | --- | --- |
| GBM | 0.2 | 474 | 0.9981 | 7e-04 | 0.6958 | 0.0329 | 0.7007 | 0.0187 | 0.3024 |  |
|  | 0.35 | 830 | 0.9797 | 0.0017 | 0.693 | 0.0242 | 0.7193 | 0.0158 | 0.2867 |  |
|  | 0.5 | 1189 | 0.9642 | 0.0014 | 0.7266 | 0.0117 | 0.7568 | 0.0039 | 0.2376 |  |
|  | 0.65 | 1545 | 0.9448 | 0.0023 | 0.7414 | 0.0102 | 0.7585 | 0.0072 | 0.2034 |  |
|  | 0.8 | 1902 | 0.9309 | 0.0024 | 0.7399 | 0.0059 | 0.7683 | 0.0055 | 0.191 |  |
|  | 1 | 2379 | 0.9163 | 7e-04 | 0.7593 | 0.0024 | 0.7783 | 0.0024 | 0.157 | 0.0327 |
| Multinomial LR | 0.2 | 474 | 0.8268 | 0.0183 | 0.7284 | 0.0253 | 0.7353 | 0.0042 | 0.0984 |  |
|  | 0.35 | 830 | 0.7833 | 0.0155 | 0.7247 | 0.0212 | 0.7506 | 0.0076 | 0.0586 |  |
|  | 0.5 | 1189 | 0.7766 | 0.0082 | 0.7395 | 0.0136 | 0.7648 | 0.0016 | 0.0371 |  |
|  | 0.65 | 1545 | 0.7749 | 0.0088 | 0.7511 | 0.0078 | 0.7637 | 0.0017 | 0.0238 |  |
|  | 0.8 | 1902 | 0.7641 | 0.0053 | 0.7434 | 0.0079 | 0.7646 | 0.0022 | 0.0207 |  |
|  | 1 | 2379 | 0.7652 | 0 | 0.7509 | 0.0015 | 0.768 | 0 | 0.0142 | 0.0114 |
| RF | 0.2 | 474 | 0.9965 | 0.0015 | 0.7236 | 0.0314 | 0.7294 | 0.0097 | 0.2729 |  |
|  | 0.35 | 830 | 0.9961 | 5e-04 | 0.7221 | 0.0263 | 0.7423 | 0.0084 | 0.2741 |  |
|  | 0.5 | 1189 | 0.9964 | 4e-04 | 0.7492 | 0.011 | 0.7661 | 0.0069 | 0.2471 |  |
|  | 0.65 | 1545 | 0.9954 | 2e-04 | 0.7584 | 0.0048 | 0.7606 | 0.0059 | 0.2371 |  |
|  | 0.8 | 1902 | 0.9958 | 2e-04 | 0.7582 | 0.0056 | 0.7666 | 0.008 | 0.2376 |  |
|  | 1 | 2379 | 0.9957 | 0 | 0.7684 | 0.0022 | 0.7725 | 0.0016 | 0.2273 | 0.0192 |
| SVM | 0.2 | 474 | 0.9173 | 0.0216 | 0.6952 | 0.0293 | 0.7002 | 0.0074 | 0.2221 |  |
|  | 0.35 | 830 | 0.905 | 0.0107 | 0.715 | 0.0196 | 0.715 | 0.0158 | 0.19 |  |
|  | 0.5 | 1189 | 0.9001 | 0.0052 | 0.7209 | 0.0089 | 0.7398 | 0.0051 | 0.1792 |  |
|  | 0.65 | 1545 | 0.8948 | 0.0055 | 0.7378 | 0.0103 | 0.7372 | 0.0086 | 0.1569 |  |
|  | 0.8 | 1902 | 0.8892 | 0.0017 | 0.7328 | 0.0046 | 0.7425 | 0.0041 | 0.1564 |  |
|  | 1 | 2379 | 0.8851 | 5e-04 | 0.7448 | 0.0021 | 0.7493 | 2e-04 | 0.1402 | 0.0239 |
| XGBoost | 0.2 | 474 | 0.9979 | 7e-04 | 0.7036 | 0.038 | 0.7219 | 0.0101 | 0.2944 |  |
|  | 0.35 | 830 | 0.9891 | 8e-04 | 0.7068 | 0.0227 | 0.7362 | 0.0121 | 0.2823 |  |
|  | 0.5 | 1189 | 0.9789 | 0.001 | 0.7399 | 0.01 | 0.7653 | 0.0044 | 0.2391 |  |
|  | 0.65 | 1545 | 0.9646 | 0.0026 | 0.7509 | 0.0107 | 0.7634 | 0.0061 | 0.2136 |  |
|  | 0.8 | 1902 | 0.9533 | 0.0014 | 0.7512 | 0.0048 | 0.7733 | 0.0053 | 0.2021 |  |
|  | 1 | 2379 | 0.9391 | 6e-04 | 0.7649 | 0.0029 | 0.7866 | 0.0024 | 0.1742 | 0.025 |

Notes: Each model was refitted on nested random subsamples of the training set from 20% to 100% of its size, with five repeats at each size, and the training, cross-validated and test Macro-AUC recorded at each size. Optimism is the training value minus the cross-validated value at the same size, so a larger value indicates more overfitting. The convergence summary formerly given as a separate panel is reproduced within these rows: the cross-validated values at 50% and 100% are the CV mean at fractions 0.50 and 1.00, the optimism at the full size is the optimism mean at fraction 1.00, and the only quantity not otherwise readable from the rows, the gain between half and the full training set, is given in the final column on the fraction 1.00 row of each model. That gain was between 0.011 and 0.033, indicating convergence at the available sample size. Optimism remains substantial for the tree ensembles and negligible for the penalised multinomial model. The stacking ensemble is omitted because refitting it at every subsample size was computationally prohibitive.

# 2 Supplementary Figures

**Figure S1.** Participant flow. (A) Development cohort, CHARLS Waves 1 and 3. (B) External validation cohort.

**Figure S2.** CKM syndrome stage transition matrices in the development and validation cohorts. (A) Transition matrix from Wave 1 (2011) to Wave 3 (2015) in the CHARLS development cohort (n = 3,442). (B) Transition matrix from baseline to follow-up assessment in the external hospital validation cohort (N = 291).

**Figure S3.** Micro-averaged, pooled one-versus-rest ROC curves for all six models, reported separately from the macro-averaged curves of Figure 1B.

**Figure S4.** Confusion matrices for all six models in the internal testing set. Rows indicate the predicted trajectory and columns the actual trajectory, with percentages computed within the true class, so that each column sums to 100%.

**Figure S5.** Decision curve analysis for all four trajectory classes.

**Figure S6.** Screening yield: percentage of rapid progressors captured against percentage of the cohort flagged for follow-up.

**Figure S7.** Macro-averaged AUC of the full-predictor and parsimonious models in the internal testing set.

**Figure S8.** Standardised mean differences between the development and external cohorts for every comparable predictor.

**Figure S9.** Distribution of the follow-up interval in the external cohort, against the fixed four-year development interval.

**Figure S10.** Macro-averaged AUC across the sensitivity analyses for missing-data handling and outcome definition.

**Figure S11.** Effect of eight class-imbalance handling strategies on discrimination, balanced accuracy, macro-F1 and rapid-progression sensitivity.

**Figure S12.** Learning curves: training, cross-validated and testing macro-averaged AUC against training-set size, by model.
